# Synthesis of Phthalimide Derivatives and Their Insecticidal Activity against Caribbean Fruit Fly, *Anastrepha suspensa* (Loew)

**DOI:** 10.3390/biom13020361

**Published:** 2023-02-14

**Authors:** Fatih Tok, Xiangbing Yang, Nurhayat Tabanca, Bedia Koçyiğit-Kaymakçıoğlu

**Affiliations:** 1 Department of Pharmaceutical Chemistry, Faculty of Pharmacy, Marmara University, Istanbul 34854, Turkey; 2 Subtropical Horticulture Research Station (SHRS), United States Department of Agriculture-Agricultural Research Service (USDA-ARS), 13601 Old Cutler Rd., Miami, FL 33158, USA

**Keywords:** *Anastrepha suspensa*, Caribbean fruit fly, Caribfly, Tephritidae, fruit fly, toxicity, phthalimide

## Abstract

In this study, thirteen phthalimide derivatives were designed and synthesized. All synthesized compounds were evaluated to determine their potential for inhibitory activities against females of the Caribbean fruit fly, *Anastrepha suspensa* (Loew) (Diptera: Tephritidae). These efforts led to the discovery of three compounds **4a**, **4c**, and **4d** with potent insecticidal activity (LD_50_ range from 0.70 to 1.91 μg/fly). Among these compounds, **4a** exhibited the highest inhibitory potency with 0.70 μg/fly. In addition, in silico models indicated that compound **4a** is less toxic than phthalimide and other precursors. Therefore, our results suggest that **4a** has strong potential as a candidate component for developing a novel environmentally friendly insecticide for control of pest fruit flies.

## 1. Introduction

The class of isoindoline-1,3-diones (e.g., phthalimide) has been identified as a privileged scaffold for designing new drug candidates with a wide range of naturally occurring and bioactive substances [1]. The phthalimide ring has been reported to be a very important substructure in organic chemistry for synthesizing various biologically active molecules [2]. The phthalimides have a high potential to cross different in vivo biological barriers due to the hydrophobic -CON(R)-CO- pharmacophore group in their structures [3,4]. The most important pharmacological effects reported for phthalimide derivatives are antimicrobial, anthelmintic, antimalarial and insecticidal activities [5,6,7]. The minimum inhibitory concentrations (MICs) values of cyclic imide structures could be comparable to clinically used antibiotics [8]. Panek et al. and Zhang et al. have shown in different studies that the phthalimide structure plays an important role in binding to cholinesterase enzymes and showing inhibitory activity [9,10], which is one of the key mechanisms for pest control. Therefore, compounds with phthalimide structure are likely to bind to cholinesterase enzymes of insects and exhibit inhibitory activity. Similarly, there are many literature reports of phthalimide drugs showing insecticidal effects such as tetramethrin, phosmet, and dialifos, or fungicidal effects such as capton, captofol, and folpet [11].

Resistance to existing pesticides develops over time [12]. In addition, the accumulation of pesticides in water resources and food products is another important problem. Therefore, there is a need for safe, environmentally friendly compounds that can easily decompose into nontoxic residues and do not harm humans and beneficial organisms [13]. Based on phthalimide derivatives, many compounds with both excellent insecticidal efficiency and environmentally friendly properties have been synthesized [14]. For example, Zhang et al. synthesized some compounds with phthalimide structure that are potent and environmentally friendly with insecticidal activity against the oriental armyworm, *Mythimna separata* [15]. It has been found by EFSA (European Food Safety Authority) that *N*-hydroxymethyl phthalimide derivatives present low risk to aquatic organisms living on the surface of the water [16].

**Figure 1:**
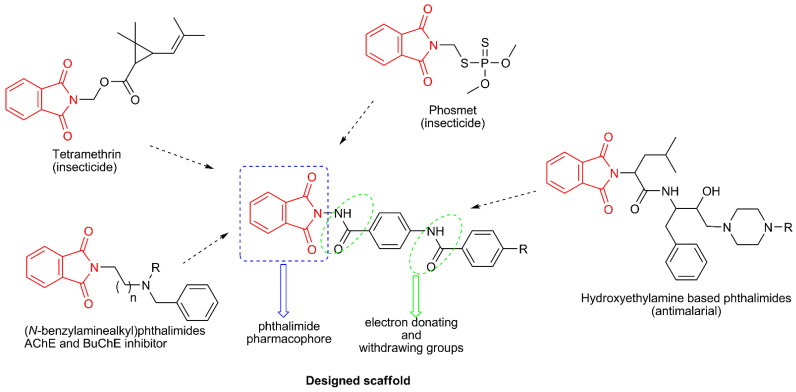
Schematic design strategy of novel molecules based phthalimide.

Tephritid fruit flies are major insect pests that damage fruit crops all over the world [17]. Current pest management relies on lures or bait stations that incorporate insecticide [18]. However, with increasing use of conventional chemical insecticides, resistance has been reported from various tephritid species and locations, thereby creating a need to develop alternatives such as environmentally friendly chemicals to control these pests [19]. The Caribbean fruit fly or Caribfly, *Anastrepha suspensa* (Loew) (Diptera: Tephritidae), is found in North America including Cuba, Jamaica, Hispaniola, Puerto Rico, and the USA and has been established since 1965. In Florida, *A. suspensa* is a quarantine pest of citrus and a major pest of many specialty fruits, particularly guava. Developing new strategies for control of fruit flies using environmentally friendly toxicants are promising approaches for not only improving monitoring tools but also easing the development of pesticide resistance with minimized environmental impact [20,21]. Thus, in this study, we designed and synthesized some new molecules bearing phthalimide structures (Figure 1) to explore their chemical characteristics and biological activities for the control of insect pests. The risk of toxic effects of these compounds on the environment has been explained using ADMETlab and Osiris programs. We investigated the derivatives’ inhibitory activities against female *A. suspensa* and determined the efficacy of those chemicals as toxicants against female adult flies. The potential application of these derivatives as alternative means for control of *A. suspensa* is also discussed.

## 2. Materials and Methods

All chemicals used in this study were purchased from Merck Company and Sigma-Aldrich. Melting points were determined by using the SMP II melting point apparatus (Cole-Parmer Ltd., Staffordshire, UK). All reactions were monitored by TLC and performed on 0.2 mm thick silica gel plates (60 F_254_ Merck). IR spectra were recorded on a Shimadzu FTIR-8400S spectrophotometer (Shimadzu Corp., Kyoto, Japan). ^1^H-NMR and ^13^C-NMR spectra were obtained on a Bruker Avance DPX-400 spectrometer (Bruker Corp., Billerica, MA, USA) operating at 400 MHz and 125 MHz, respectively. Tetramethylsilane as the internal standard and DMSO-*d*_6_ as the solvent were used for NMR spectrums (Figures S1–S39). Elemental analysis was performed on the Thermo Scientific Flash 2000 (Finnegan MAT, USA).

### 2.1. Procedure for the Synthesis of Derivatives ***1***

A mixture of 1 mmol *p*-aminobenzoic acid and 1 mL conc. H_2_SO_4_ was refluxed in 5 mL absolute ethanol for 5 h. After the reaction completed, 10 mL of cold 50% NaOH (*w*/*v*) was added dropwise, and precipitate product was stirred. The product was obtained by filtration and washed with cold water [22]. CAS registry number: 94-09-7.

### 2.2. Procedure for the Synthesis of Derivatives ***2a***–***2m***

Ethyl *p*-aminobenzoate (3 mmol) was dissolved in 10 mL ether. Then 3 mmol substituted benzoyl chloride was added on the reaction dropwise and vigorously stirred until the white precipitate was formed. The precipitate was washed with water until the smell of benzoyl chloride disappeared [23] to obtain derivatives **2a**–**2m**. The structures of **2a**–**2g** and **2j**–**2m** were reported in the literature [24,25,26,27]. CAS registry number: 876534-36-0 for compound **2h**; CAS registry number: 425627-98-1 for **2i**.

### 2.3. Procedure for the Synthesis of Derivatives ***3a***–***3m***

Ethyl 4-(substituted)benzamidobenzoate (10 mmol), hydrazine monohydrate (9 mL), and 20 mL of ethanol were refluxed 110–120 °C for 2 h. The excess solvent was evaporated under vacuum. The product was filtered and washed with cold water. The product was then recrystallized in ethanol to obtain **3a**–**3m** [26]. The structures of **3a**–**3e**, **3l**, and **3m** were reported in the literature [28,29,30,31]. CAS registry number: 315249-21-9 for compound **3k**.

4-Bromo-*N*-(4-(hydrazinecarbonyl)phenyl)benzamide (**3f**)

Yield: 85%, white solid, m.p. = 221.3–221.7 °C. ^1^H-NMR (500 MHz, DMSO-d_6_): *δ* 4.29 (s, 2H, NH_2_), 7.75–8.03 (m, 8H, Ar-H), 10.61 (s, 1H, hydrazide NH), 11.24 (s, 1H, amide NH). ^13^C-NMR (125 MHz, DMSO-d_6_): *δ* 124.35, 125.22, 126.03, 129.20, 129.98, 130.40, 130.54, 131.94, 134.07, 135.89, 143.85, 165.46, 166.80. Anal. calcd. for C_22_H_15_N_3_O_4_: C, 50.32; H, 3.62; N, 12.57. Found: C, 50.88; H, 3.78; N, 12.37.

*N*-(4-(Hydrazinecarbonyl)phenyl)-2-(trifluoromethyl)benzamide (**3g**)

Yield: 70%, beige solid, m.p. = 222.2–222.6 °C. ^1^H-NMR (500 MHz, DMSO-d_6_): *δ* 4.31 (s, 2H, NH_2_), 7.39–7.99 (m, 8H, Ar-H), 10.48 (s, 1H, hydrazide NH), 11.21 (s, 1H, amide NH). ^13^C-NMR (125 MHz, DMSO-d_6_): *δ* 120.02, 124.95, 125.35, 128.78, 130.50, 130.79, 144.10, 144.13, 165.78, 165.83. Anal. calcd. for C_22_H_15_N_3_O_4_: C, 55.73; H, 3.74; N, 13.00. Found: C, 55.20; H, 3.92; N, 13.27.

*N*-(4-(Hydrazinecarbonyl)phenyl)-3-(trifluoromethyl)benzamide (**3h**)

Yield: 75%, beige solid, m.p. = 224.4–225.0 °C. ^1^H-NMR (500 MHz, DMSO-d_6_): *δ* 4.43 (s, 2H, NH_2_), 6.90–8.04 (m, 8H, Ar-H), 10.61 (s, 1H, hydrazide NH), 11.24 (s, 1H, amide NH). Anal. calcd. for C_22_H_15_N_3_O_4_: C, 55.73; H, 3.74; N, 13.00. Found: C, 56.05; H, 3.88; N, 12.87.

*N*-(4-(Hydrazinecarbonyl)phenyl)-4-(trifluoromethoxy)benzamide (**3i**)

Yield: 75%, beige solid, m.p. = 227.7–228.4 °C. ^1^H-NMR (500 MHz, DMSO-d_6_): *δ* 4.30 (s, 2H, NH_2_), 7.89–8.08 (m, 8H, Ar-H), 10.74 (s, 1H, hydrazide NH). ^13^C-NMR (125 MHz, DMSO-d_6_): *δ* 120.11, 125.36, 127.35, 128.75, 129.67, 130.57, 131.20, 136.34, 137.72, 143.74, 165.53, 165.78. Anal. calcd. for C_22_H_15_N_3_O_4_: C, 53.10; H, 3.57; N, 12.39. Found: C, 53.55; H, 3.72; N, 12.55.

*N*-(4-(Hydrazinecarbonyl)phenyl)-4-(trifluoromethylthio)benzamide (**3j**)

Yield: 75%, yellow solid, m.p. = 181.1–181.7 °C. ^1^H-NMR (500 MHz, DMSO-d_6_): *δ* 4.29 (s, 2H, NH_2_), 7.54–8.11 (m, 8H, Ar-H), 10.65 (s, 1H, hydrazide NH), 11.24 (s, 1H, amide NH). ^13^C-NMR (125 MHz, DMSO-d_6_): *δ* 120.08, 121.19, 125.25, 130.55, 130.70, 134.16, 143.85, 151.12, 165.25, 165.79. Anal. calcd. for C_22_H_15_N_3_O_4_: C, 50.70; H, 3.40; N, 11.83. Found: C, 51.25; H, 3.22; N, 11.65.

### 2.4. Procedure for the Synthesis of Derivatives ***4a***–***4m***

*N*-(4-(Hydrazinecarbonyl)phenyl)substitutedbenzamide (10 mmol), phthalic anhydride (10 mmol), and glacial acetic acid (10 mL) were added to a round bottom flask. The mixture was heated under reflux for 8 h. After cooling of the reaction mixture, the solid was gathered and crystallized from ethanol to obtain **4a**–**4m [32]**.

Our results showed that compounds **4a**–**4m** were successfully synthesized, and the detailed chemical information of each derivative is described below: 

4-Benzamido-*N*-(1,3-dioxoisoindolin-2-yl)benzamide (**4a**)

Yield: 79%, beige solid, m.p. = 213.9–214.3 °C. FTIR (ATR, cm^−1^): 3379, 3317 (N-H), 3066 (=C-H), 1788, 1728 (C=O imide), 1687, 1660 (C=O amide and hydrazide), 1593, 1521 (C=C), 1325 (C-N). ^1^H-NMR (400 MHz, DMSO-d_6_): *δ* 7.52–7.61 (m, 3H, Ar-H), 7.94–8.02 (m, 10H, Ar-H), 10.58 (s, 1H, hydrazide NH), 11.24 (s, 1H, amide NH). ^13^C-NMR (100 MHz, DMSO-d_6_): *δ* 120.12, 124.35, 125.84, 128.29, 128.94, 129.22, 129.98, 132.38, 135.07, 135.88, 143.74, 165.32, 165.97, 166.52. Anal. calcd. for C_22_H_15_N_3_O_4_: C, 68.57; H, 3.92; N, 10.90. Found: C, 67.98; H, 3.15; N, 9.90.

*N*-(1,3-Dioxoisoindolin-2-yl)-4-(4-fluorobenzamido)benzamide (**4b**)

Yield: 83%, beige solid, m.p. = 225.5–225.9 °C. FTIR (ATR, cm^−1^): 3389, 3273 (N-H), 3057 (=C-H), 1791, 1725 (C=O imide), 1685, 1662 (C=O amide and hydrazide), 1593, 1526 (C=C), 1325 (C-N). ^1^H-NMR (400 MHz, DMSO-d_6_): *δ* 7.36–7.41 (t, 1H, Ar-H), 7.81–8.08 (m, 11H, Ar-H), 10.59 (s, 1H, hydrazide NH), 11.22 (s, 1H, amide NH). ^13^C-NMR (100 MHz, DMSO-d_6_): *δ* 115.82, 116.03, 119.96, 120.14, 124.09, 124.36, 125.38, 125.89, 129.21, 129.97, 131.04, 131.14, 131.50, 135.54, 135.91, 143.61, 165.37, 165.96. Anal. calcd. for C_22_H_14_FN_3_O_4_: C, 65.51; H, 3.50; N, 10.42. Found: C, 66.01; H, 3.98; N, 10.91.

*N*-(1,3-Dioxoisoindolin-2-yl)-4-(4-chlorobenzamido)benzamide (**4c**)

Yield: 85%, beige solid, m.p. = 210.1–210.6 °C. FTIR (ATR, cm^−1^): 3373, 3319 (N-H), 3047 (=C-H), 1786, 1725 (C=O imide), 1695, 1658 (C=O amide and hydrazide), 1593, 1529 (C=C), 1327 (C-N). ^1^H-NMR (400 MHz, DMSO-d_6_): *δ* 7.60–7.65 (m, 2H, Ar-H), 7.95–8.05 (m, 10H, Ar-H), 10.59 (s, 1H, hydrazide NH), 11.27 (s, 1H, amide NH). ^13^C-NMR (100 MHz, DMSO-d_6_): *δ* 120.03, 120.20, 124.34, 126.00, 127.92, 128.72, 129.02, 129.23, 129.97, 130.21, 130.26, 133.73, 133.80, 135.88, 137.15, 137.24, 142.58, 143.51, 165.29, 165.44, 165.96, 169.08. Anal. calcd. for C_22_H_14_ClN_3_O_4_: C, 62.94; H, 3.36; N, 10.01. Found: C, 63.58; H, 3.80; N, 9.57.

*N*-(1,3-Dioxoisoindolin-2-yl)-4-(4-methylbenzamido)benzamide (**4d**)

Yield: 80%, white solid, m.p. = 236.6–237.1 °C. FTIR (ATR, cm^−1^): 3290, 3244 (N-H), 3024 (=C-H), 1788, 1734 (C=O imide), 1697, 1647 (C=O amide and hydrazide), 1595, 1521 (C=C), 1325 (C-N). ^1^H-NMR (400 MHz, DMSO-d_6_): *δ* 2.41 (s, 3H, CH_3_), 7.30–7.37 (m, 2H, Ar-H), 7.95–8.00 (m, 10H, Ar-H), 10.48 (s, 1H, hydrazide NH), 11.25 (s, 1H, amide NH). ^13^C-NMR (100 MHz, DMSO-d_6_): *δ* 21.51, 119.90, 119.99, 120.07, 124.35, 125.70, 127.63, 128.28, 128.33, 128.66, 129.17, 129.47, 129.97, 130.52, 132.13, 132.21, 135.90, 142.38, 142.49, 142.86, 143.81, 165.30, 165.97, 166.28, 169.06. Anal. calcd. for C_23_H_17_N_3_O_4_: C, 69.17; H, 4.29; N, 10.52. Found: C, 70.50; H, 4.01; N, 10.92.

*N*-(1,3-Dioxoisoindolin-2-yl)-4-(4-methoxybenzamido)benzamide (**4e**)

Yield: 75%, white solid, m.p. = 231.1–231.8 °C. FTIR (ATR, cm^−1^): 3371, 3304 (N-H), 3037 (=C-H), 2978, 2845 (C-H), 1793, 1734 (C=O imide), 1697, 1643 (C=O amide and hydrazide), 1591, 1525 (C=C), 1323 (C-N). ^1^H-NMR (400 MHz, DMSO-d_6_): *δ* 3.86 (s, 3H, OCH_3_), 7.07–7.11 (m, 2H, Ar-H), 7.94–8.02 (m, 10H, Ar-H), 10.37 (s, 1H, hydrazide NH), 11.25 (s, 1H, amide NH). ^13^C-NMR (100 MHz, DMSO-d_6_): *δ* 21.08, 119.16, 119.99, 120.15, 121.22, 121.72, 124.31, 126.13, 127.96, 128.72, 129.23, 129.99, 130.67, 130.72, 134.20, 134.26, 135.85, 142.55, 143.45, 151.12, 165.17, 165.32, 165.97, 169.07. Anal. calcd. for C_23_H_17_N_3_O_5_: C, 66.50; H, 4.12; N, 10.12. Found: C, 67.04; H, 4.58; N, 10.63.

*N*-(1,3-Dioxoisoindolin-2-yl)-4-(4-bromobenzamido)benzamide (**4f**)

Yield: 75%, white solid, m.p. = 203.3–203.8 °C. FTIR (ATR, cm^−1^): 3371, 3317 (N-H), 3032 (=C-H), 1786, 1728 (C=O imide), 1691, 1647 (C=O amide and hydrazide), 1595, 1525 (C=C), 1327 (C-N). ^1^H-NMR (400 MHz, DMSO-d_6_): *δ* 7.55–8.17 (m, 12H, Ar-H), 10.61 (s, 1H, hydrazide NH), 11.25 (s, 1H, amide NH). ^13^C-NMR (100 MHz, DMSO-d_6_): *δ* 120.02, 120.19, 124.35, 126.00, 126.11, 126.21, 127.92, 128.72, 129.23, 129.97, 130.38, 130.42, 131.96, 134.08, 134.16, 135.89, 142.56, 143.49, 165.28, 165.43, 165.96, 169.07. Anal. calcd. for C_22_H_14_BrN_3_O_4_: C, 56.91; H, 3.04; N, 9.05. Found: C, C, 56.12; H, 3.22; N, 9.40.

*N*-(1,3-Dioxoisoindolin-2-yl)-4-(4-(trifluoromethylthio)benzamido)benzamide (**4g**)

Yield: 70%, beige solid, m.p. = 213.7–213.9 °C. FTIR (ATR, cm^−1^): 3335, 3290 (N-H), 3064 (=C-H), 1790, 1737 (C=O imide), 1693, 1651 (C=O amide and hydrazide), 1593, 1521 (C=C), 1323 (C-N). ^1^H-NMR (400 MHz, DMSO-d_6_): *δ* 7.88–8.10 (m, 12H, Ar-H), 10.70 (s, 1H, hydrazide NH), 11.27 (s, 1H, amide NH). ^13^C-NMR (100 MHz, DMSO-d_6_): *δ* 119.99, 120.17, 124.36, 126.13, 127.25, 127.33, 128.05, 128.45, 128.74, 129.25, 129.65, 130.59, 131.51, 135.91, 136.39, 137.74, 142.45, 143.38, 165.25, 165.45, 165.96, 169.06. Anal. calcd. for C_23_H_14_F_3_N_3_O_4_S: C, 56.91; H, 2.91; N, 8.66. Found: C, 57.33; H, 2.84; N, 9.11.

*N*-(1,3-Dioxoisoindolin-2-yl)-4-(4-(trifluoromethoxy)benzamido)benzamide (**4h**)

Yield: 77%, beige solid, m.p. = 229.1–229.7 °C. FTIR (ATR, cm^−1^): 3345, 3242 (N-H), 3034 (=C-H), 1797, 1720 (C=O imide), 1676, 1645 (C=O amide and hydrazide), 1595, 1521 (C=C), 1325 (C-N). ^1^H-NMR (400 MHz, DMSO-d_6_): *δ* 7.56 (s, 2H, Ar-H), 7.96–8.04 (m, 10H, Ar-H), 10.65 (s, 1H, hydrazide NH), 11.28 (s, 1H, amide NH). ^13^C-NMR (100 MHz, DMSO-d_6_): *δ* 119.15, 120.03, 120.77, 121.71, 124.30, 125.22, 126.04, 126.16, 127.95, 128.72, 129.23, 129.99, 130.23, 130.52, 134.13, 135.83, 142.56, 143.50, 165.22, 165.78, 165.94, 169.07. Anal. calcd. for C_23_H_14_F_3_N_3_O_5_: C, 58.85; H, 3.01; N, 8.95. Found: C, 58.22; H, 2.74; N, 8.01.

*N*-(4-(1,3-Dioxoisoindolin-2-ylcarbamoyl)phenyl)-2-(trifluoromethyl)benzamide (**4i**)

Yield: 72%, beige solid, m.p. = 266.9–267.6 °C. FTIR (ATR, cm^−1^): 3372, 3318 (N-H), 3082 (=C-H), 1788, 1728 (C=O imide), 1681, 1647 (C=O amide and hydrazide), 1593, 1521 (C=C), 1325 (C-N). ^1^H-NMR (400 MHz, DMSO-d_6_): *δ* 7.62–8.10 (m, 12H, Ar-H), 10.81 (s, 1H, hydrazide NH), 11.28 (s, 1H, amide NH). ^13^C-NMR (100 MHz, DMSO-d_6_): *δ* 119.54, 122.86, 124.34, 125.58, 125.84, 126.16, 126.47, 126.87, 126.92, 128.30, 128.88, 129.04, 129.40, 129.96, 130.80, 133.18, 135.90, 136.21, 143.26, 165.23, 165.95, 166.50. Anal. calcd. for C_23_H_14_F_3_N_3_O_4_: C, 60.93; H, 3.11; N, 9.27. Found: C, 61.77; H, 3.33; N, 8.88.

*N*-(4-(1,3-Dioxoisoindolin-2-ylcarbamoyl)phenyl)-3-(trifluoromethyl)benzamide (**4j**)

Yield: 77%, beige solid, m.p. = 274.0–274.5 °C. FTIR (ATR, cm^−1^): 3377, 3282 (N-H), 3064 (=C-H), 1791, 1734 (C=O imide), 1683, 1647 (C=O amide and hydrazide), 1591, 1525 (C=C), 1309 (C-N). ^1^H-NMR (400 MHz, DMSO-d_6_): *δ* 7.42–7.45 (m, 2H, Ar-H), 7.75–8.04 (m, 10H, Ar-H), 10.55 (s, 1H, hydrazide NH), 11.23 (s, 1H, amide NH). ^13^C-NMR (100 MHz, DMSO-d_6_): *δ* 120.17, 123.06, 124.36, 124.80, 125.77, 126.19, 127.82, 128.12, 128.74, 128.86, 129.24, 129.96, 130.15, 130.58, 132.44, 135.90, 135.99, 143.32, 165.01, 165.42, 165.95, 169.07. Anal. calcd. for C_23_H_14_F_3_N_3_O_4_: C, 60.93; H, 3.11; N, 9.27. Found: C, 61.84; H, 3.39; N, 9.79.

*N*-(1,3-Dioxoisoindolin-2-yl)-4-(3-methylbenzamido)benzamide (**4k**)

Yield: 80%, beige solid, m.p. = 232.3–232.7 °C. FTIR (ATR, cm^−1^): 3284, 3254 (N-H), 3039 (=C-H), 1791, 1734 (C=O imide), 1689, 1645 (C=O amide and hydrazide), 1595, 1521 (C=C), 1329 (C-N). ^1^H-NMR (400 MHz, DMSO-d_6_): *δ* 2.01 (s, 3H, CH_3_), 7.79–8.23 (m, 12H, Ar-H), 10.79 (s, 1H, hydrazide NH), 11.26 (s, 1H, amide NH). ^13^C-NMR (100 MHz, DMSO-d_6_): *δ* 21.43, 119.90, 120.07, 124.35, 125.40, 125.76, 127.69, 128.68, 128.83, 129.19, 129.97, 132.87, 132.96, 135.06, 135.90, 138.26, 142.82, 143.76, 165.29, 165.45, 165.97, 169.06. Anal. calcd. for C_23_H_17_N_3_O_4_: C, 69.17; H, 4.29; N, 10.52. Found: C, 70.00; H, 4.65; N, 9.99.

*N*-(4-(1,3-Dioxoisoindolin-2-ylcarbamoyl)phenyl)-4-cyanobenzamide (**4l**)

Yield: 75%, orange solid, m.p. = 261.0–261.7 °C. FTIR (ATR, cm^−1^): 3252 (N-H), 3039 (=C-H), 1791, 1732 (C=O imide), 1680, 1646 (C=O amide and hydrazide), 1593, 1518 (C=C), 1321 (C-N). ^1^H-NMR (400 MHz, DMSO-d_6_): *δ* 7.63–8.20 (m, 12H, Ar-H), 10.76 (s, 1H, hydrazide NH), 11.40 (s, 1H, amide NH). ^13^C-NMR (100 MHz, DMSO-d_6_): *δ* 118.76, 119.98, 120.04, 120.28, 124.35, 126.01, 127.45, 128.23, 128.76, 129.10, 129.96, 130.76, 132.99, 135.09, 135.89, 138.05, 139.12, 142.76, 143.69, 165.01, 165.48, 165.97, 169.11. Anal. calcd. for C_23_H_14_N_4_O_4_: C, 67.31; H, 3.44; N, 13.65. Found: C, 68.21; H, 3.89; N, 12.95.

*N*-(1,3-Dioxoisoindolin-2-yl)-4-(4-nitrobenzamido)benzamide (**4m**)

Yield: 80%, yellow solid, m.p. = 256.8–257.4 °C. FTIR (ATR, cm^−1^): 3252 (N-H), 3047 (=C-H), 1788, 1737 (C=O imide), 1693, 1645 (C=O amide and hydrazide), 1593, 1525 (C=C), 1325 (C-N). ^1^H-NMR (400 MHz, DMSO-d_6_): *δ* 7.57–8.25 (m, 12H, Ar-H), 10.67 (s, 1H, hydrazide NH), 11.20 (s, 1H, amide NH). ^13^C-NMR (100 MHz, DMSO-d_6_): *δ* 119.91, 120.09, 120.32, 124.36, 125.91, 126.34, 128.26, 128.89, 129.04, 129.84, 130.61, 131.99, 134.44, 135.32, 135.90, 142.27, 143.87, 165.29, 165.48, 165.97, 169.32. Anal. calcd. for C_22_H_14_N_4_O_6_: C, 61.40; H, 3.28; N, 13.02. Found: C, 60.66; H, 2.98; N, 13.98.

### 2.5. In Silico Prediction of Toxicity Risk of Compounds

In this section, the mutagenic, tumorigenic, reproductive, and irritant effect of compound **4a**, which was chosen as a prototype, and its effects on the environment, are investigated with the OSIRIS property explorer (Data warrior), with parameters such as BCF and IGC_50_ with ADMETlab server (https://admetmesh.scbdd.com/, accessed on 4 December 2022).

### 2.6. Toxicity of Derivatives ***4a***–***4m*** against Female Adult Anastrepha Suspensa

To understand the biological activities of phthalimide derivatives **4a**–**4m**, topical bioassays using thoracic application to adult female *Anastrepha suspensa* were conducted to determine the toxicities of these derivatives under laboratory conditions at 26 ± 1 °C, 70 ± 5% RH, and 12:12 L:D photoperiod in the toxicology laboratory, Subtropical Horticultural Research Station, USDA-ARS, Miami, FL, USA. 

To begin with the bioassay, a stock solution of each derivative was first prepared by dissolving 100 mg of each compound (**4a**–**4m**) in 1 mL of dimethyl sulfoxide (DMSO) to establish a 100 mg/mL solution. To evaluate the toxicities of the derivatives, a serial dilution of stock solution was then prepared with acetone to establish 0.1, 1, 10, 50, and 75 µg/µL solutions, and each dilution was used in topical bioassays.

To prepare the female adult *A. suspensa* for the topical bioassay, pupae of *A. suspensa* were collected from rearing cages in the insectary and placed in a tray inside a screen cage (30 cm × 30 cm × 30 cm) under the laboratory conditions to collect newly emerged female adults. After adult emergence, female adults (<3 d old) were then collected into a plastic vial (3 cm in diameter × 8 cm in height) using an aspirator. Female adults in the vial were first chilled at −10 °C in a refrigerator for 5 min to calm the flies, and calmed flies were then removed from the refrigerator to a petri dish for the topical application. On each fly, a repeating dispenser equipped with gastight and microliter syringe (50 µL) (PB600, Hamilton Company, Reno, NV, USA) was used to apply 1 µL dilution at each concentration of each derivative on the dorsal thorax of the calmed adult flies. After topical application, the adult flies were immediately transferred into a plastic cup (6 cm in diameter × 7.4 cm in height) and covered with a mesh screen for post-treatment observation. A block of sugar and yeast hydrolysate mixture (4:1 per weight) (1 cm^3^) and a block of water agar (1 cm^3^) were placed on top of the mesh screen to supply the food and water for tested flies. To remove the chilling effect from the treatment, only adult flies recovered from chill were used for the experiment after topical bioassay. After 24 h, numbers of live and dead flies were documented, and mortality of *A. suspensa* in each treatment was calculated. Untreated female adults and those treated with acetone alone were used as controls. To screen the efficacy of the derivatives (**4a**–**4m**), 1 µg of each chemical was topically applied to 10 adult female fly to document the mortality following the above-mentioned procedure, and the mortality data were documented and showed that, except for **4a**, **4c**, and **4d**, all other derivatives yielded < 20% of mortality (Table S1). Thus, **4a**, **4c**, and **4d** were then continued to be evaluated for toxicities. For each dilution of **4a**, **4c**, and **4d**, 10~15 female adult flies were treated, and for control treatment, 10 female adult flies were treated with acetone. Each treatment was replicated 3 times. In total, 578 female flies were used in the evaluation of all three derivatives.

### 2.7. Statistical Analysis

Median lethal dose (LD_50_) of *A. suspensa* in toxicity bioassays were calculated based on the mortality data for each phthalimide derivatives. Mortality data for each treatment were first corrected by mortalities in the untreated control using Abbott’s formula [33] prior to the analysis. A probit analysis was then used to calculate the lethal dose corresponding to a 50% reduction (LD_50_) in the *A. suspensa*’s survival based on the regression curve. The statistical analysis was performed using SAS version 9.4 [34].

## 3. Results and Discussion

### 3.1. Chemistry

**Scheme 1:**
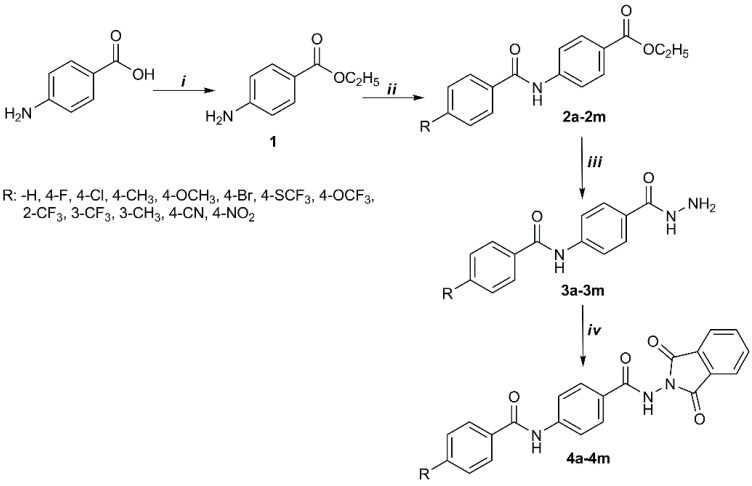
Synthetic pathway of target compounds. (i) Ethanol, H_2_SO_4_; (ii) ether, substituted benzoyl chloride; (iii) ethanol, hydrazine monohydrate; (iv) acetic acid, phthalic anhydride.

The schematic pathway for synthesis of the target compounds is shown in Scheme 1. The target compounds were obtained through four steps of the process. Firstly, ethyl *p*-aminobenzoate (**1**) was obtained by the esterification reaction of *p*-aminobenzoic acid and ethanol in acidic medium. Secondly, amide derivatives (**2a**–**2m**) were prepared with ethyl *p*-aminobenzoate (**1**) and substituted benzoyl chloride by the mechanism of the nucleophilic addition. The reaction of ester with hydrazine hydrate gave the corresponding hydrazide derivatives (**3a**–**3m**) according to nucleophilic addition after the third step. Finally, phthalimide derivatives (**4a**–**4m**) were synthesized from the hydrazide group and phthalic anhydride by dehydrative condensation reaction. IR, ^1^H-NMR, and ^13^C-NMR allowed us to elucidate the structure of the synthesized target compounds (**4a**–**4m**).

When the IR spectrum of **4a**–**4m** compounds was examined, doublet NH peaks in **3a**–**3m** compounds disappeared. The N-H stretching vibrations were detected in the range of 3242–3389 cm^−1^. The carbonyl (C=O) bands of the phthalimide and amide structures were found at 1720–1797 cm^−1^ and 1643–1697 cm^−1^, respectively. The C-H stretching vibrations of aromatic structure appeared within the range of 3024–3082 cm^−1^.

When the ^1^H-NMR spectrum of **4a**–**4m** compounds was examined, the NH peaks of amide and hydrazide structures resonated as singlets within the range of 11.20–11.40 ppm and 10.37–10.81 ppm, respectively. The disappearance of the broad NH peak in the range of 4.28–4.58 ppm of the hydrazide structure belonging to compounds **3a**–**3m** proved the synthesis of the phthalimide structure. 

When the ^13^C-NMR spectrum of **4a**–**4m** compounds was examined, carbonyl (C=O) carbons of amide and phthalimide structures were observed in the range of 165.01–169.32 ppm. All aromatic protons and carbons peaks were consistent with the theoretical values.

### 3.2. Evaluation of In Silico Studies

**Figure 2:**
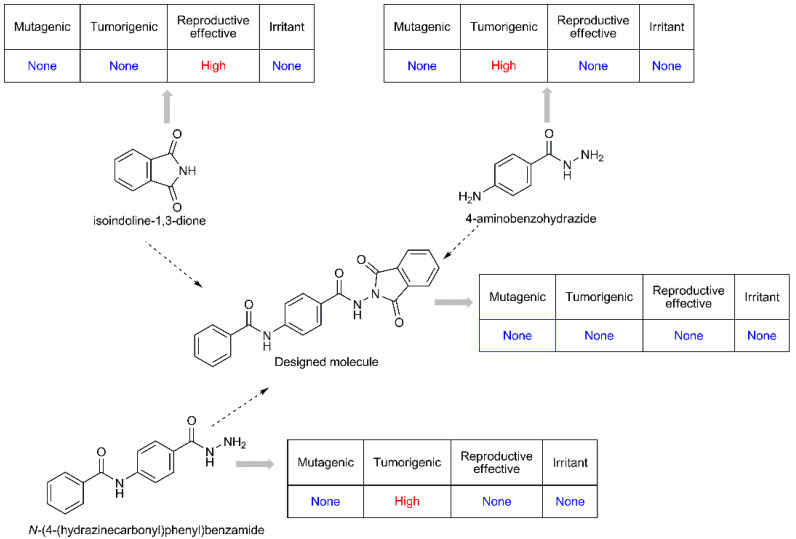
The toxicity profiles of designed molecules.

The pioneering substances of the designed molecules are isoindoline-1,3-dione, 4-aminobenzohydrazide, and *N*-(4-(hydrazinecarbonyl)phenyl)benzamide. When the toxicity profiles of these molecules such as mutagenic, tumorigenic, reproductive, and irritant effect were examined through OSIRIS property explorer [35], it was determined that there is no toxicity of the designed structures. However, the high reproductive effect of isoindoline-1,3-dione and the high tumorigenic effect of 4-aminobenzohydrazide and *N*-(4-(hydrazinecarbonyl)phenyl)benzamide were determined. Therefore, the designed compounds are much safer than the pioneering substances for human health (Figure 2).

Environmental toxicity values such as bioconcentration factor (BCF) and IGC_50_ were evaluated for compound **4a** and the pioneering compounds using ADMETlab server. According to the activity results, **4a** with the highest inhibition value against *A. suspensa* was chosen as the prototype. The BCF is defined as the ratio of the chemical concentration in biota as a result of absorption via the respiratory surface to that in water at a steady state. It is used for considering secondary poisoning potential and assessing risks to human health via the food chain [35,36]. According to the results, compound **4a** was less toxic to the *Tetrahymena pyriformis* compared to the pioneering compounds because of its lower IGC_50_ value (Table 1). It was determined that compound **4a** has less environmental risk than pioneering compounds. Lipophilicity, expressed as the partition coefficient (log P), is generally correlated with biological activity. Compounds with lower log P values are classified as polar, while compounds with higher log P values are considered more lipophilic with better membrane permeability [37]. Compound **4a** had a higher log P value than its precursor compounds. Therefore, it was determined that compound **4a** had higher membrane permeability and therefore higher insecticidal activity.

### 3.3. Toxicity to the Caribbean Fruit Fly

The results showed that phthalimide derivatives **4a**, **4c**, and **4d** had strong toxicity to adult female *A. suspensa*. The median lethal doses (LD_50_) of **4a**, **4c**, and **4d** for *A. suspensa* were 0.70, 1.92, and 0.81 µg/fly, respectively (Table 2). Results showed that an untreated control had a 3.4% adult mortality and had no significant difference between those treated with acetone alone, which resulted in a 6.6% adult mortality. Our data also showed that phthalimide derivative **4a** showed the strongest toxicity against female adult *A. suspensa* than the other two derivatives, with slightly over the half LD_50_ dose of those for **4d** (1.36 µg/fly) and less than half LD_50_ dose of those for **4c**, which was 1.92 µg/fly. This could be due to the different chemical compositions of the three derivatives. The phthalimide and its derivatives have often been used as fungicidal components for control of fungal diseases for crop production [38,39], and the intermediate derivatives such as tetramethrin, phosmet, and dialifos have been used as insecticides for control of insect pests [11]. Therefore, it is speculated that the phthalimide derivatives **4a**, **4c**, and **4d**, may have possessed similar structures, such as the phthalimide pharmacophore (Figure 1), which might play an important role in contributing to insecticidal activities, such as binding to cholinesterase enzymes with inhibitory effect on target insect pests [9,10]. The derivatives **4a**, **4c**, and **4d** showed potential to control tephritid fruit flies, such as the Caribbean fruit fly, *A. suspensa*, which has been an important insect pest for subtropical fruit crops in south Florida. Therefore, these phthalimide derivative chemicals have potential to be used as components in chemical control for tephritid fruit flies, such as including them as components in bait stations or lure traps. However, further studies are needed to analyze the molecular mechanisms underlying the lethal effects of phthalimide derivatives on insect pests (i.e., the binding sites of insect pests and potential synergistic effects of **4a**, **4c**, and **4d**, among the three or with other phthalimide derivatives **4b**, **4e**–**4m**).

## 4. Conclusions

The use of synthetic pesticides is one of the most effective solutions to control pest organisms considered harmful in the current farming systems. However, new active molecules are needed due to the resistance that develops over time and their harmful effects on the environment. Phthalimide, a pharmacophore with different biological activity, is preferred because of its strong insecticidal activity. For this purpose, some new phthalimide derivatives were obtained from the reaction of *N*-(4-(hydrazinecarbonyl)phenyl)benzamide derivatives with phthalic anhydride. The resulting compounds were screened for measuring their insecticidal activity against *A. suspensa*. The insecticidal activity of these substances against the Caribbean fruit fly was investigated for the first time in this study. Among them, compounds **4a, 4c**, and **4d** were found to be the most effective derivatives. In addition to the fact that the phthalimide structure is an environmentally friendly compound, the synthesized compounds were thought to be promising compounds due to their better bioconcentration factor and IGC_50_ values compared to the precursor compounds.

## Figures and Tables

**Table 1:** The environmental toxicity values of compound **4a** and the pioneering compounds.

Compounds	Environmental Toxicity		cLog P
	Bioconcentration Factors *	IGC_50_ **	
Isoindoline-1,3-dione	0.458	2.557	0.536
4-Aminobenzohydrazide	0.495	2.763	−0.753
*N*-(4-(Hydrazinecarbonyl)phenyl)benzamide	0.469	3.337	1.339
Compound **4a**	0.365	3.488	2.674

* The unit of BCF is log10(L/kg). ** 48 h *Tetrahymena pyriformis* IGC_50_ (concentration of the test chemical in water in mg/L that causes 50% growth inhibition to *Tetrahymena pyriformis* after 48 h). The unit of IGC_50_ is −log10[(mg/L)/(1000 × MW)].

**Table 2:** Median lethal dose (LD_50_) of phthalimide derivatives (**4a**, **4c** and **4d**) for the control of the adult female Caribbean fruit fly, *A. suspensa*, under the laboratory conditions.

Chemical	N *	Slop ± SE	LD_50_, µg/fly	χ^2^	df	*p*
**4a**	194	0.85 ± 0.40	0.7015	1.1235	13	0.9999
**4c**	194	0.83 ± 0.38	1.9168	1.6079	13	0.9999
**4d**	190	0.76 ± 0.37	1.3602	0.8145	13	0.9999

* Total number of female adult *A. suspensa* tested.

## Data Availability

Data are contained within the article.

## Supplementary Materials

The following supporting information can be downloaded at: https://www.mdpi.com/article/10.3390/biom13020361/s1, Figures S1–S48 and Table S1.

## References

[1] El-Gohary N.S., Shaaban M.I. (2015). Synthesis, antimicrobial, antiquorum-sensing, and cytotoxic activities of new series of isoindoline-1,3-dione, pyrazolo[5,1-a]isoindole, and pyridine derivatives. Arch. Pharm. Chem. Life Sci..

[2] Rateb H.S., Ahmed H.E.A., Ahmed S., Ihmaid S., Afifi T.H. (2016). Discovery of novel phthalimide analogs: Synthesis, antimicrobial and antitubercular screening with molecular docking studies. EXCLI J..

[3] Bansode T.N., Shelke J.V., Dongre V.G. (2009). Synthesis and antimicrobial activity of some new *N*-acyl substituted phenothiazines. Eur. J. Med. Chem..

[4] Durairaju P., Umarani C., Periyasami G., Vivekanand P.A., Rahaman M. (2021). Synthesis and in vitro antimicrobial evaluation of photoactive multi—block chalcone conjugate phthalimide and 1,8-naphthalimide novolacs. Polymers.

[5] Chi C.L., Xu L., Li J.J., Liu Y., Chen B.Q. (2022). Synthesis, antiproliferative, and antimicrobial properties of novel phthalimide derivatives. Med. Chem. Res..

[6] Akgün H., Karamelekoğlu İ., Berk B., Kurnaz I., Sarıbıyık G., Öktem S., Kocagöz T. (2012). Synthesis and anti mycobacterial activity of some phthalimide derivatives. Bioorg. Med. Chem..

[7] Santos J.L., Yamasaki P.R., Chin C.M., Takashi C.H., Pavan F.R., Leite C.Q.F. (2009). Synthesis and in vitro anti *Mycobacterium tuberculosis* activity of a series of phthalimide derivatives. Bioorg. Med. Chem..

[8] Orzeszko A., Kaminska B., Starosciak B.J. (2002). Synthesis and antimicrobial activity of new adamantane derivatives III. IL Farm..

[9] Panek D., Wieckowska A., Wichur T., Bajda M., Godyn J., Jonczyk J., Mika K., Janockova J., Soukup O., Knez D. (2017). Design, synthesis and biological evaluation of new phthalimide and saccharin derivatives with alicyclic amines targeting cholinesterases, beta-secretase and amyloid beta aggregation. Eur. J. Med. Chem..

[10] Zhang H., Song Q., Yu G., Cao Z., Qiang X., Liu X., Deng Y. (2021). Phthalimide-(*N*-alkylbenzylamine) cysteamide hybrids as multifunctional agents against Alzheimer’s disease: Design, synthesis, and biological evaluation. Chem. Biol. Drug Des..

[11] Pawar N.S., Kapadi U.R., Hundiwale D.G., Kumbhar P.P. (2002). Applications of polymer-supported reactions in the synthesis of pesticides I: Alkylation and acylation of *N*-hydroxy phthalimide. J. Sci. Ind. Res..

[12] South A., Hastings I.M. (2018). Insecticide resistance evolution with mixtures and sequences: A model-based explanation. Malar. J..

[13] Umetsu N., Shirai Y. (2020). Development of novelpesticides in the 21st century. J. Pestic. Sci..

[14] Liu M., Wang Y., Wangyang W.Z., Liu F., Cui Y.L., Duan Y.S., Wang M., Liu S.Z., Rui C.H. (2010). Design, synthesis, and insecticidal activities of phthalamides containing a hydrazone substructure. J. Agric. Food Chem..

[15] Zhang L., Chen R., Li X., Xu X., Xu Z., Cheng J., Wang Y., Li Y., Shao X., Li Z. (2022). Synthesis, insecticidal activities, and 3D-QASR of *N*-pyridylpyrazole amide derivatives containing a phthalimide as potential ryanodine receptor activators. J. Agric. Food Chem..

[16] Anastassiadou M., Arena M., Auteri D., Brancato A., Bura L., Cabrera L.C., Chaideftou E., Chiusolo A., Crivellente F., EFSA (European Food Safety Authority) (2021). Conclusion onthe peer review of the pesticide risk assessment of the active substance phosmet. EFSA J..

[17] Clarke A.R., Measham P.F. (2022). Competition: A Missing component of fruit fly (Diptera: Tephritidae) risk assessment and planning. Insects.

[18] Shelly T., Epsky N., Jang E.B., Reyes-Flores J., Vargas R. (2014). Trapping and the Detection, Control, and Regulation of Tephritid Fruit Flies.

[19] Weldon C.W., Boardman L., Marlin D., Terblanche J.S. (2016). Physiological mechanisms of dehydration tolerance contribute to the invasion potential of *Ceratitis capitata* (Wiedemann) (Diptera: Tephritidae) relative to its less widely distributed congeners. Front. Zool..

[20] Quilici S., Atiama-Nurbel T., Brévault T., Shelly T., Epsky N., Jang E.B., Reyes-Flores J., Vargas R. (2014). Plant odors as fruit fly attractants. Trapping and the Detection, Control, and Regulation of Tephritid Fruit Flies.

[21] Tan K.H., Nishida R., Jang E.B., Shelly T.E., Shelly T., Epsky N., Jang E.B., Reyes-Flores J., Vargas R. (2014). Pheromones, male lures and trapping of tephritid fruit flies. Trapping and the Detection, Control, and Regulation of Tephritid Fruitflies.

[22] Xu S., Pei L., Wang C., Zhang Y.K., Li D., Yao H., Wu X., Chen Z.S., Sun Y., Xu J. (2014). Novel hybrids of natural oridonin-bearing nitrogen mustards as potential anticancer drug candidates. ACS Med. Chem. Lett..

[23] Tok F., Abas B.İ., Çevik Ö., Koçyiğit-Kaymakçıoğlu B. (2020). Design, synthesis and biological evaluation of some new 2-Pyrazoline derivatives as potential anticancer agents. Bioorg. Chem..

[24] Zheng C.H., Yang H., Zhang M., Lu S.H., Shi D., Wang J., Chen X.H., Ren X.H., Liu J., Lv J.G. (2012). Design, synthesis, and activity evaluation of broad-spectrum small-molecule inhibitors of anti-apoptotic Bcl-2 family proteins: Characteristics of broad-spectrum protein binding and its effects on anti-tumor activity. Bioorg. Med. Chem. Lett..

[25] Hassan M.M., Israelian J., Nawar N., Ganda G., Manaswiyoungkul P., Raouf Y.S., Armstrong D., Sedighi A., Olaoye O.O., Erdogan F. (2020). Characterization of conformationally constrained benzanilide scaffolds for potent and selective HDAC8 targeting. J. Med. Chem..

[26] Karakuş S., Rollas S. (2016). Synthesis and antimycobacterial activity of some 2-(4-aminophenyl)-5-substituted amino-1,3,4-thiadiazole derivatives and their coupling products. Marmara Pharm. J..

[27] Verma R.S., Pandey R.K., Kumar P. (1982). Synthesis of Alkyl 4-<<4’-(1,2-Dihydro-5-chloro-2-oxo-3H-indol-3-ylideneamino)-benzoyl>amino>benzoates and Related Compounds. Indian J. Chem. Sect. B..

[28] Seelam N., Shrivastava S.P. (2011). Synthesis and in-vitro activity of some new class of thiazolidinone and their arylidene derivatives. Bull. Korean Chem. Soc..

[29] Sedaghat A., Rezaee E., Hosseini O., Tabatabai S.A. (2020). Para-aminobenzohydrazide derivatives as fatty acid amide hydrolase inhibitors: Design, synthesis and biological evaluation. IJPR-Iran J. Pharm. Res..

[30] Akdağ K., Tok F., Karakuş S., Erdoğan Ö., Çevik Ö., Koçyiğit-Kaymakçıoğlu B. (2022). Synthesis and biological evaluation of some hydrazide-hydrazone derivatives as anticancer agents. Acta Chim. Slov..

[31] Varma R.S., Chauhan S., Prasad C.R. (1983). Synthesis of some 3-<4-(p-carbalkoxyphenylcarbamoyl)phenylaminomethyl>benzothiazolin-2-thiones and related systems as CNS active agents. Indian J. Chem. Sect. B.

[32] Hosny M.A., Zaki Y.H., Mokbel W.A., Abdelhamid A.O. (2019). Synthesis of novel thiazole, pyranothiazole, thiazolo[4,5-*b*]pyridinesandthiazolo[5′,4′:5,6]pyrano[2,3-*d*]pyrimidine derivatives and incorporating isoindoline-1,3-dione group. BMC Chem..

[33] Abbott A.F. (1925). A method of computing the effectiveness of an insecticide. J. Econ. Entomol..

[34] SAS Institute (2020). SAS/STAT, 9.4.

[35] Xiong G., Wu Z., Yi J., Fu L., Yang Z., Hsieh C., Yin M., Zeng X., Wu C., Lu A. (2021). ADMETlab 2.0: An integrated online platform for accurate and comprehensive predictions of ADMET properties. Nucleic Acids Res..

[36] Sodeeq B., Nosakhare I., Isaiah O. (2022). Structure-based discovery of multitarget directed anti-inflammatory p-nitrophenyl hydrazones; molecular docking, drug-likeness, in-silico pharmaco kinetics, and toxicity studies. Biol. Med. Chem..

[37] Tabanca N., Masi M., Epsky N.D., Nocera P., Cimmino A., Kendra P.E., Niogret J., Evidente A. (2019). Laboratory evaluation of natural and synthetic aromatic compounds as potential attractants for male Mediterranean Fruit Fly, *Ceratiti scapitata*. Molecules.

[38] Raina-Fulton R. (2014). A Review of methods for the analysis of orphan and difficult pesticides: Glyphosate, glufosinate, quaternary ammonium and phenoxy acid herbicides, and dithio carbamate and phthalimide fungicides. J. AOAC Int..

[39] Iwasaki J., Hogendoorn K. (2021). Non-insecticide pesticide impacts on bees: A review of methods and reported outcomes. Agric. Ecosyst. Environ..

