# Supplementary material for: Synthesis of Phthalimide Derivatives and Their Insecticidal Activity against Caribbean Fruit Fly, Anastrepha suspensa (Loew)
Source: Biomolecules. 2023 Feb 14;13(2):361. doi: 10.3390/biom13020361 (PMC9953367; doi:10.3390/biom13020361)
Supplement: Supplementary file 1 [file biomolecules-13-00361-s001.zip › biomolecules-2135947-supplementary.pdf]

biomolecules

SupplementaryMaterials

Synthesis of Phthalimide Derivatives and Their Insecticidal Activity Against Caribbean Fruit Fly, *Anastrepha suspensa* (Loew)

Fatih Tok [1], Xiangbing Yang [2], Nurhayat Tabanca [2] and Bedia Koçyiğit-Kaymakçıoğlu [1,*]

[1] Department of Pharmaceutical Chemistry, Faculty of Pharmacy, Marmara University, 34854 4/A, Istanbul, Turkey
[2] Subtropical Horticulture Research Station (SHRS), United States Department of Agriculture-Agricultural Research Service (USDA-ARS), 13601 Old Cutler Rd., Miami, FL 33158, USA
* Correspondence: bkaymakcioglu@marmara.edu.tr; Tel.: +90-(216)-777-5200

LIST OF CONTENTS

Figure S1. IR spectrum for **4a**

Figure S2. ^{1}H-NMR spectrum for **4a** (400 MHz, DMSO-d_6)

Figure S3. ^{13}C-NMR spectrum for **4a** (100 MHz, DMSO-d_6)

Figure S4. IR spectrum for **4b**

Figure S5. ^{1}H-NMR spectrum for **4b** (400 MHz, DMSO-d_6)

Figure S6. ^{13}C-NMR spectrum for **4b** (100 MHz, DMSO-d_6)

Figure S7. IR spectrum for **4c**

Figure S8. ^{1}H-NMR spectrum for **4c** (400 MHz, DMSO-d_6)

Figure S9. ^{13}C-NMR spectrum for **4c** (100 MHz, DMSO-d_6)

Figure S10. IR spectrum for **4d**

Figure S11. ^{1}H-NMR spectrum for **4d** (400 MHz, DMSO-d_6)

Figure S12. ^{13}C-NMR spectrum for **4d** (100 MHz, DMSO-d_6)

Figure S13. IR spectrum for **4e**

Figure S14. ^{1}H-NMR spectrum for **4e** (400 MHz, DMSO-d_6)

Figure S15. ^{13}C-NMR spectrum for **4e** (100 MHz, DMSO-d_6)

Figure S16. IR spectrum for **4f**

Figure S17. ^{1}H-NMR spectrum for **4f** (400 MHz, DMSO-d_6)

Figure S18. ^{13}C-NMR spectrum for **4f** (100 MHz, DMSO-d_6)

Figure S19. IR spectrum for **4g**

Figure S20. ^{1}H-NMR spectrum for **4g** (400 MHz, DMSO-d_6)

Figure S21. ^{13}C-NMR spectrum for **4g** (100 MHz, DMSO-d_6)

Figure S22. IR spectrum for **4h**

Figure S23. ^{1}H-NMR spectrum for **4h** (400 MHz, DMSO-d_6)

Figure S24. ^{13}C-NMR spectrum for **4h** (100 MHz, DMSO-d_6)

Figure S25. IR spectrum for **4i**

Figure S26. ^{1}H-NMR spectrum for **4i** (400 MHz, DMSO-d_6)

Figure S27. ^{13}C-NMR spectrum for **4i** (100 MHz, DMSO-d_6)

Figure S28. IR spectrum for **4j**

Figure S29. ^{1}H-NMR spectrum for **4j** (400 MHz, DMSO-d_6)

Figure S30. ^{13}C-NMR spectrum for **4j** (100 MHz, DMSO-d_6)

Figure S31. IR spectrum for **4k**

Figure S32. ^{1}H-NMR spectrum for **4k** (400 MHz, DMSO-d_6)

Figure S33. ^{13}C-NMR spectrum for **4k** (100 MHz, DMSO-d_6)

Figure S34. IR spectrum for **4l**

Figure S35. ^{1}H-NMR spectrum for **4l** (400 MHz, DMSO-d_6)

Figure S36. ^{13}C-NMR spectrum for **4l** (100 MHz, DMSO-d_6)

Figure S37. IR spectrum for **4m**

Figure S38. ^{1}H-NMR spectrum for **4m** (400 MHz, DMSO-d_6)

Figure S39. ^{13}C-NMR spectrum for **4m** (100 MHz, DMSO-d_6)

Figure S40. ^{1}H-NMR spectrum for **3f** (500 MHz, DMSO-d_6)

Figure S41. ^{13}C-NMR spectrum for **3f** (125 MHz, DMSO-d_6)

Figure S42. ^{1}H-NMR spectrum for **3g** (500 MHz, DMSO-d_6)

Figure S43. ^{13}C-NMR spectrum for **3g** (125 MHz, DMSO-d_6)

Figure S44. ^{1}H-NMR spectrum for **3h** (500 MHz, DMSO-d_6)

Figure S45. ^{1}H-NMR spectrum for **3i** (500 MHz, DMSO-d_6)

Figure S46. ^{13}C-NMR spectrum for **3i** (125 MHz, DMSO-d_6)

Figure S47. ^{1}H-NMR spectrum for **3j** (500 MHz, DMSO-d_6)

Figure S48. ^{13}C-NMR spectrum for **3j** (125 MHz, DMSO-d_6)

Table S1. Screening test to evaluation of derivatives (**4a**-**4m**) on mortality of adult female *A. suspensa* under the laboratory conditions at 26±1°C, 70±5% RH, and 12:12 L:D photoperiod.

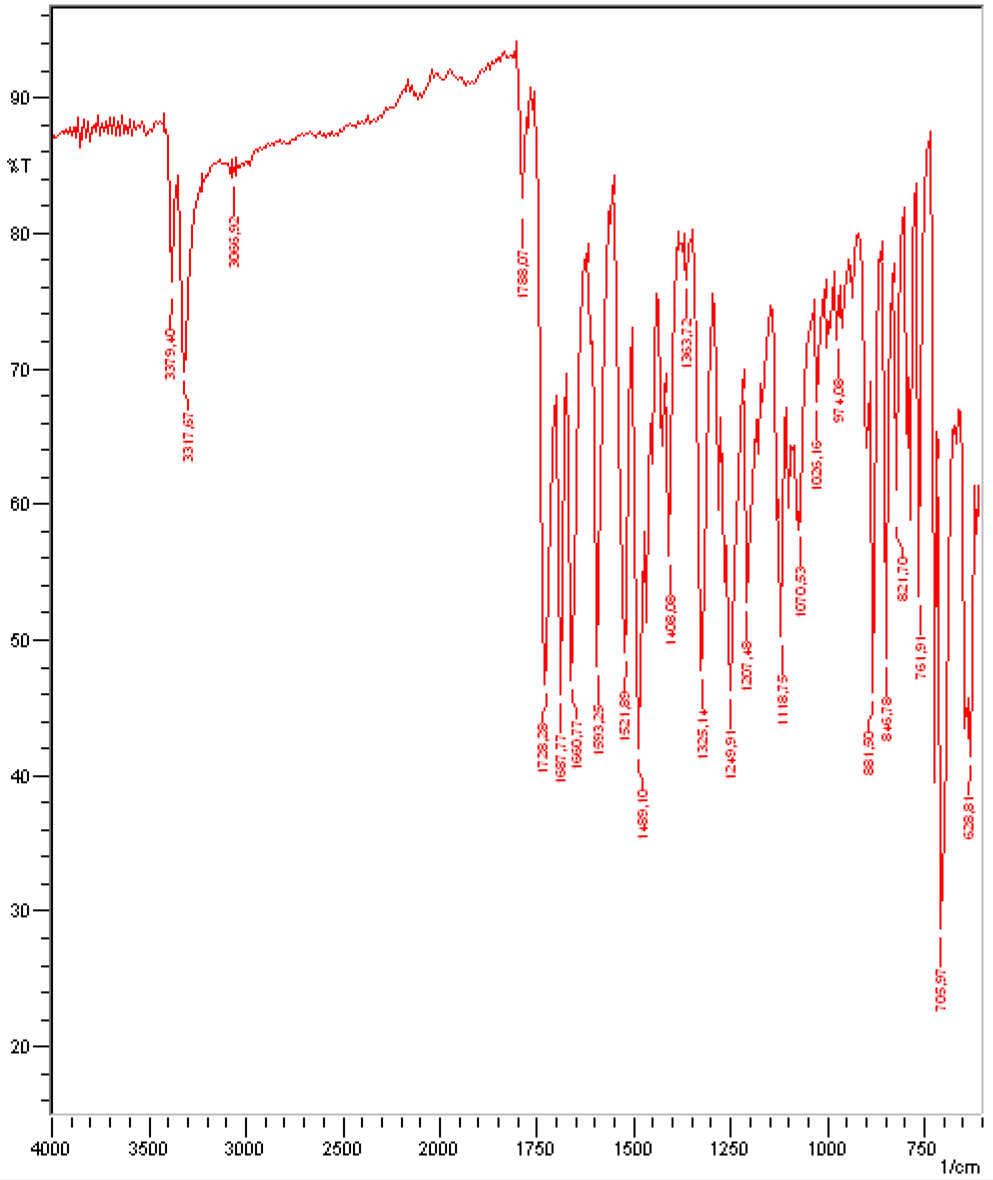

Figure S1. IR spectrum for **4a.**

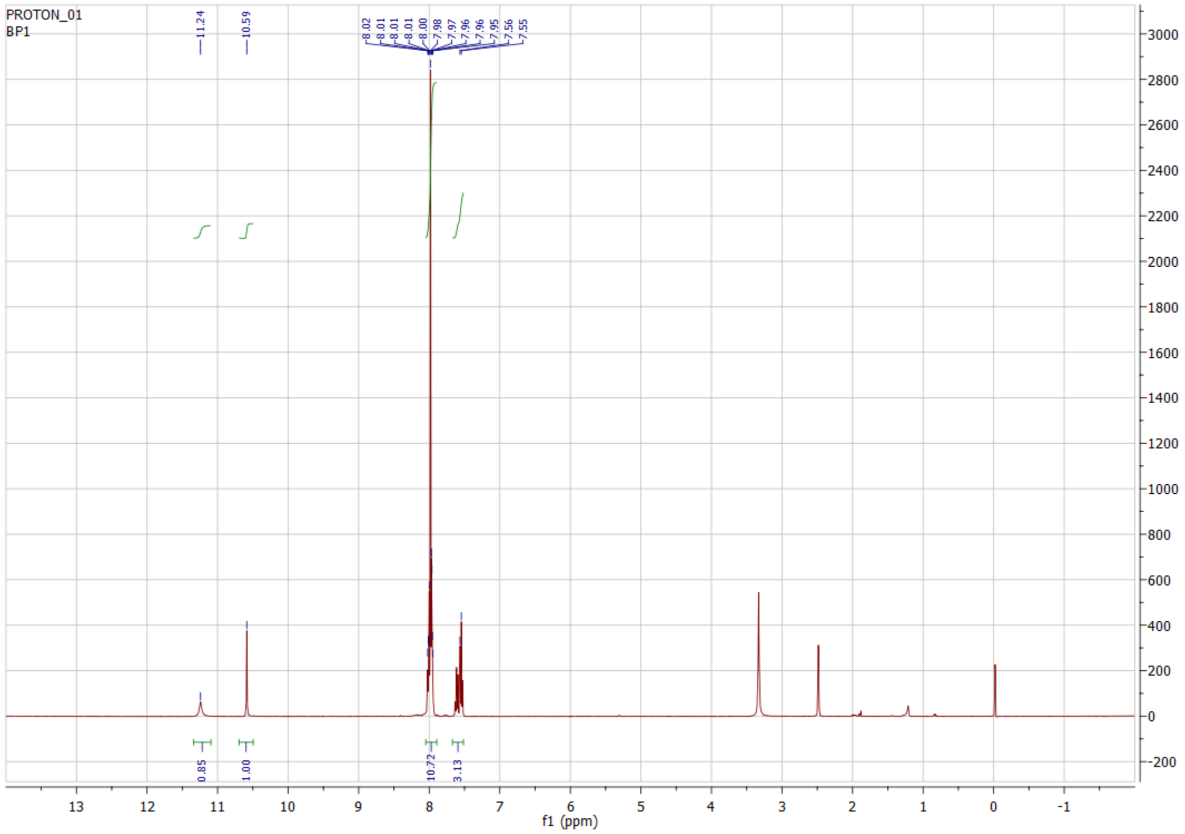

Figure S2. ^{1}H-NMR spectrum for **4a** (400 MHz, DMSO-d6).

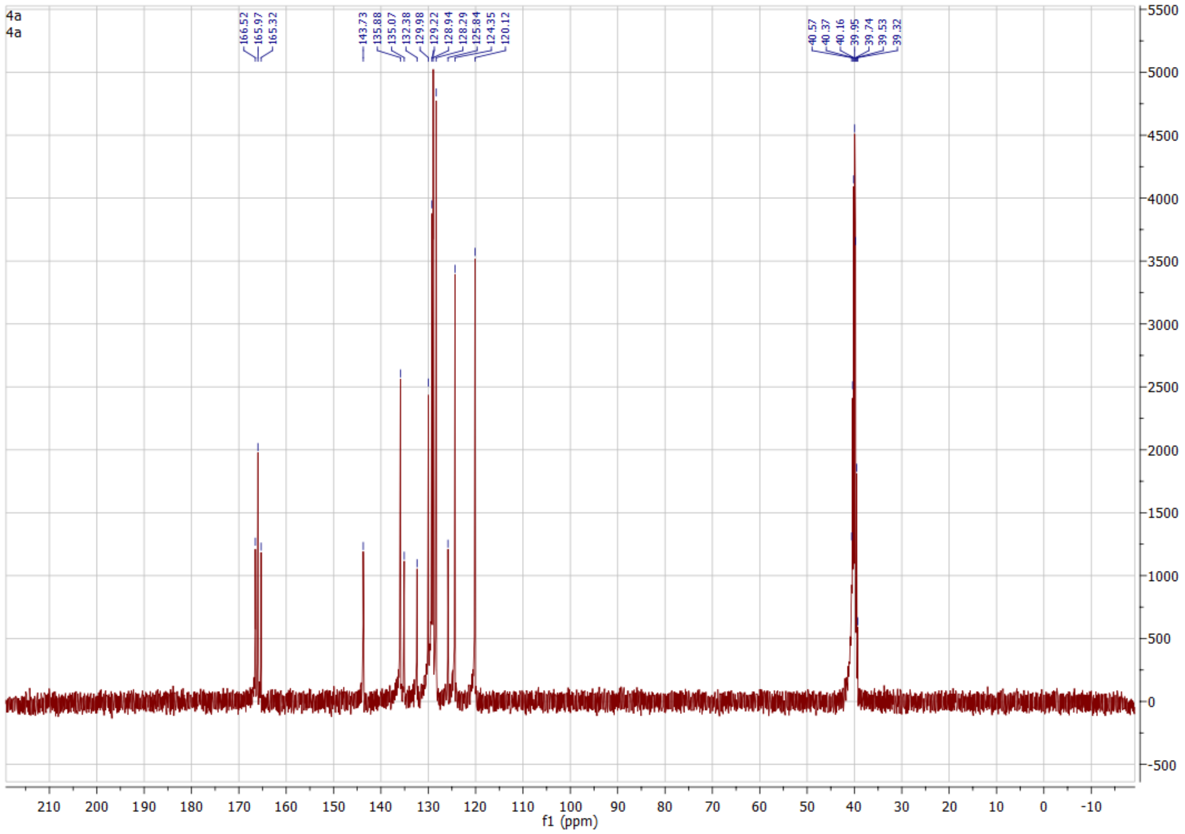

Figure S3. ^{13}C-NMR spectrum for **4a** (100 MHz, DMSO-d6).

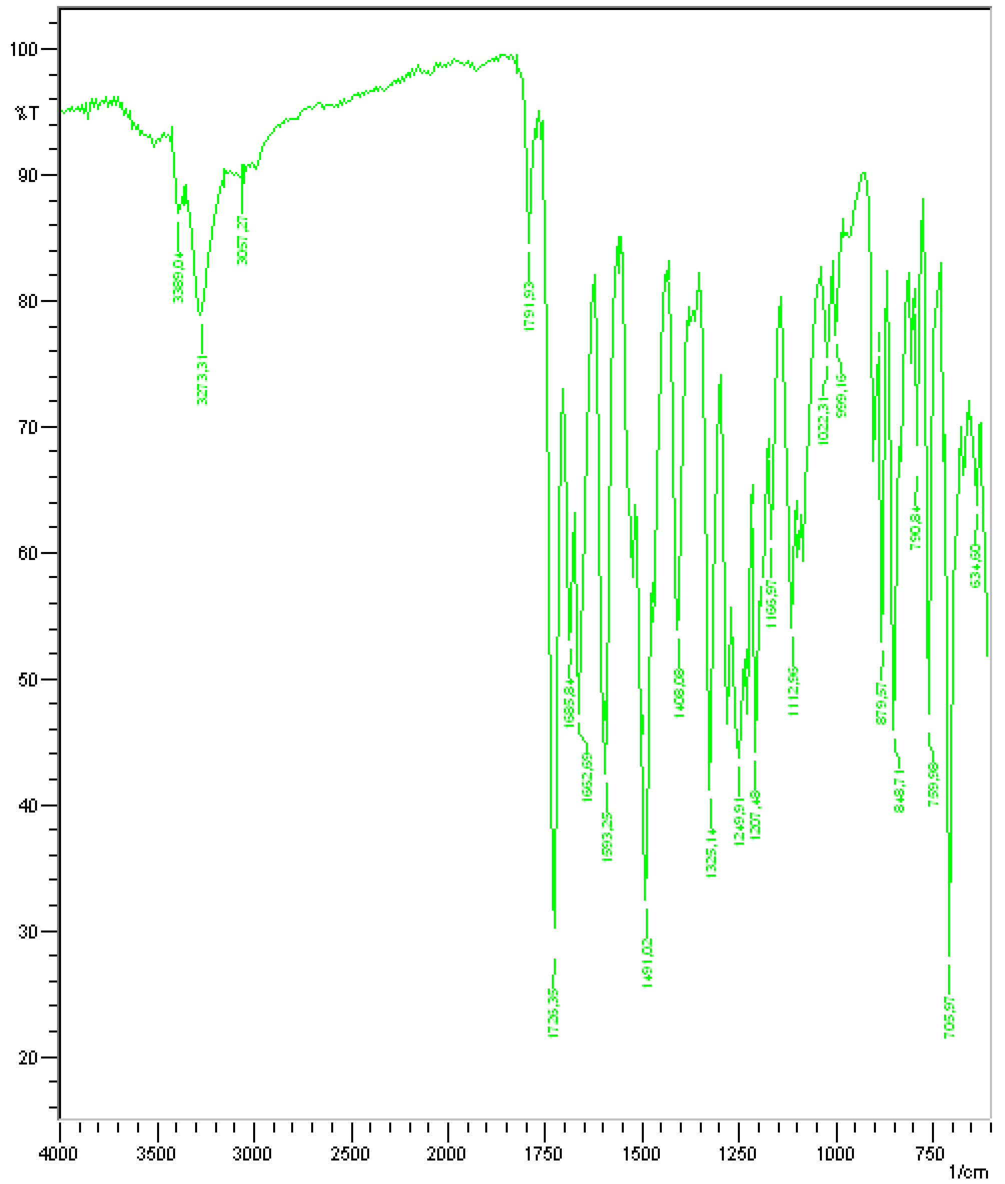

Figure S4. IR spectrum for **4b.**

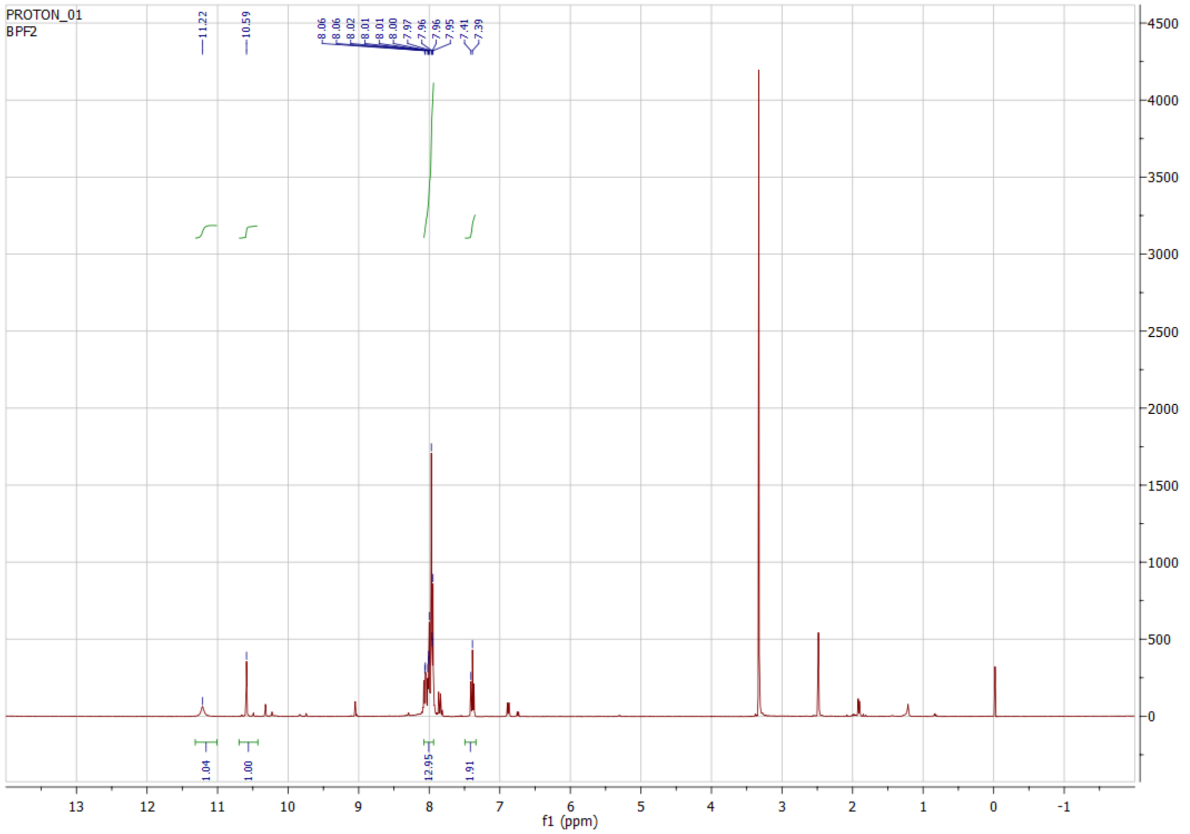

Figure S5. ^{1}H-NMR spectrum for **4b** (400 MHz, DMSO-d$_6$).

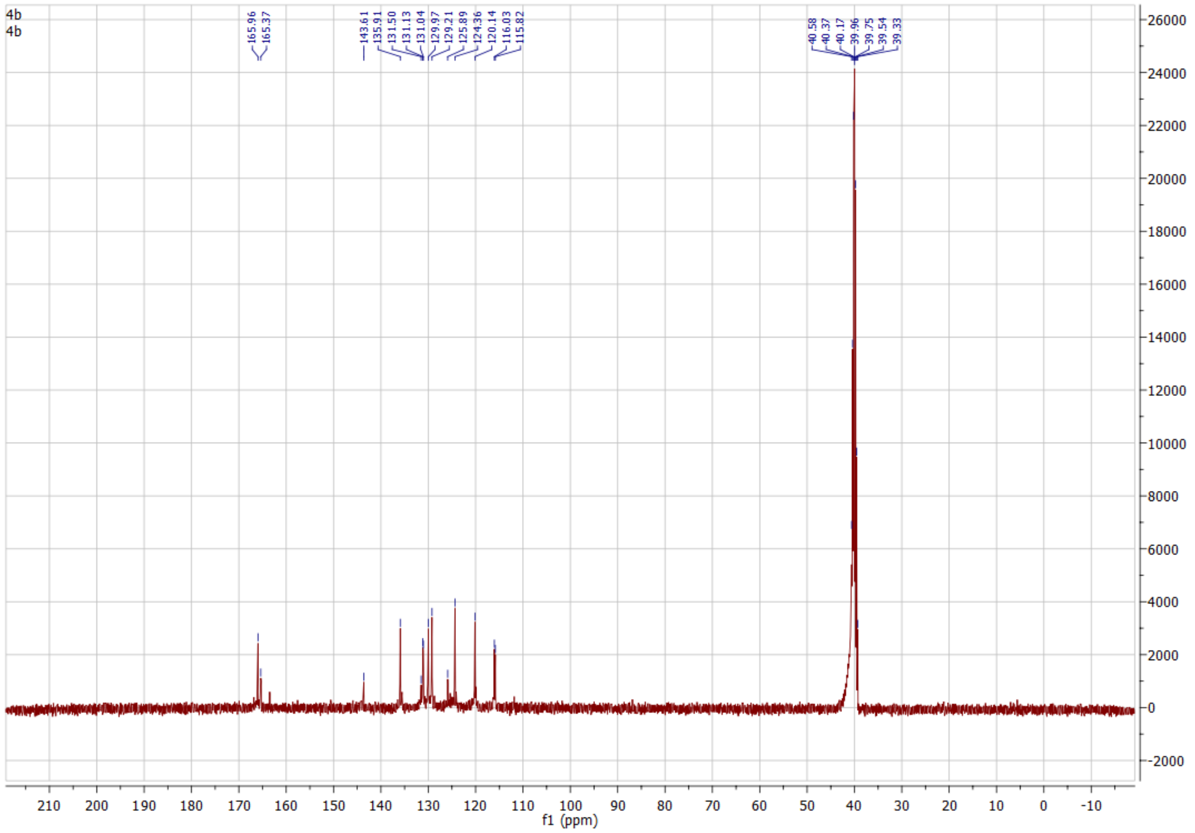

Figure S6. ^{13}C-NMR spectrum for **4b** (100 MHz, DMSO-d$_6$).

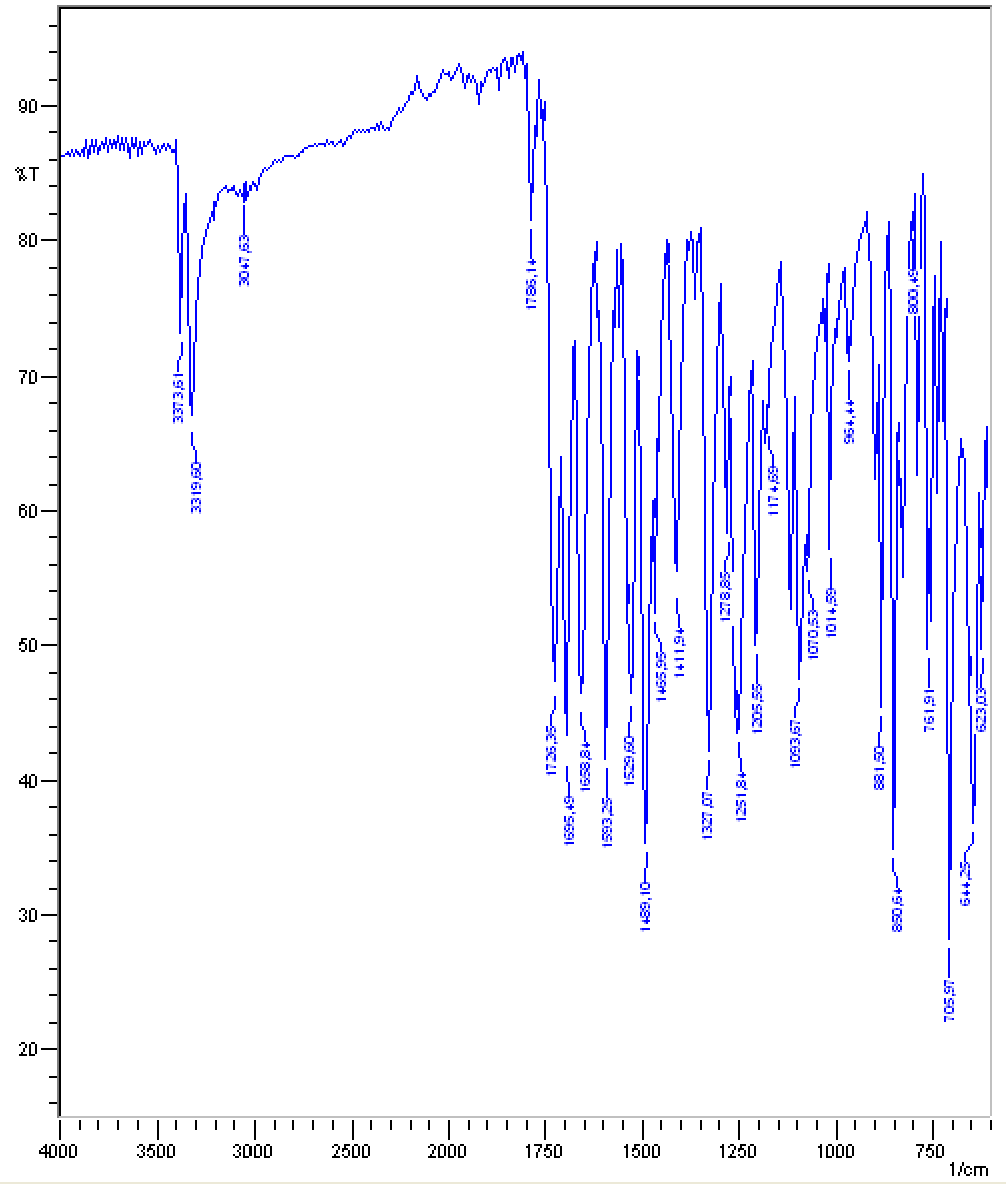

Figure S7. IR spectrum for **4c.**

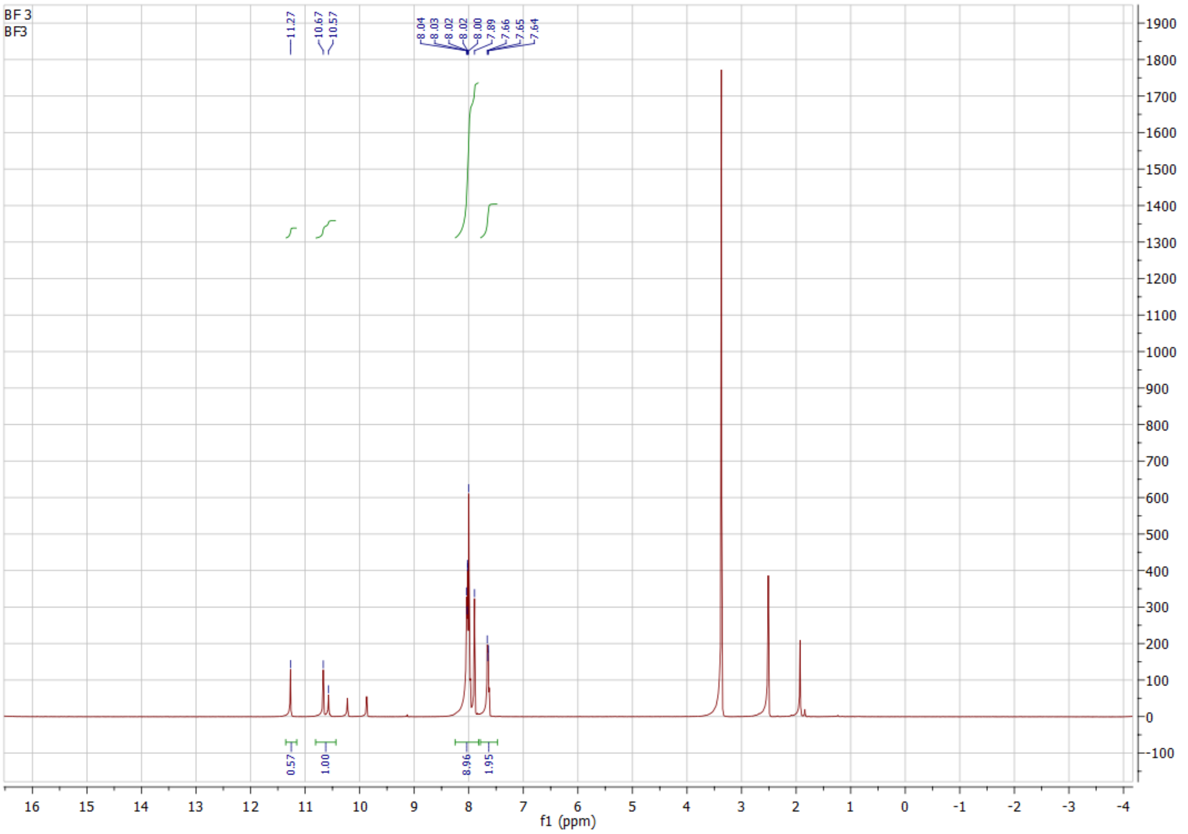

Figure S8. ^{1}H-NMR spectrum for **4c** (400 MHz, DMSO-d6).

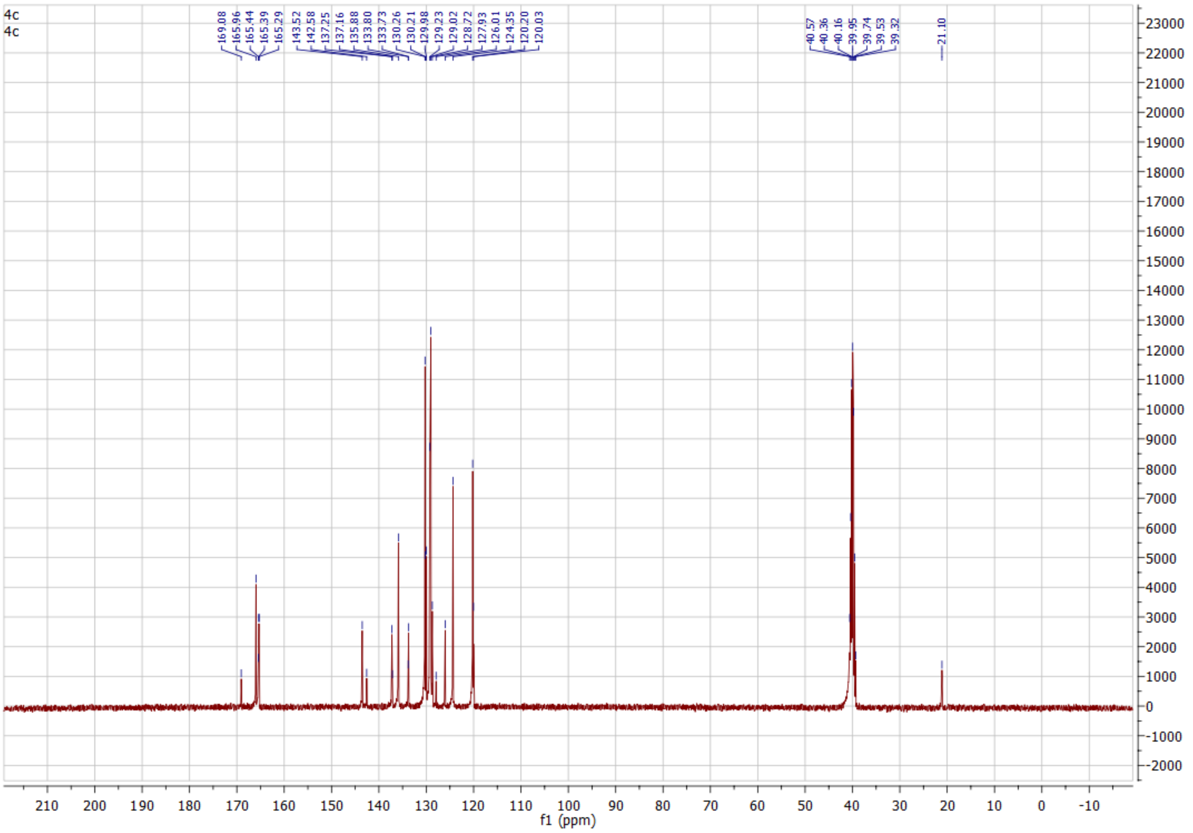

Figure S9. ^{13}C-NMR spectrum for **4c** (100 MHz, DMSO-d6).

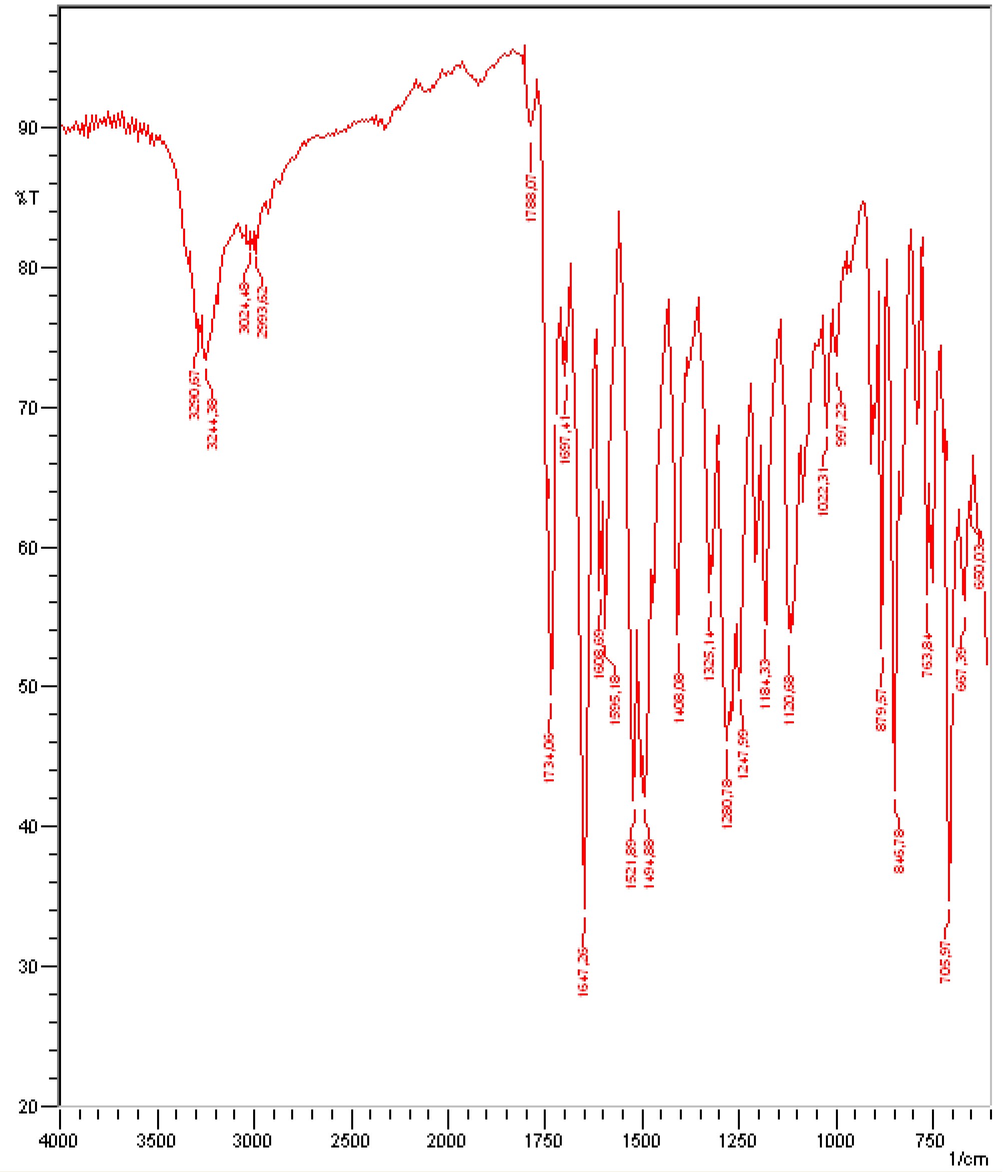

Figure S10. IR spectrum for **4d.**

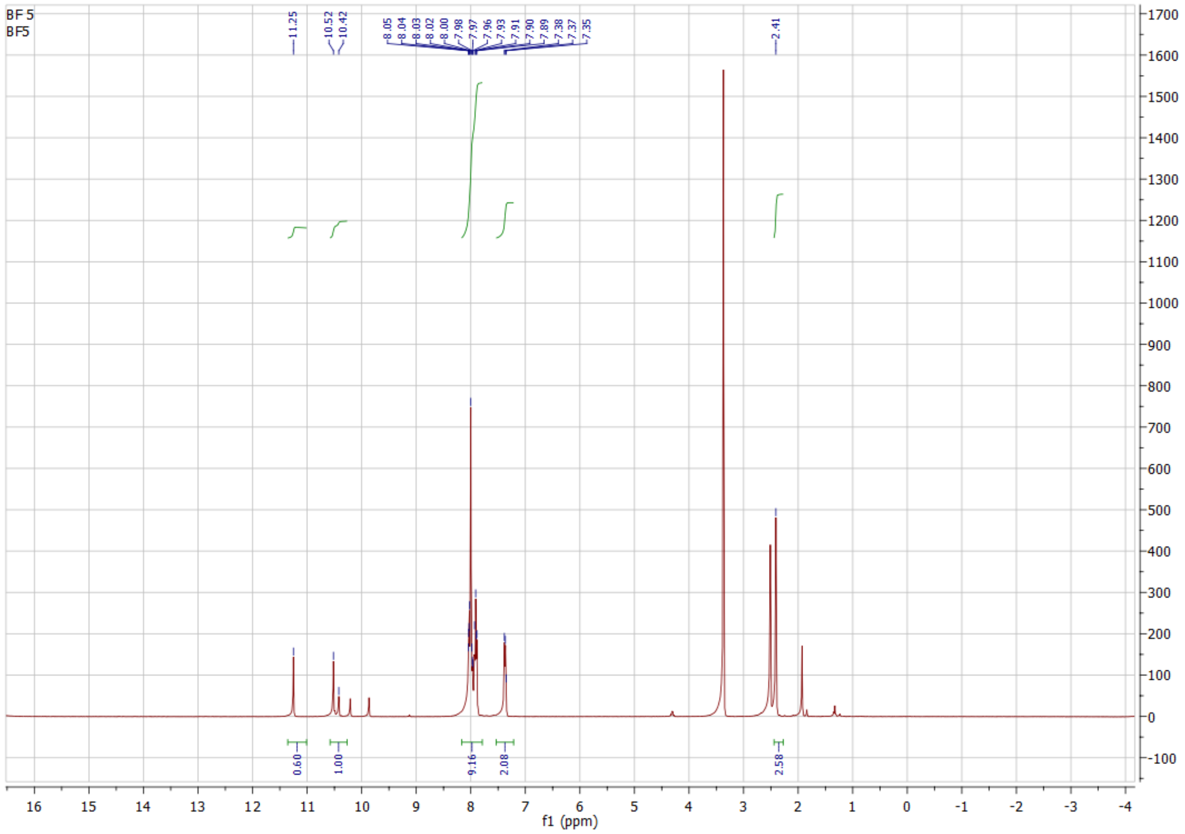

Figure S11. ^{1}H-NMR spectrum for **4d** (400 MHz, DMSO-d_6).

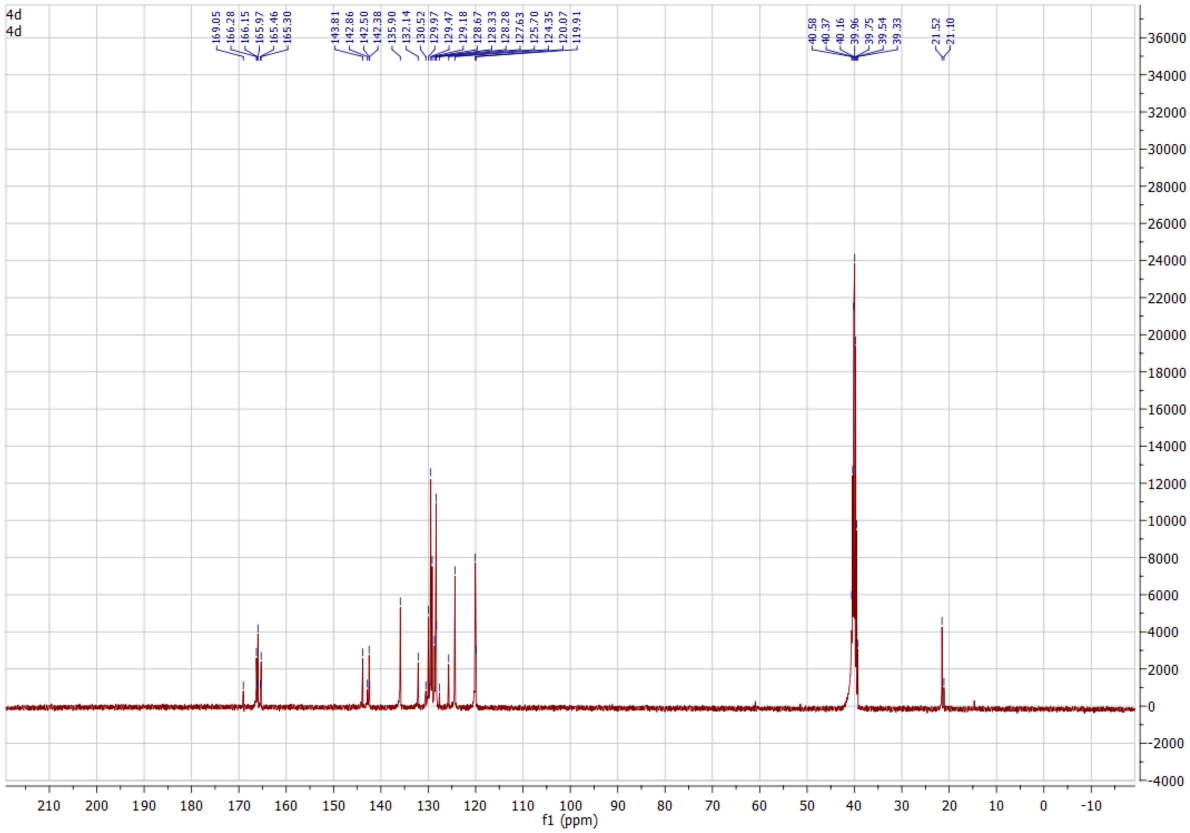

Figure S12. ^{13}C-NMR spectrum for **4d** (100 MHz, DMSO-d_6).

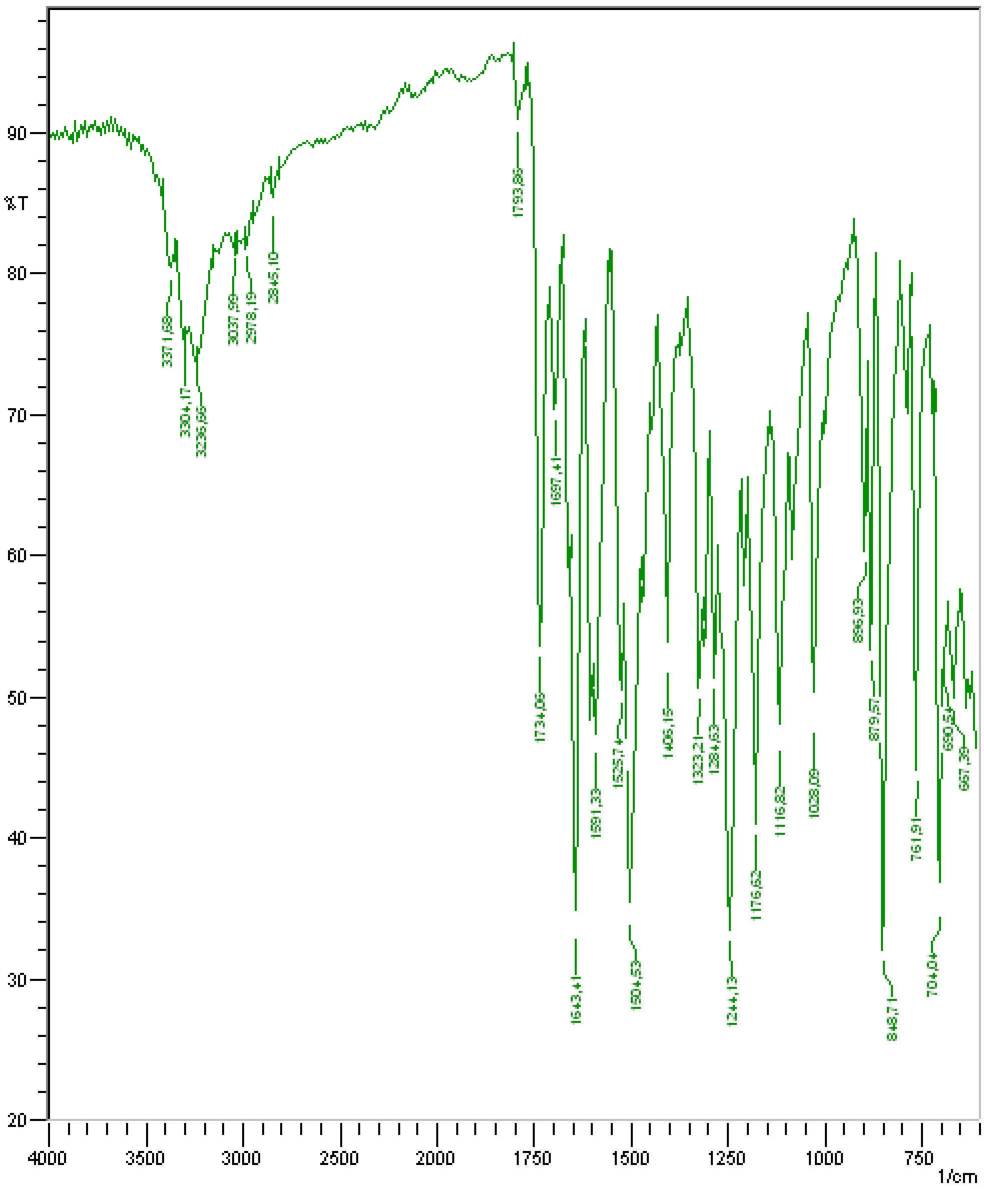

Figure S13. IR spectrum for **4e.**

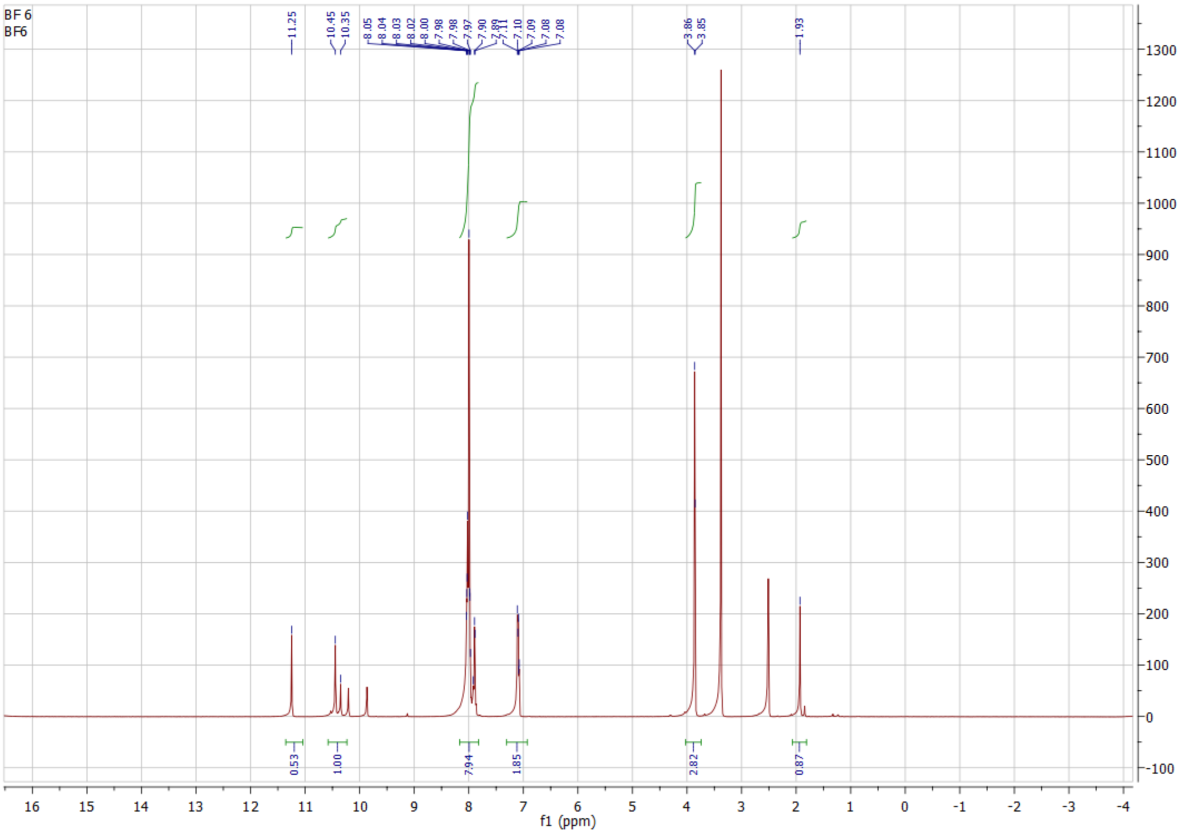

Figure S14. ^{1}H-NMR spectrum for **4e** (400 MHz, DMSO-d_6).

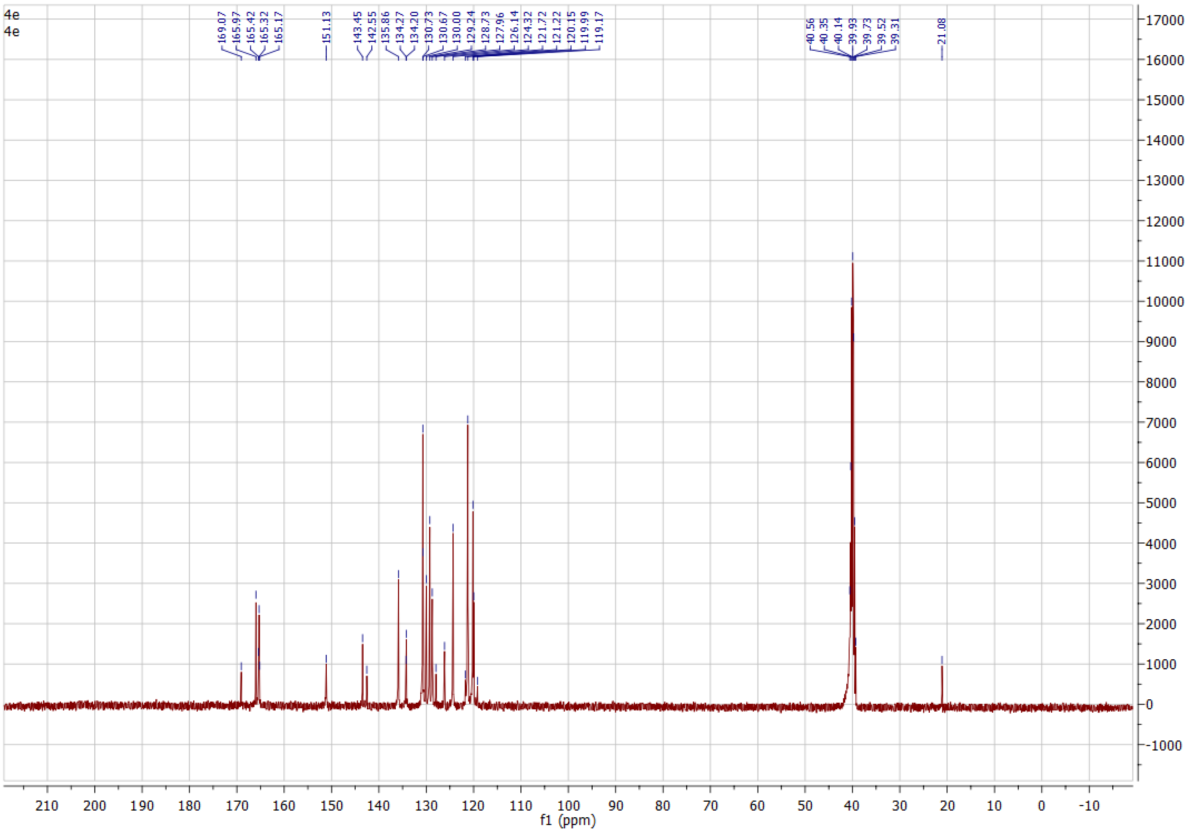

Figure S15. ^{13}C-NMR spectrum for **4e** (100 MHz, DMSO-d_6).

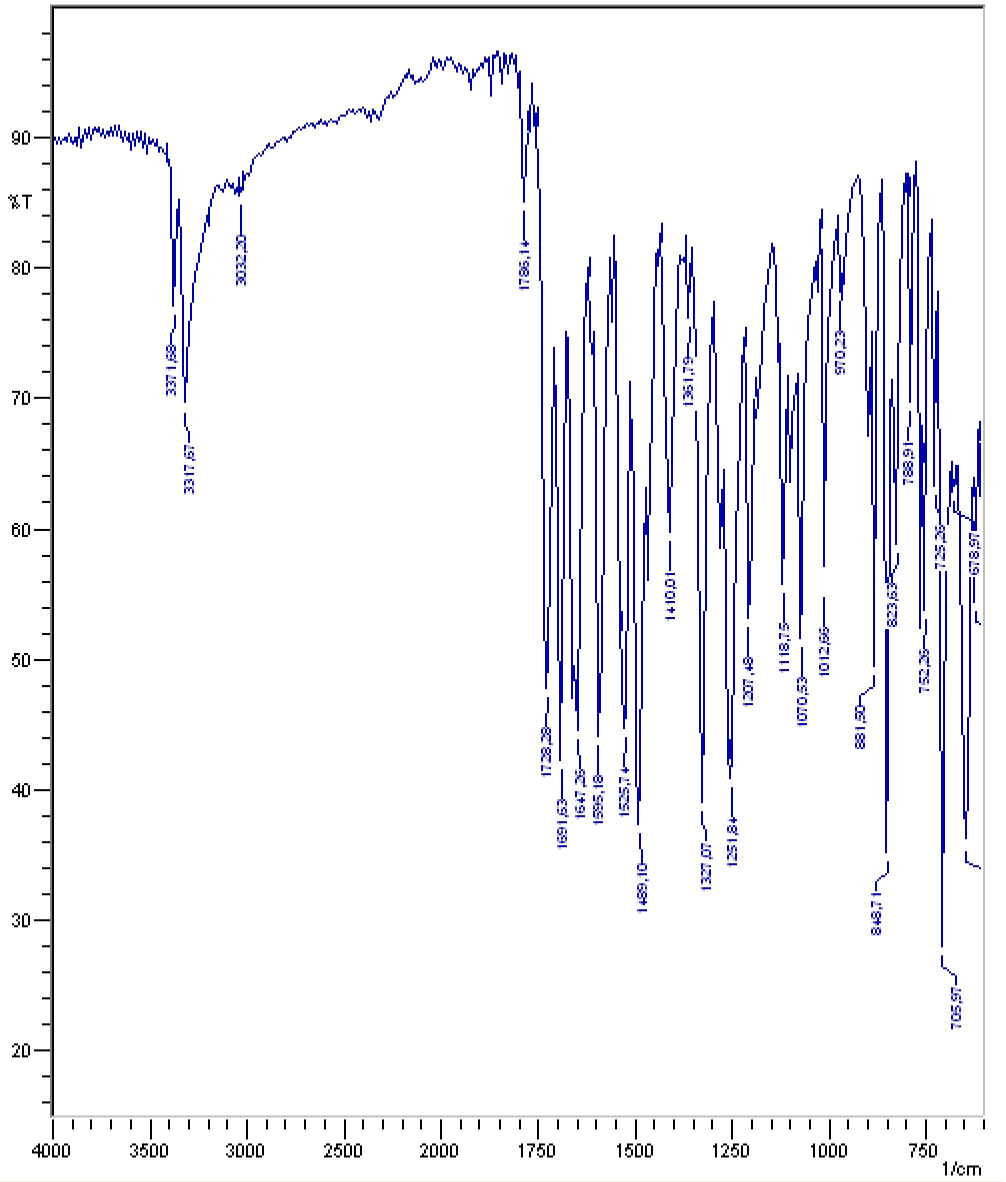

Figure S16. IR spectrum for **4f.**

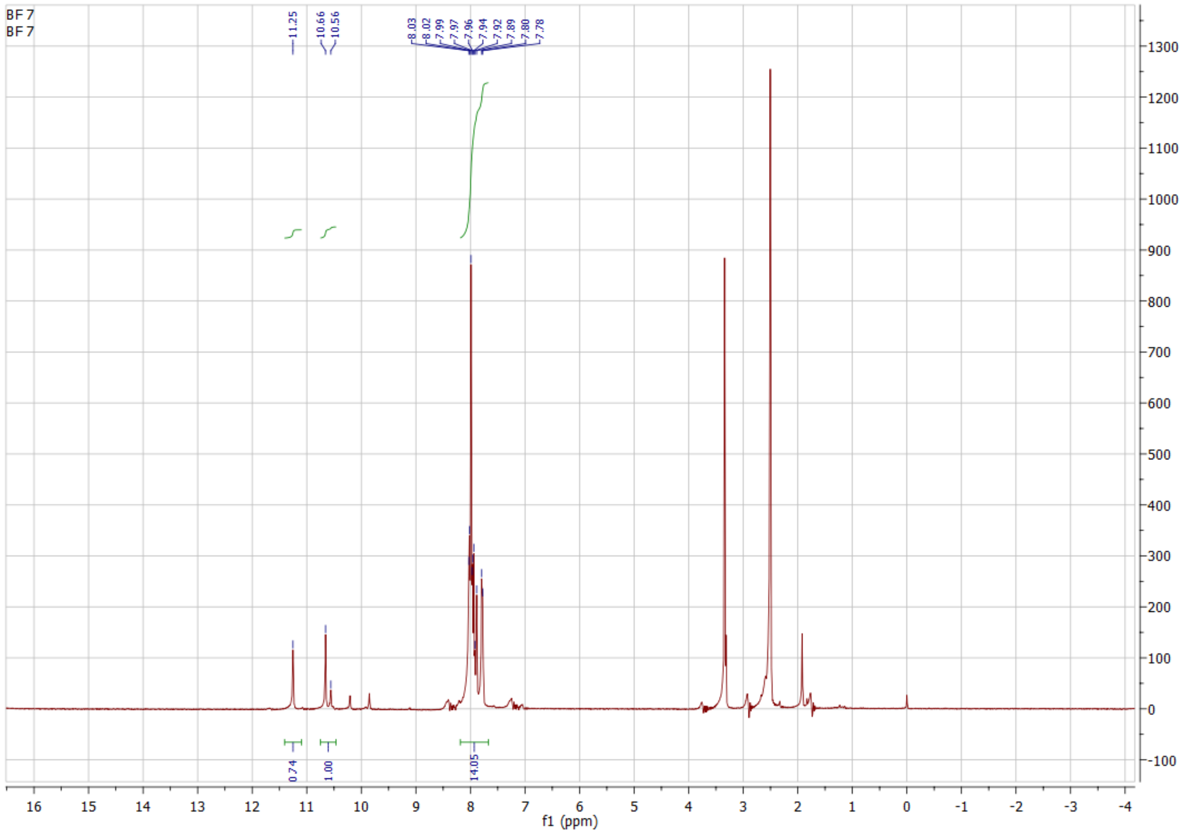

Figure S17. ^{1}H-NMR spectrum for **4f** (400 MHz, DMSO-d_6).

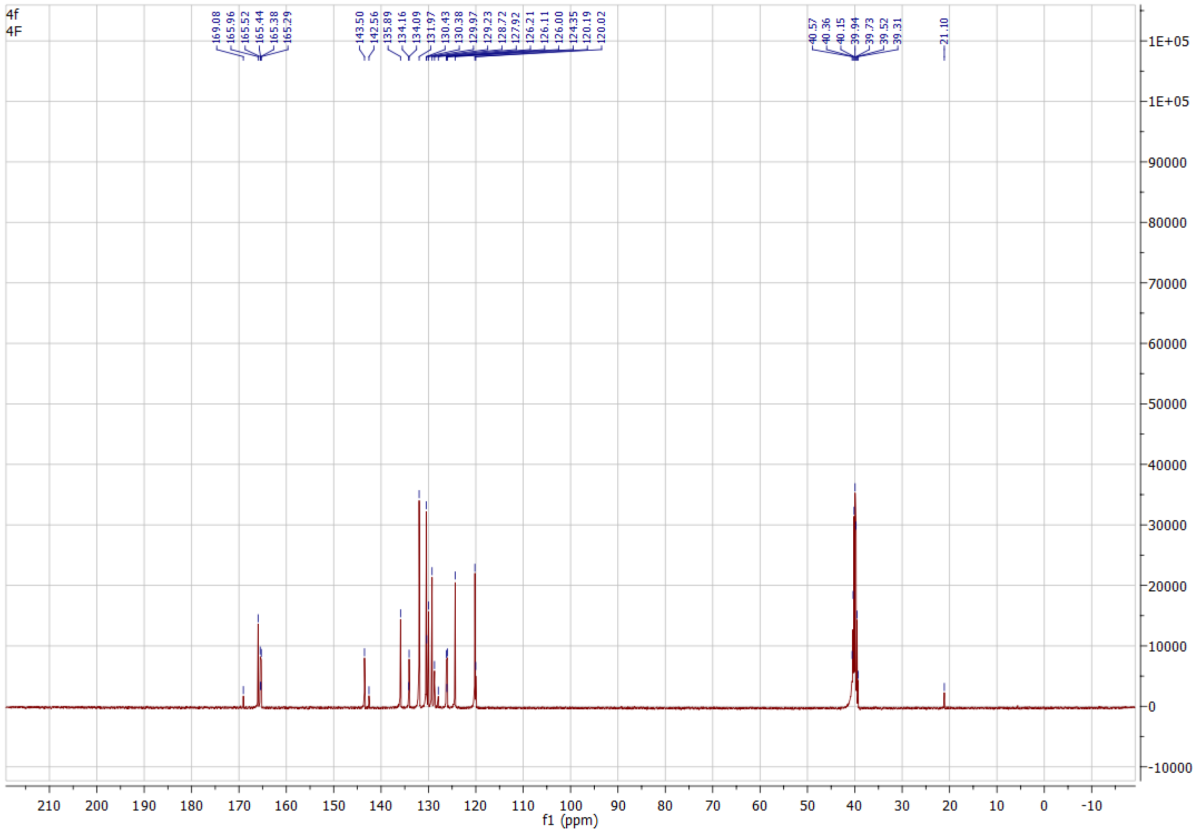

Figure S18. ^{13}C-NMR spectrum for **4f** (100 MHz, DMSO-d_6).

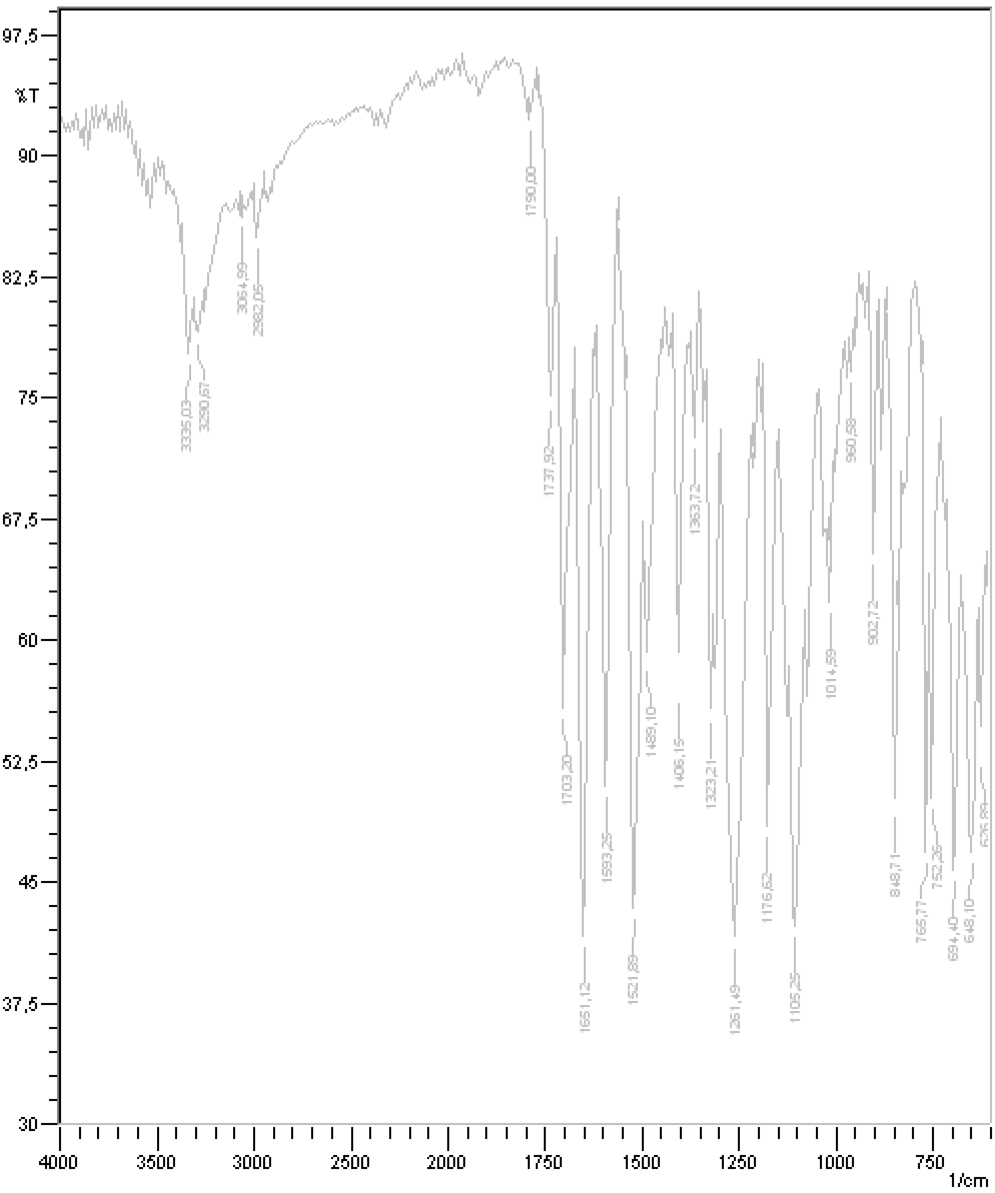

Figure S19. IR spectrum for **4g.**

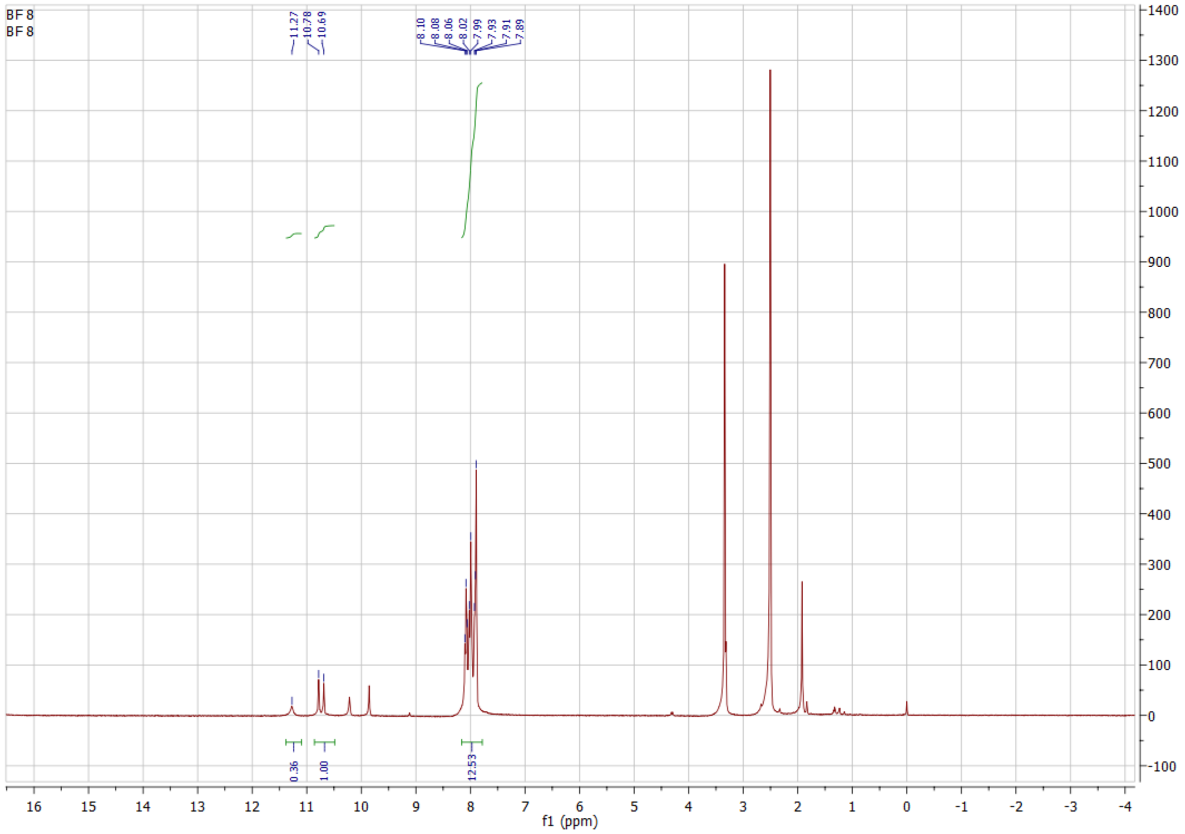

Figure S20. ^{1}H-NMR spectrum for **4g** (400 MHz, DMSO-d6).

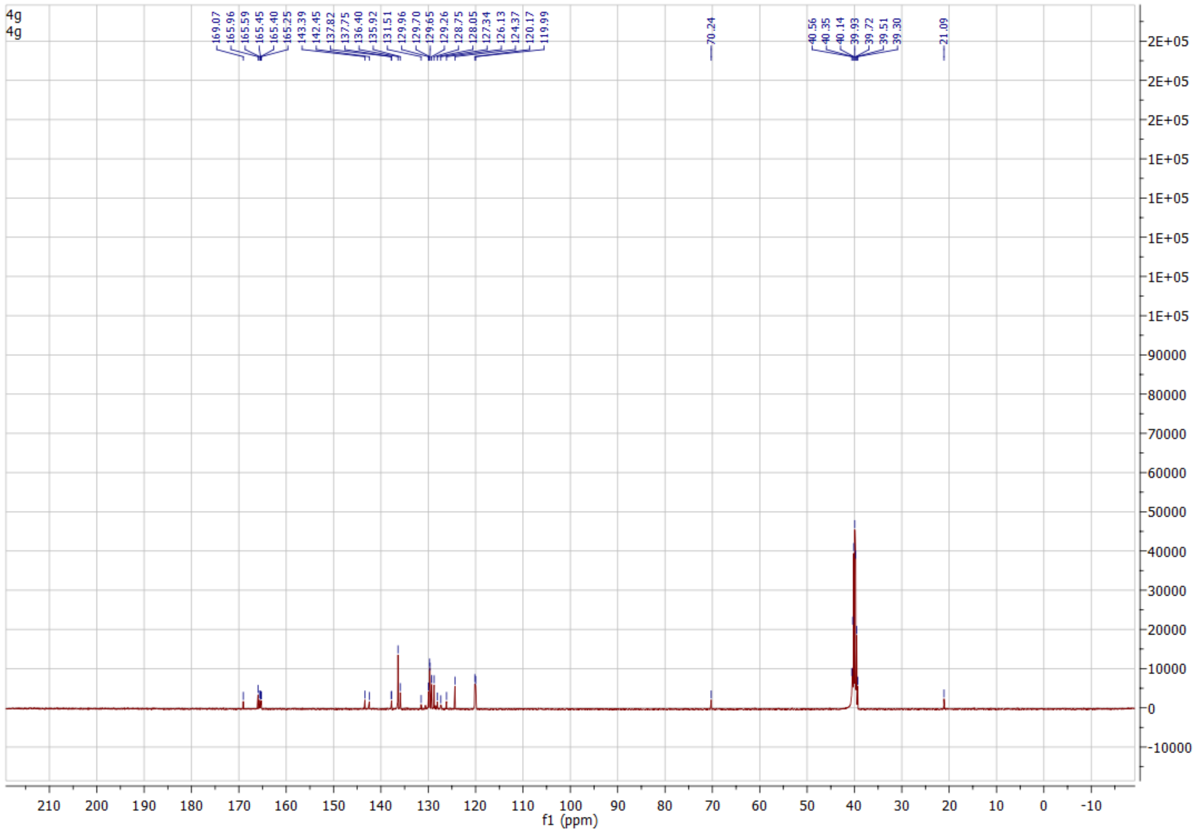

Figure S21. ^{13}C-NMR spectrum for **4g** (100 MHz, DMSO-d6).

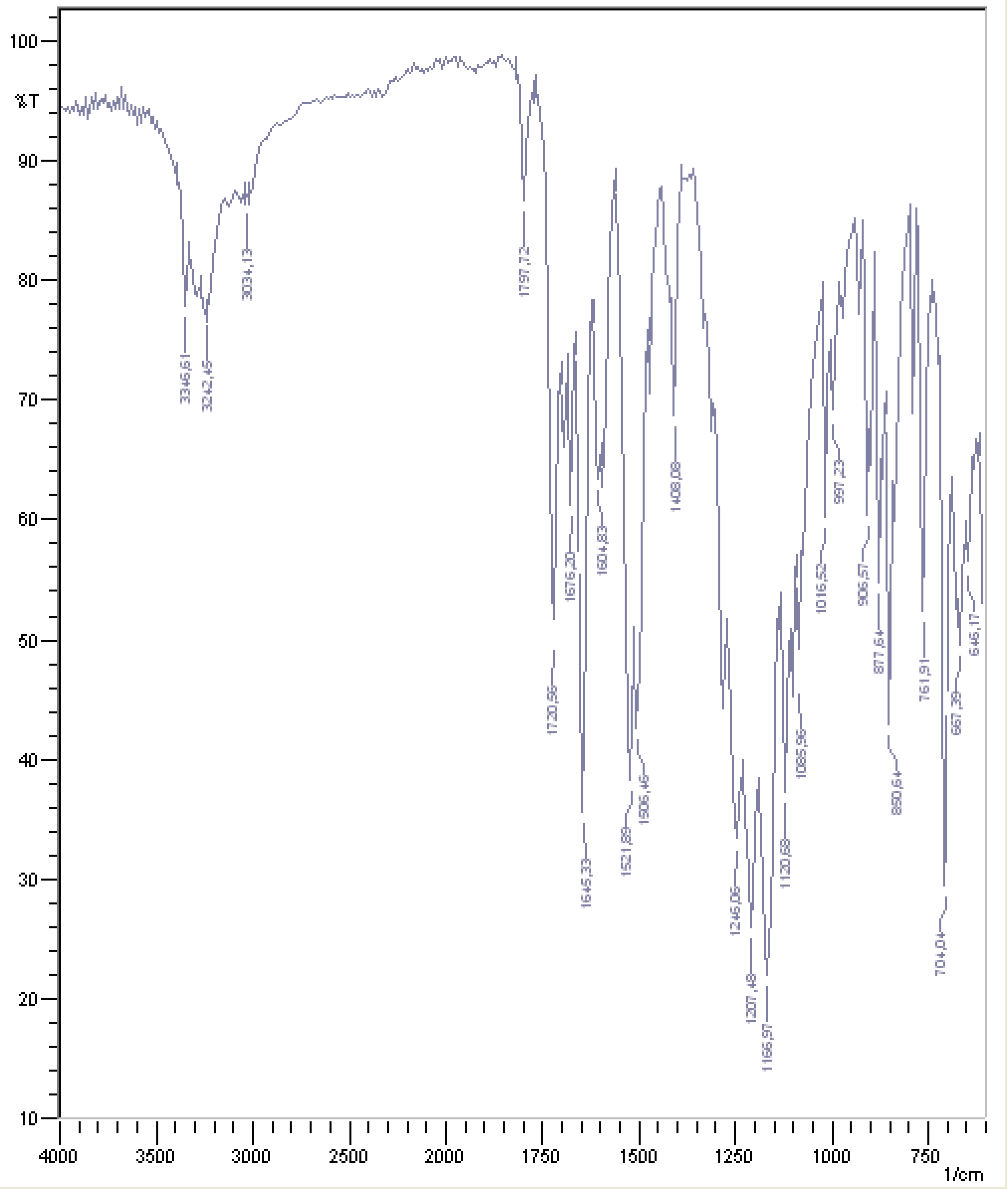

Figure S22. IR spectrum for **4h.**

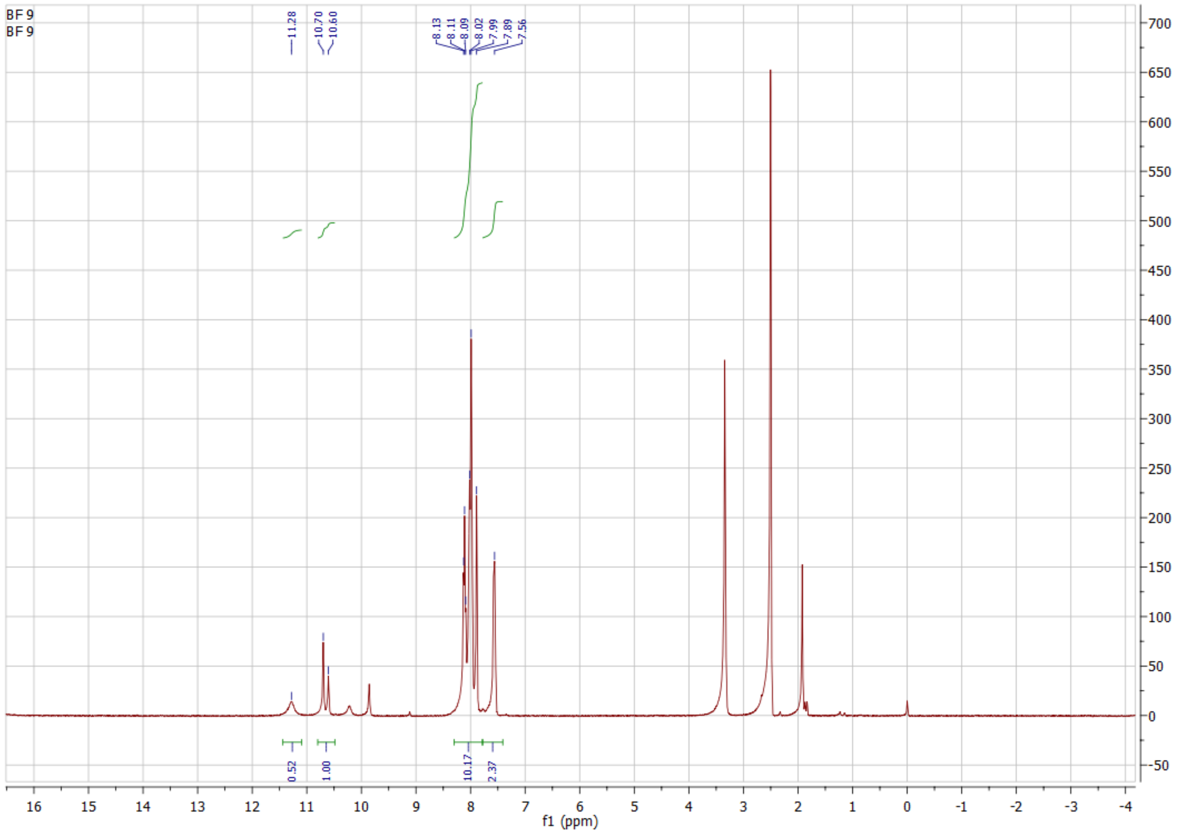

Figure S23. ^{1}H-NMR spectrum for **4h** (400 MHz, DMSO-d$_6$).

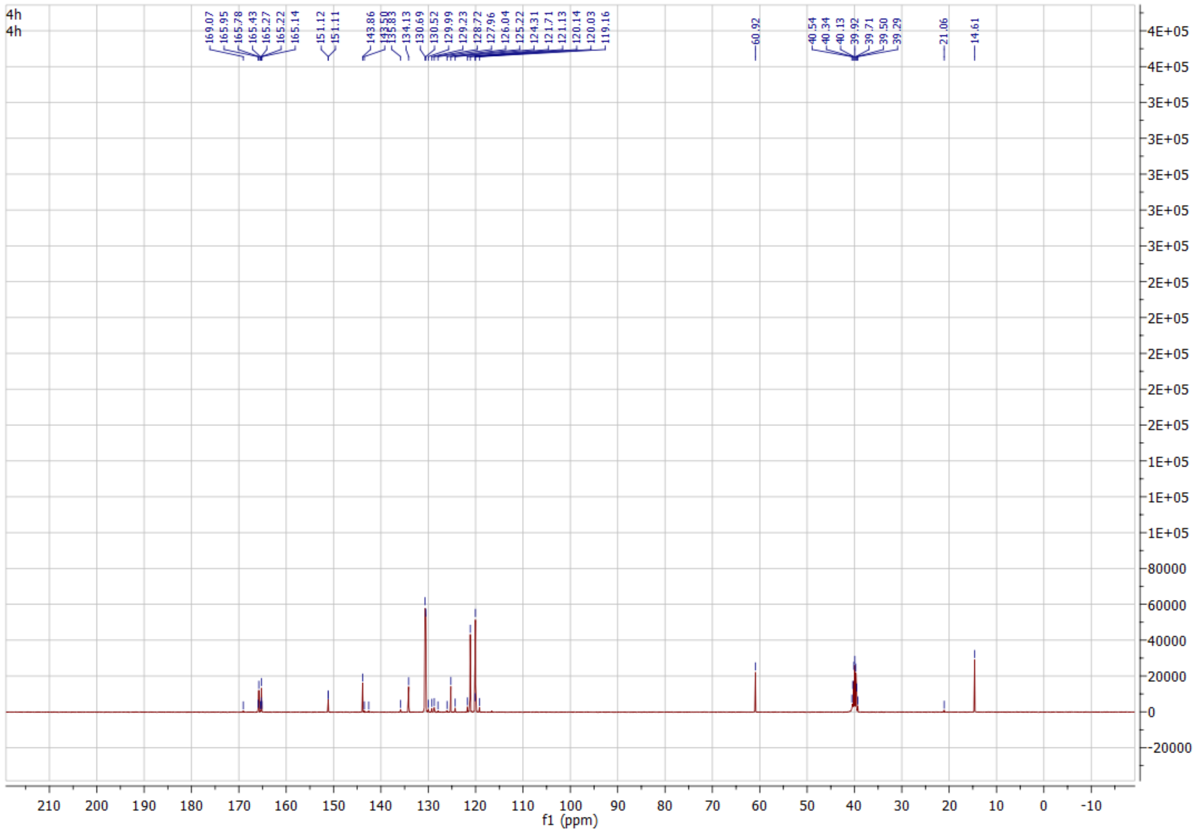

Figure S24. ^{13}C-NMR spectrum for **4h** (100 MHz, DMSO-d$_6$).

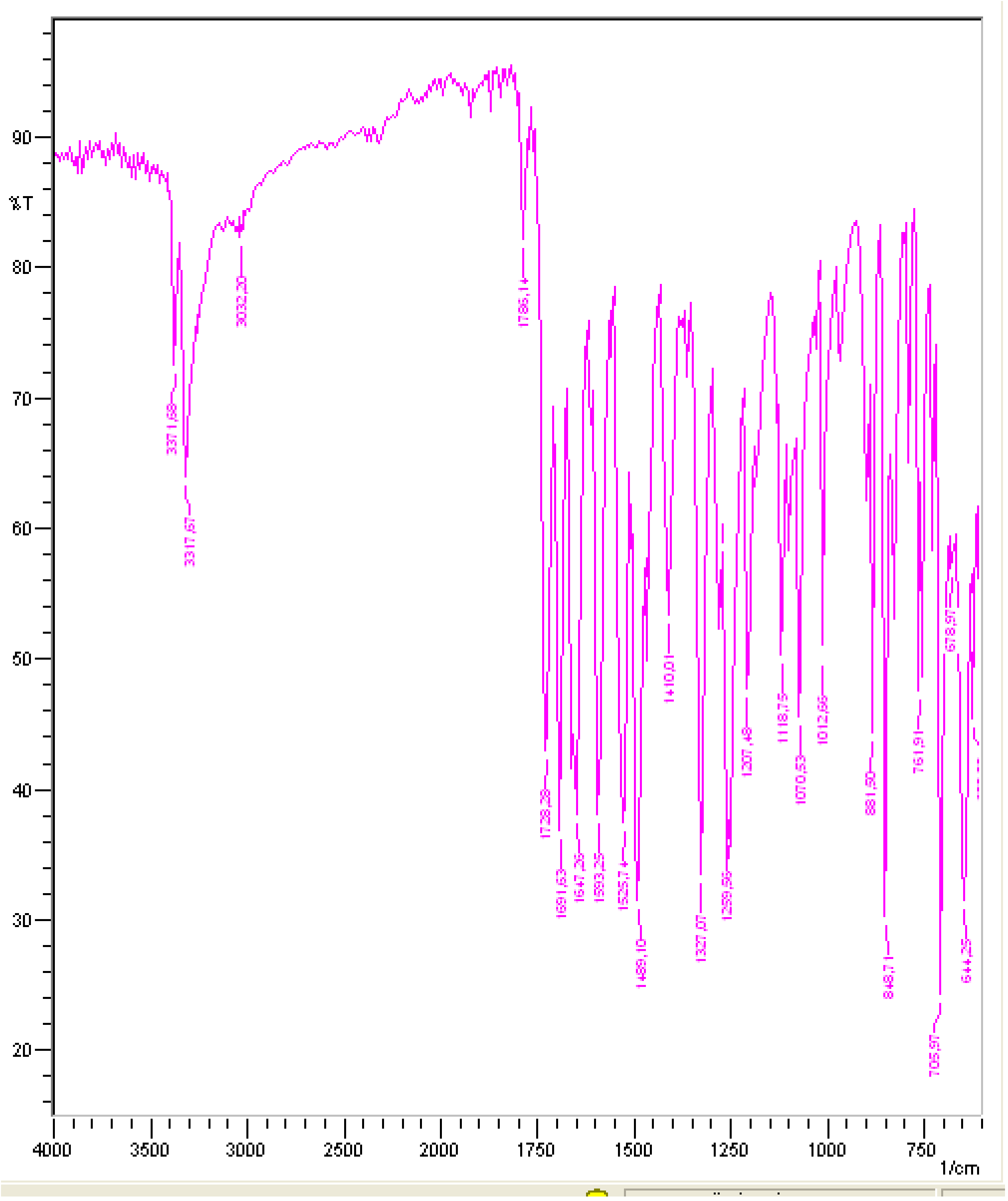

Figure S25. IR spectrum for **4i.**

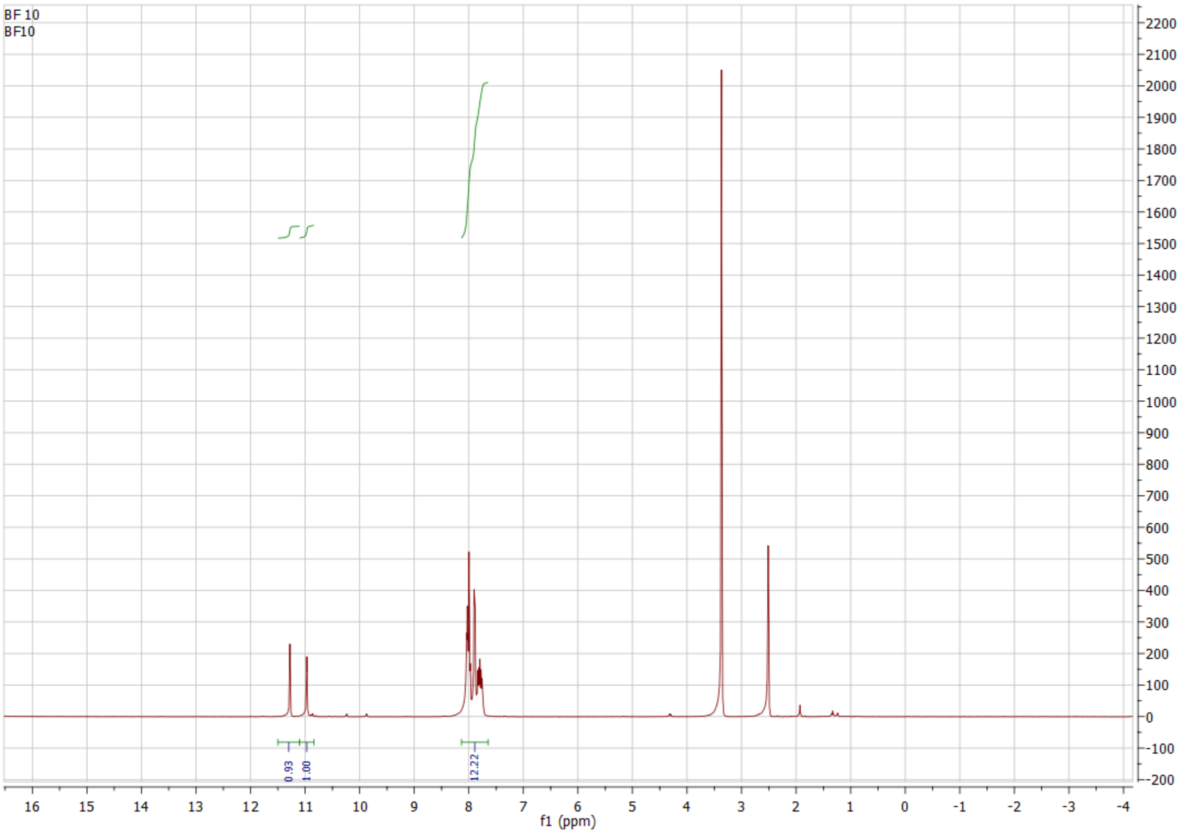

Figure S26. ^{1}H-NMR spectrum for **4i** (400 MHz, DMSO-d_6).

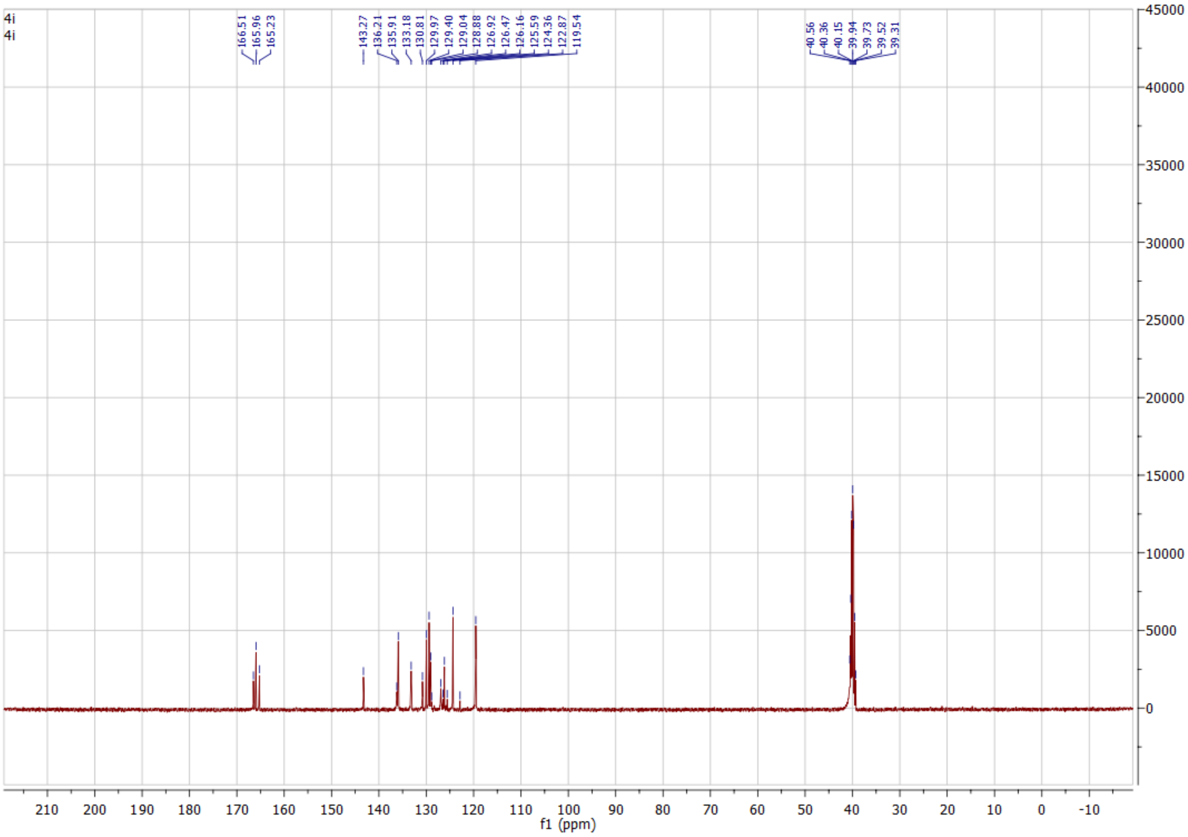

Figure S27. ^{13}C-NMR spectrum for **4i** (100 MHz, DMSO-d_6).

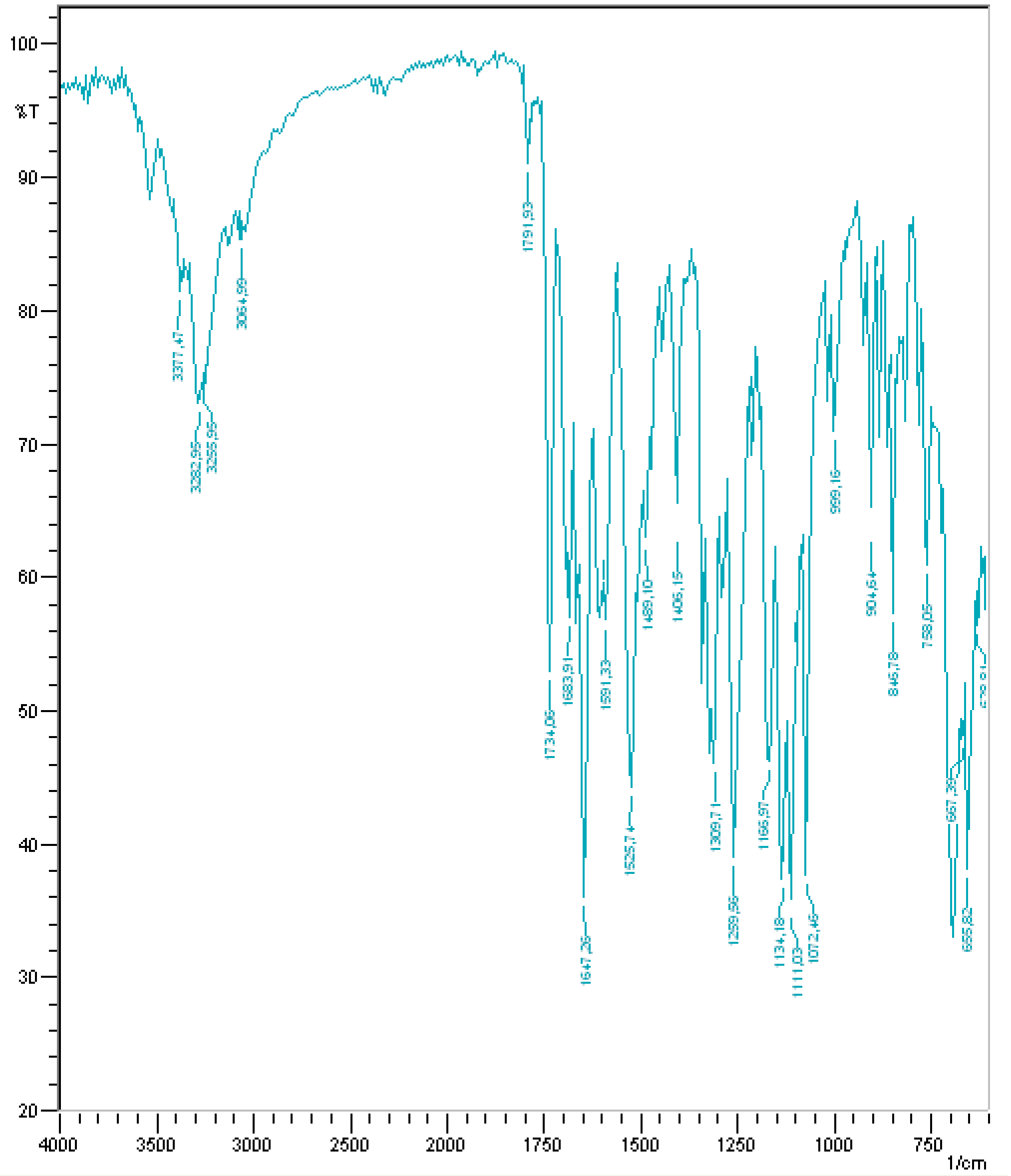

Figure S28. IR spectrum for **4j.**

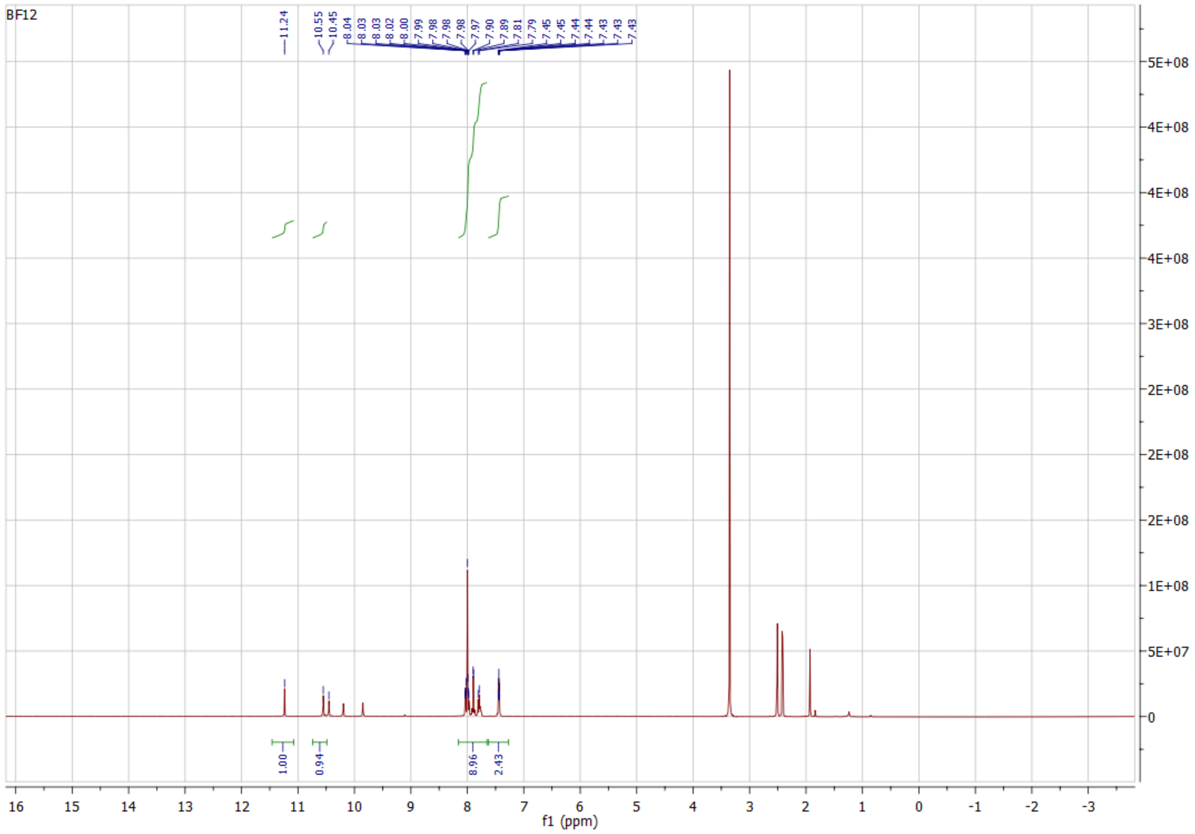

Figure S29. ^{1}H-NMR spectrum for **4j** (400 MHz, DMSO-d_6).

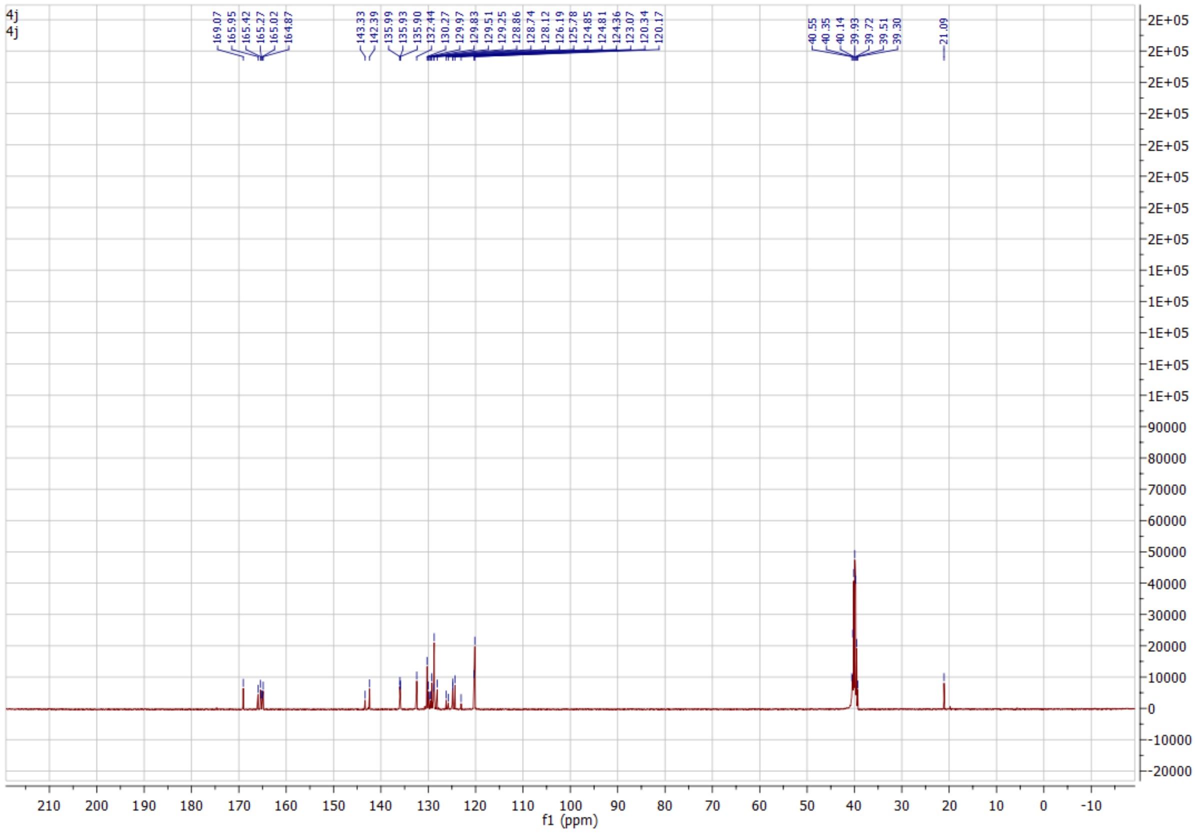

Figure S30. ^{13}C-NMR spectrum for **4j** (100 MHz, DMSO-d_6).

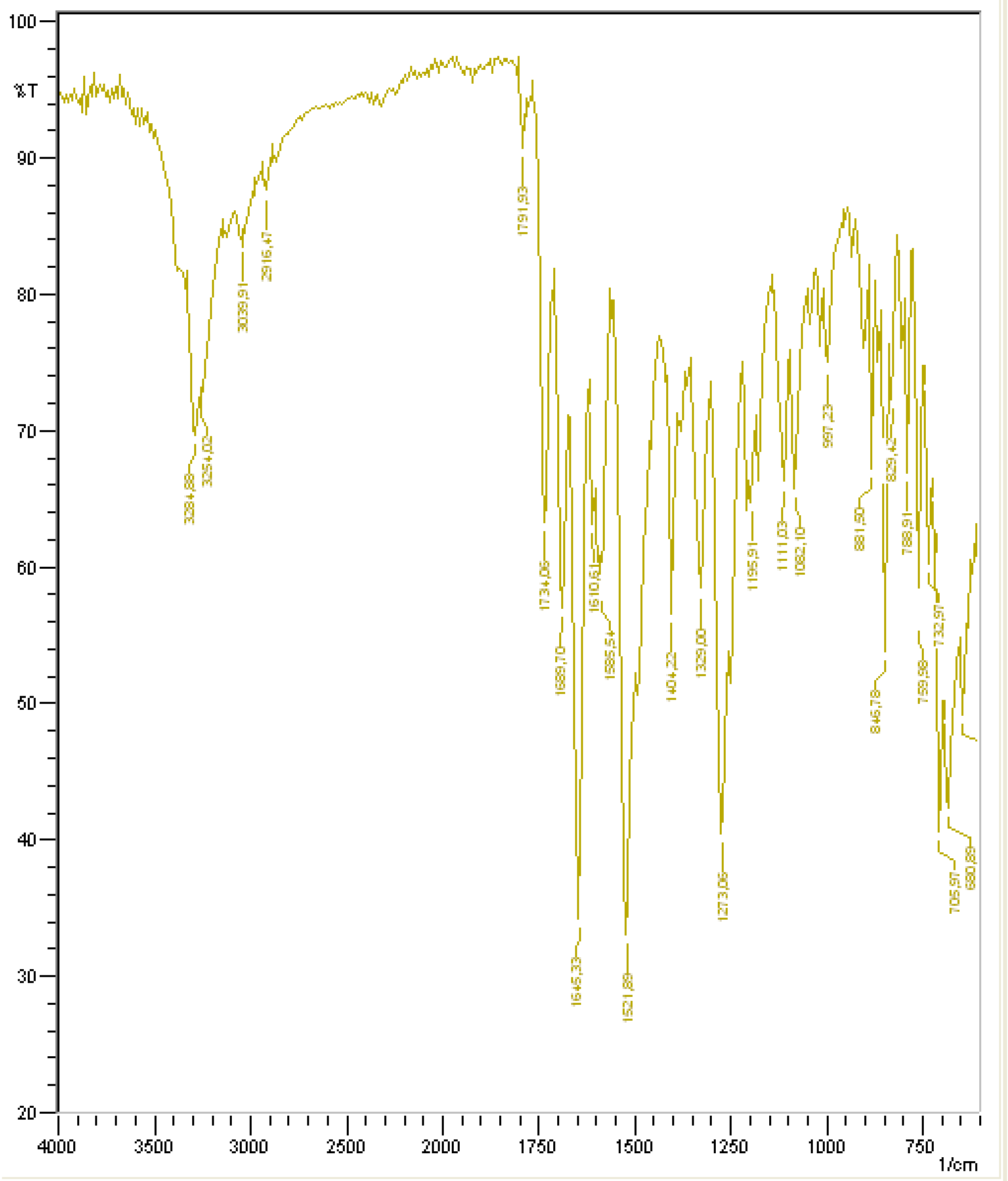

Figure S31. IR spectrum for **4k.**

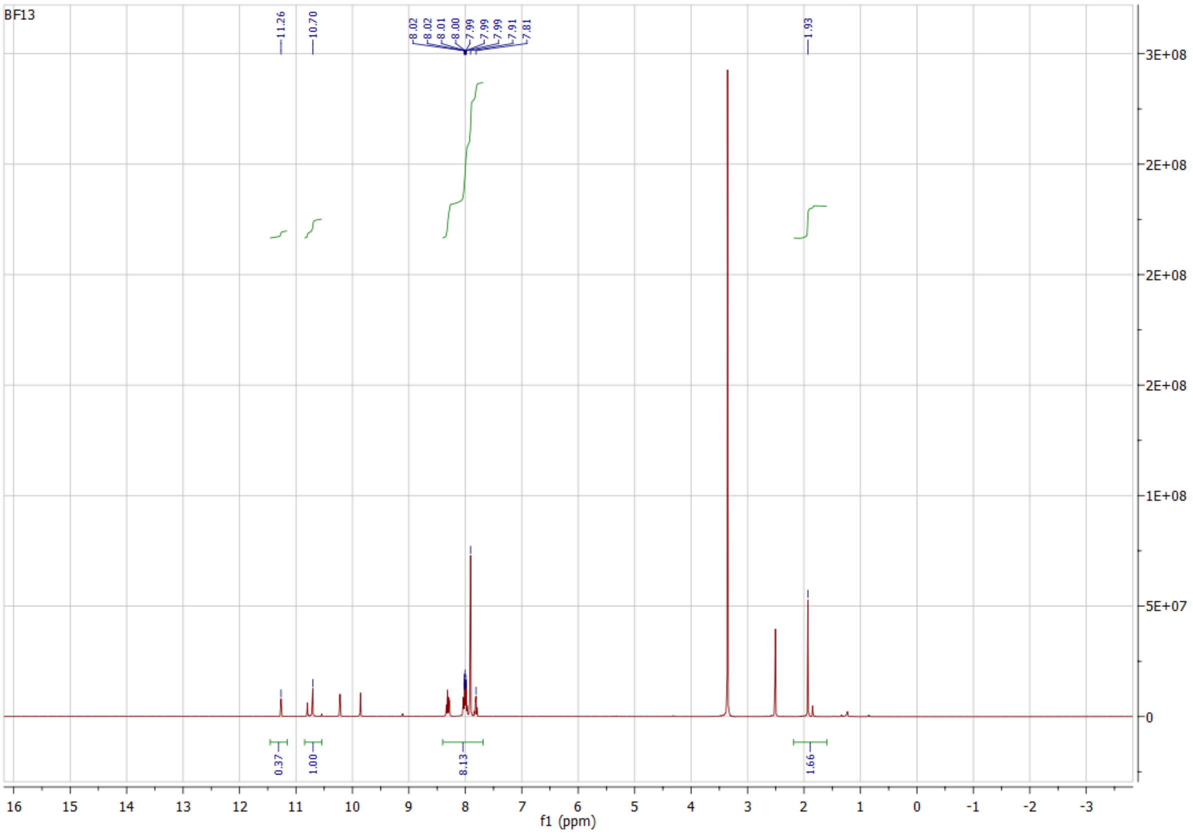

Figure S32. ^{1}H-NMR spectrum for **4k** (400 MHz, DMSO-d_6).

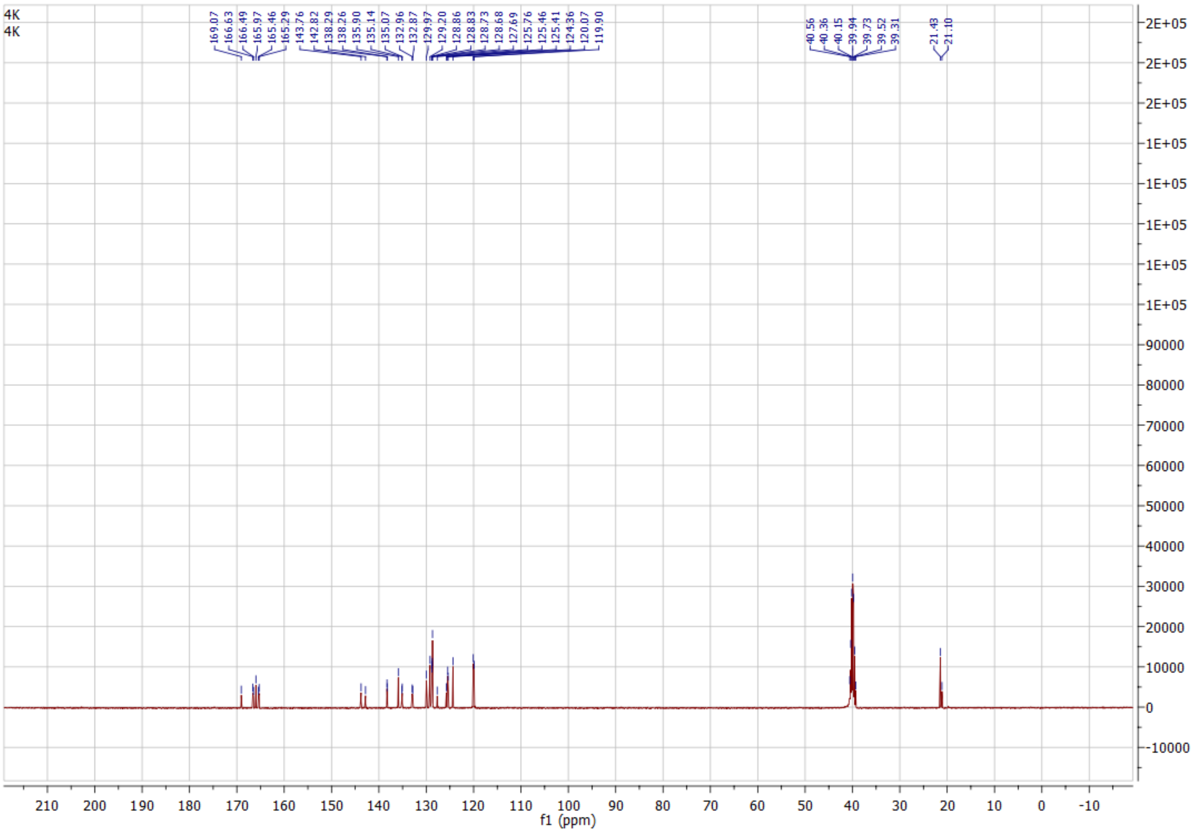

Figure S33. ^{13}C-NMR spectrum for **4k** (100 MHz, DMSO-d_6).

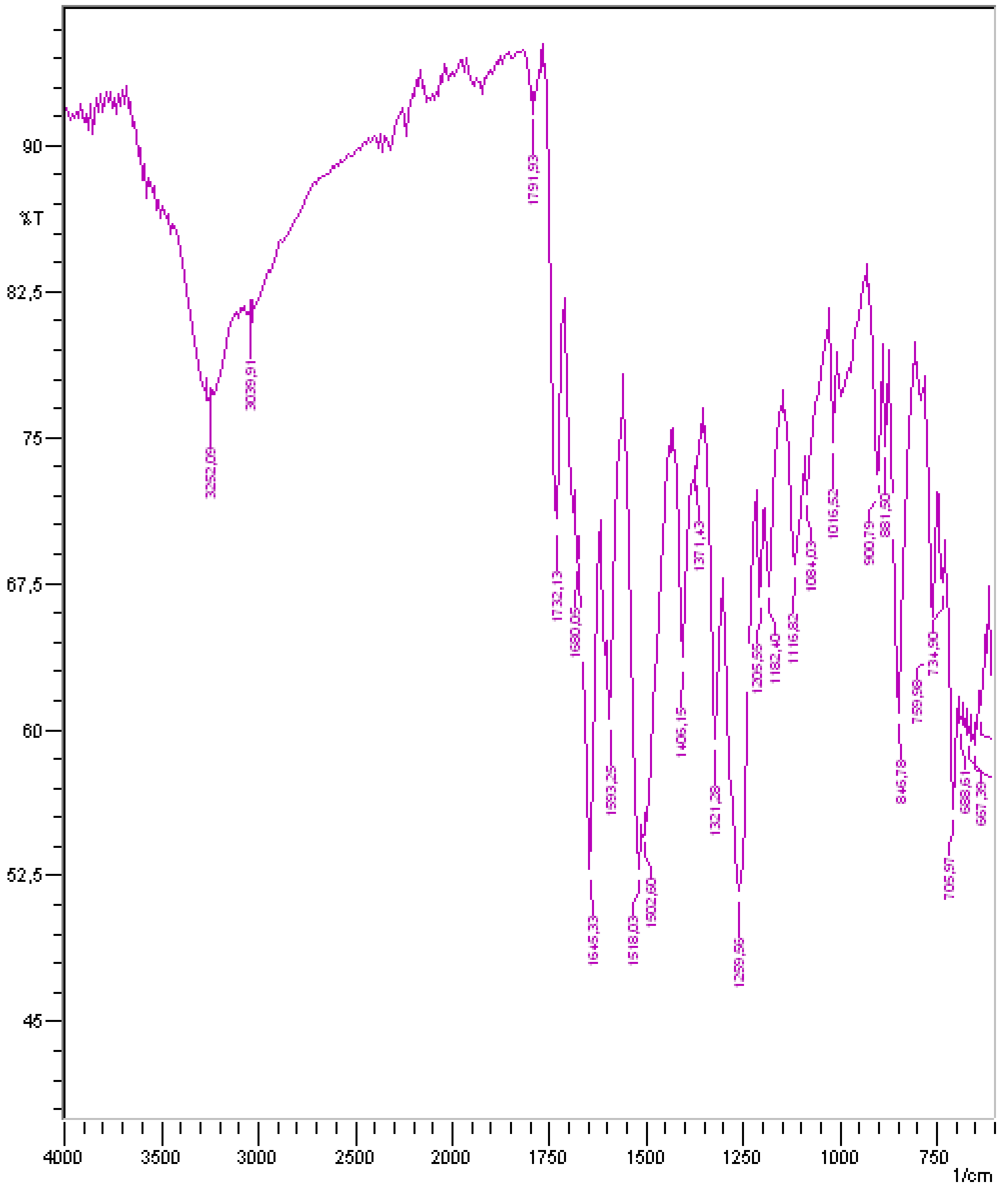

Figure S34. IR spectrum for **4l.**

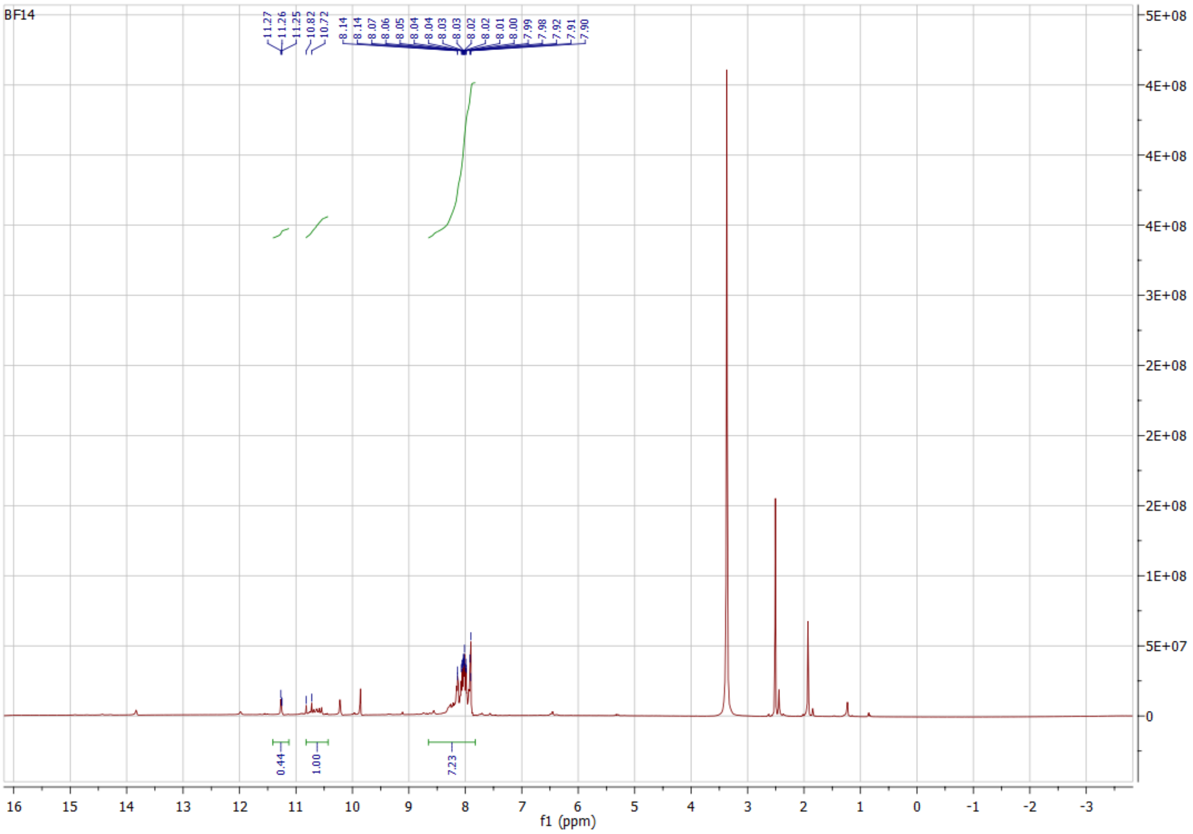

Figure S35. ^{1}H-NMR spectrum for **4l** (400 MHz, DMSO-d_6).

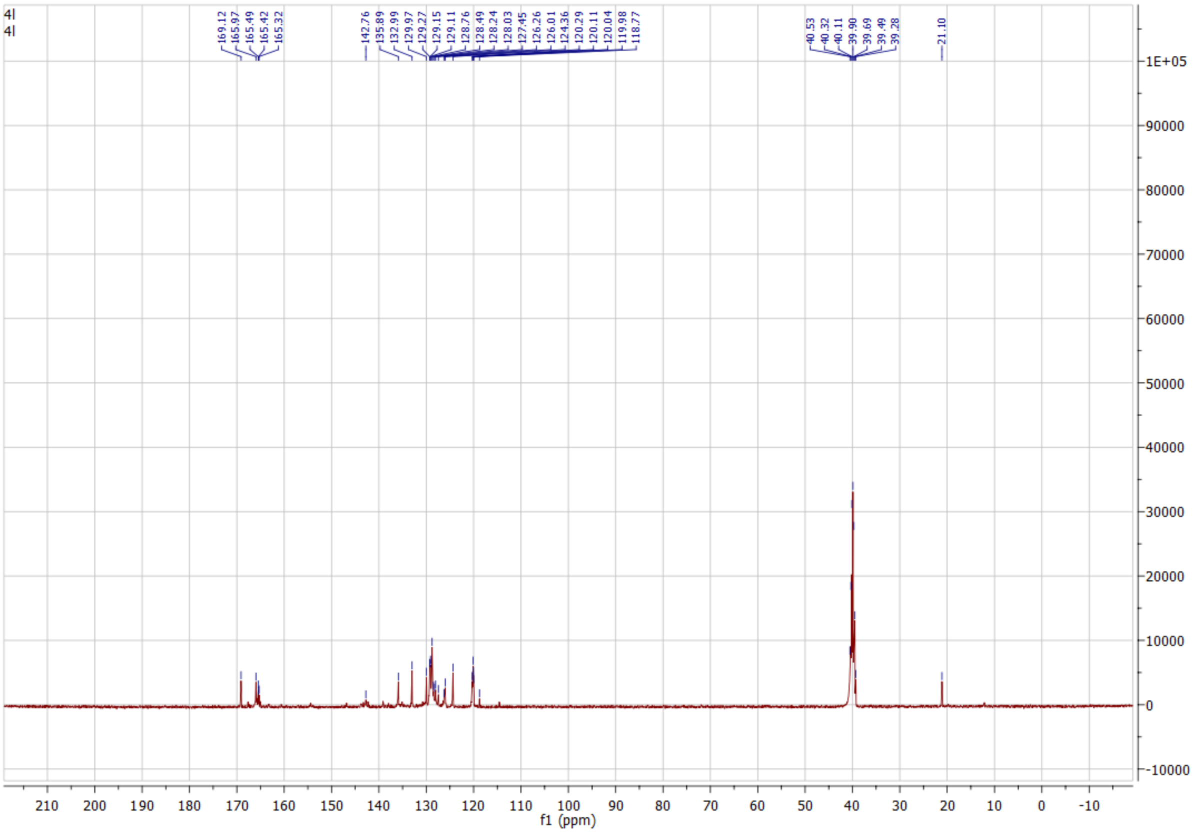

Figure S36. ^{13}C-NMR spectrum for **4l** (100 MHz, DMSO-d_6).

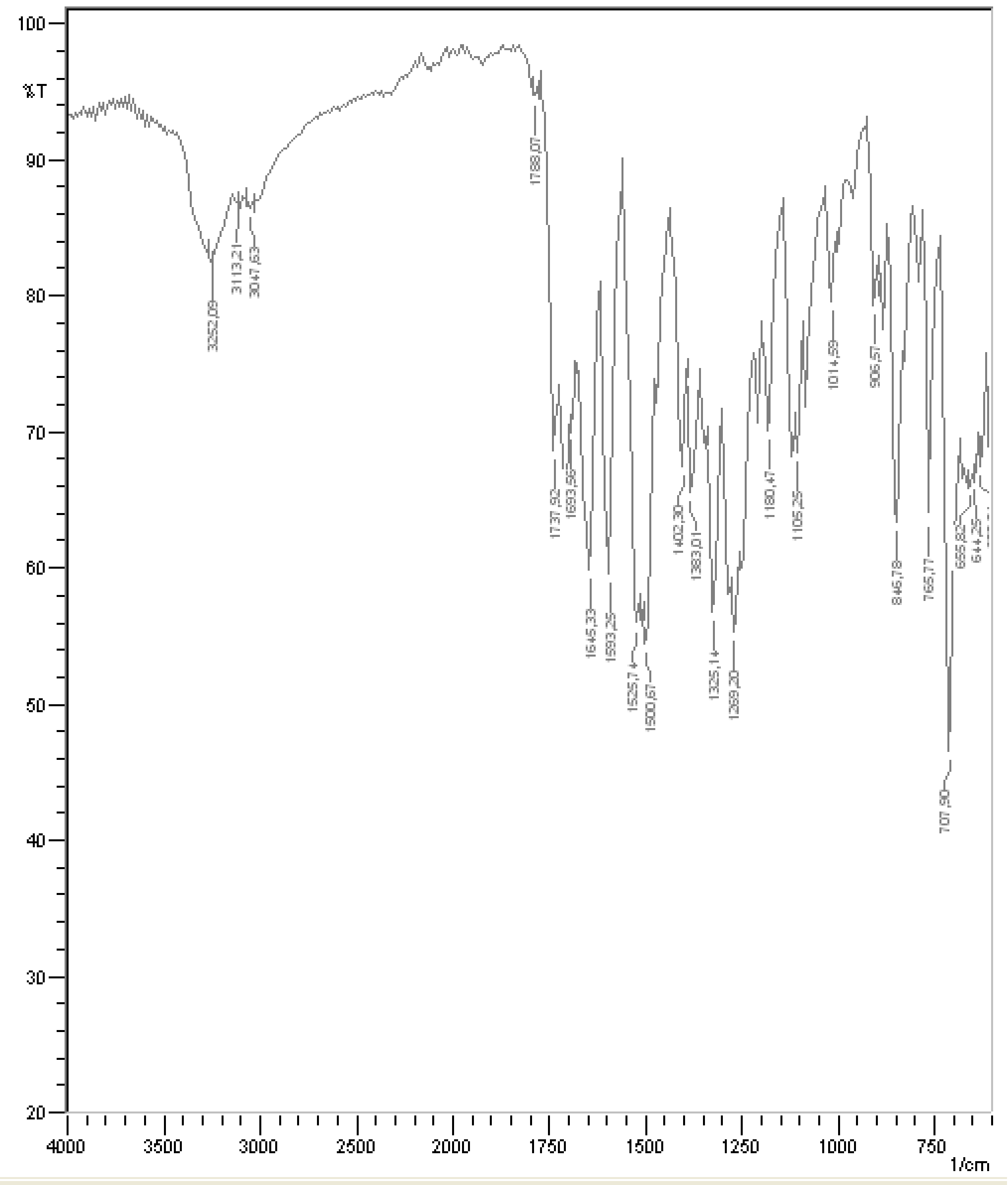

Figure S37. IR spectrum for **4m.**

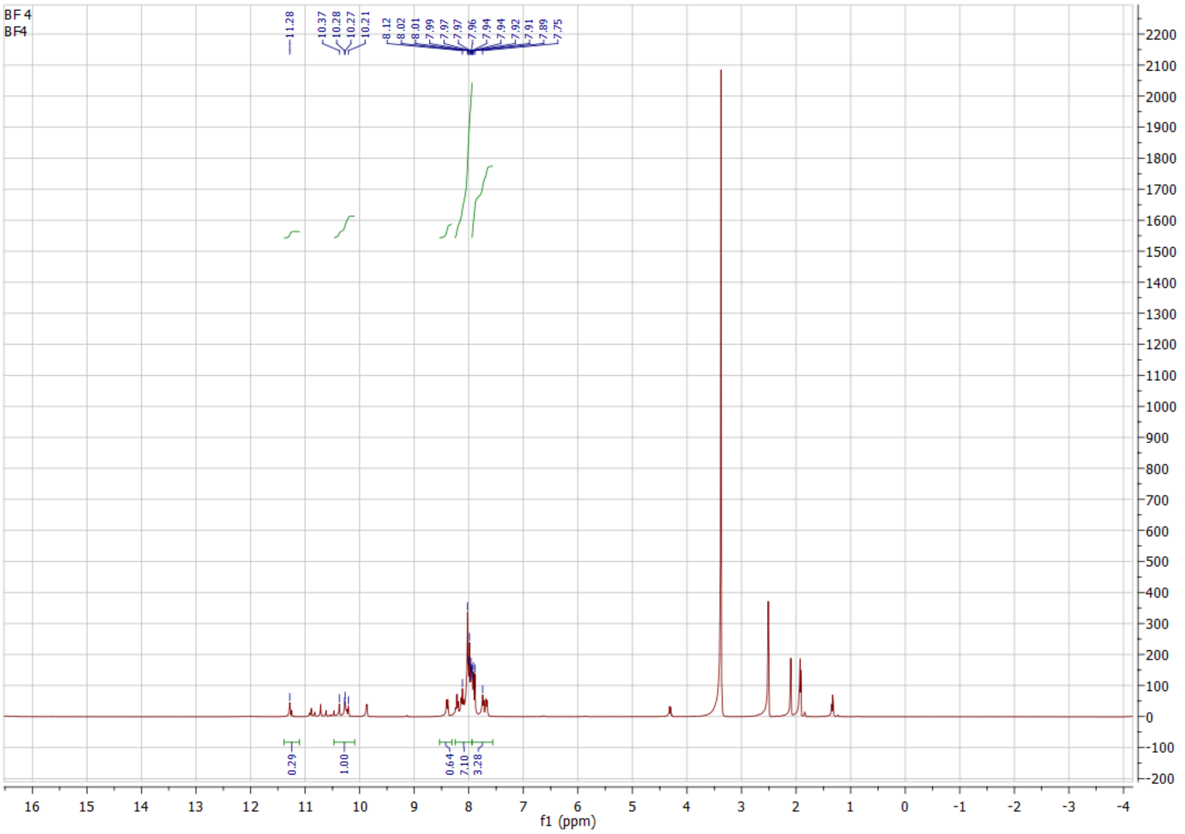

Figure S38. ^{1}H-NMR spectrum for **4m** (400 MHz, DMSO-d_6).

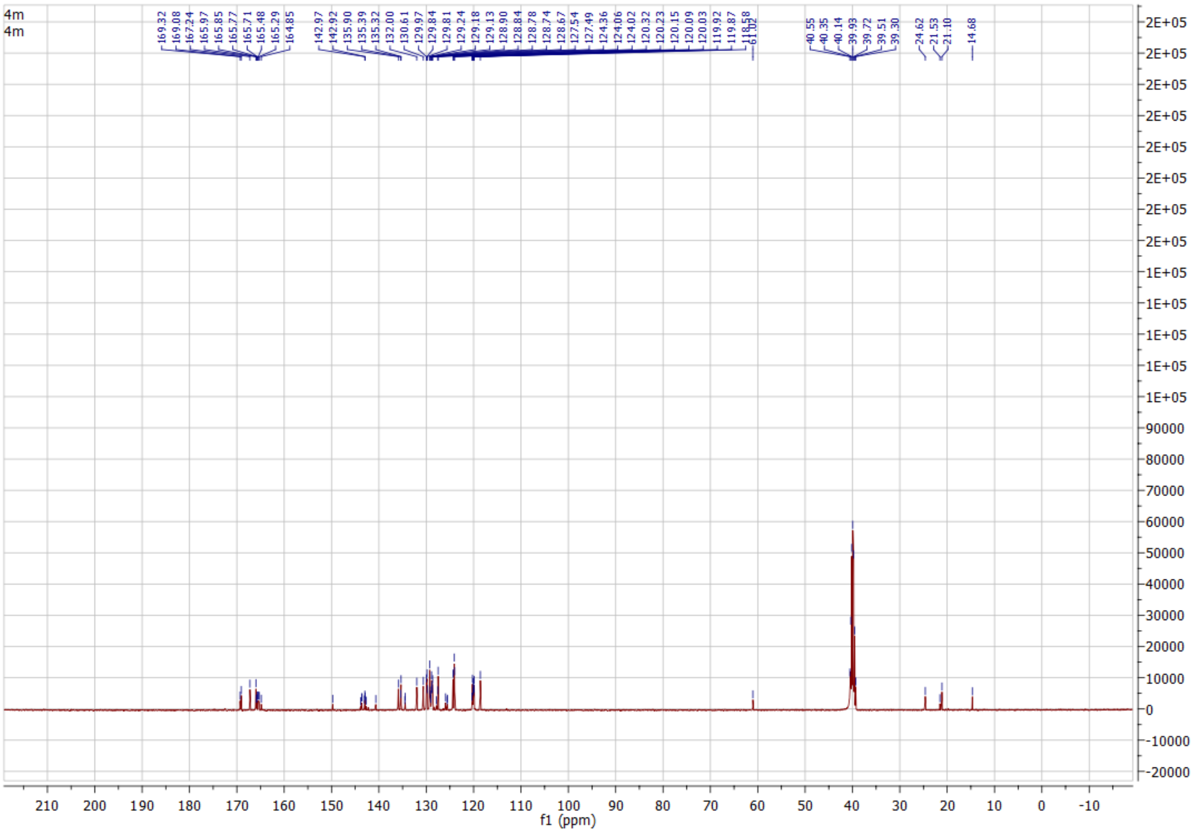

Figure S39. ^{13}C-NMR spectrum for **4m** (100 MHz, DMSO-d_6).

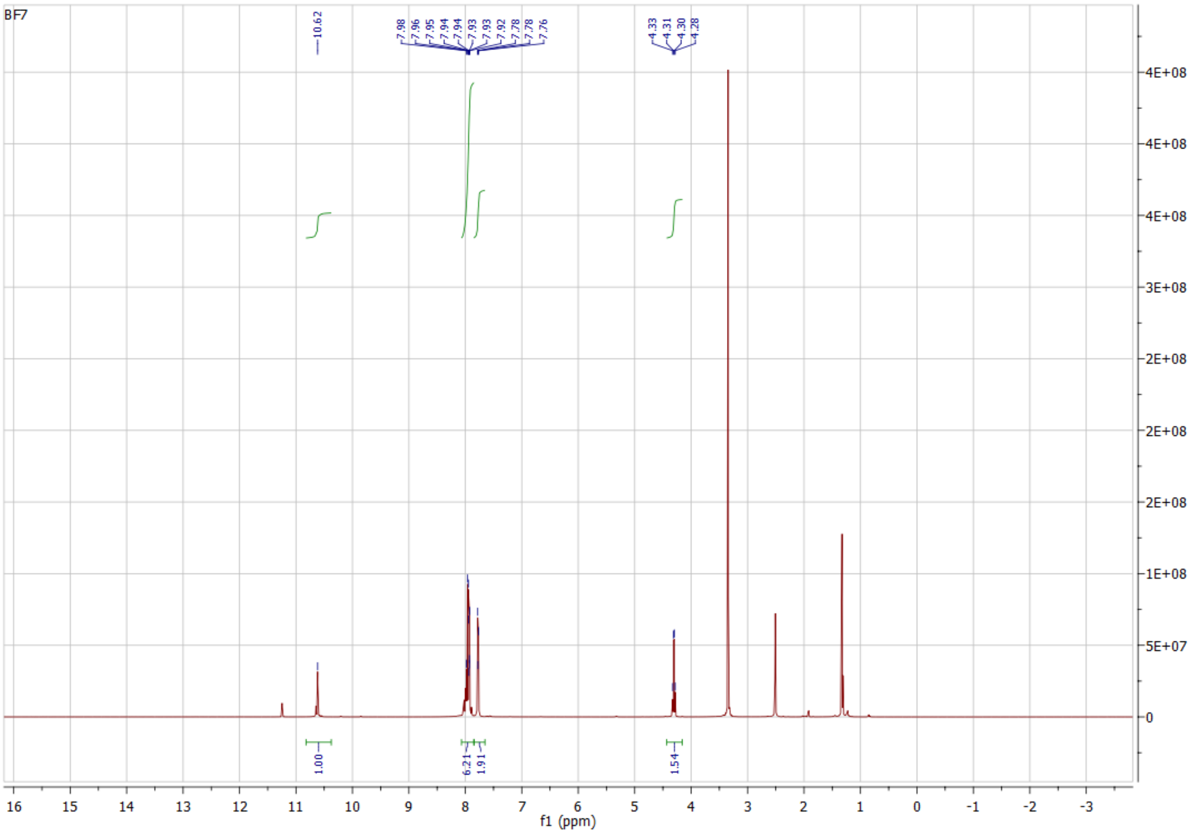

Figure S40. ^{1}H-NMR spectrum for **3f** (500 MHz, DMSO-d_6).

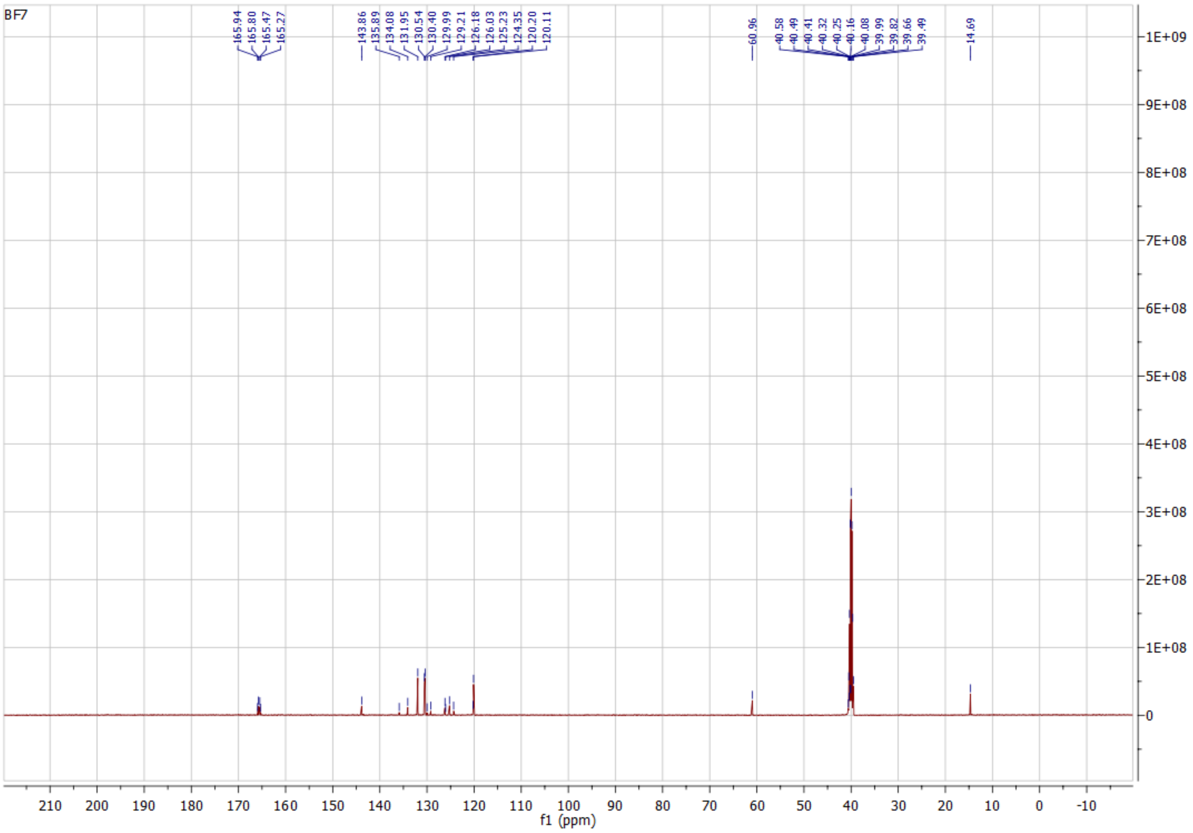

Figure S41. ^{13}C-NMR spectrum for **3f** (125 MHz, DMSO-d_6).

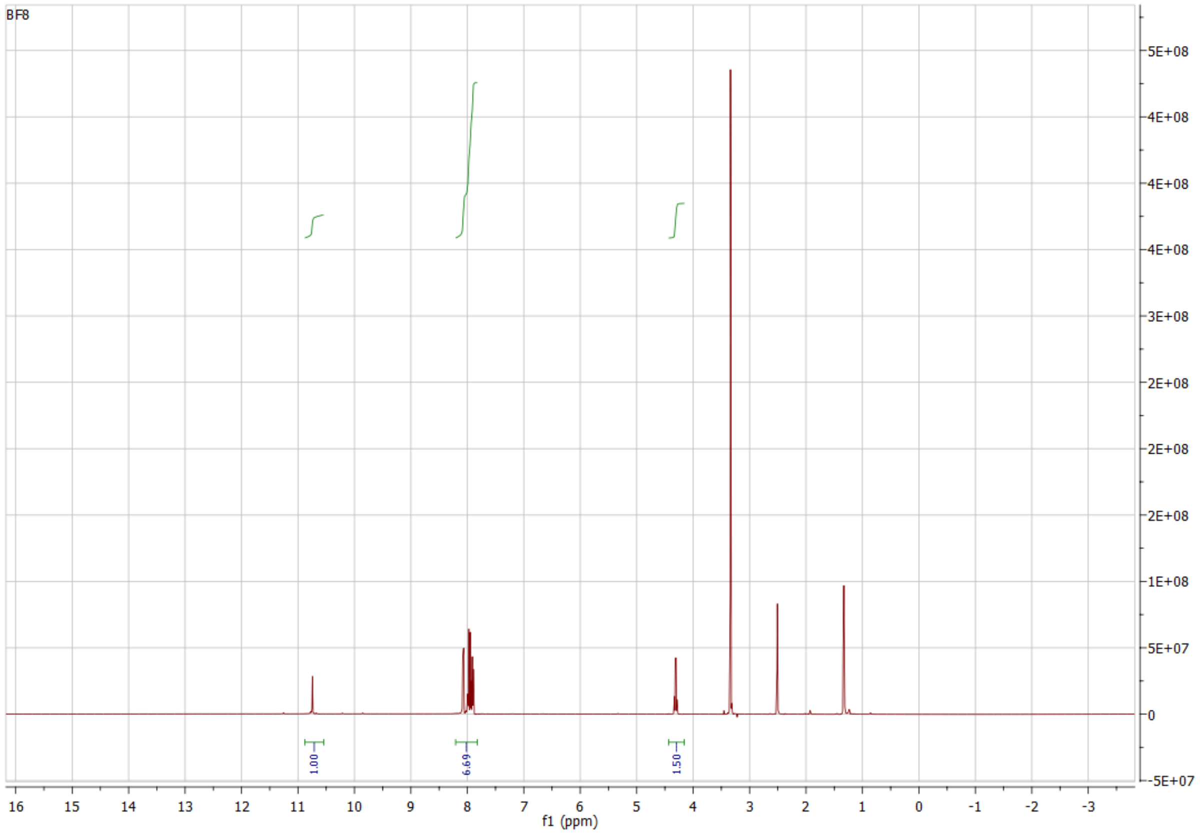

Figure S42. ^{1}H-NMR spectrum for **3g** (500 MHz, DMSO-d6).

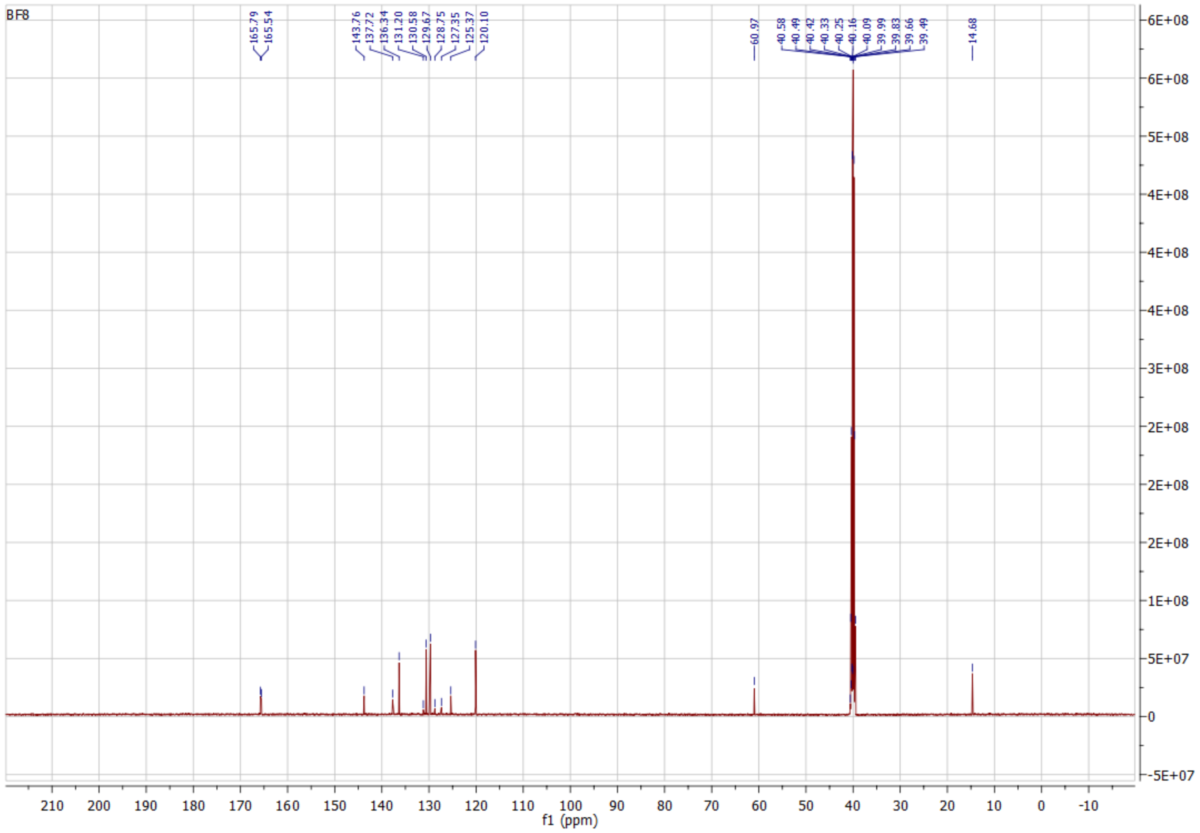

Figure S43. ^{13}C-NMR spectrum for **3g** (125 MHz, DMSO-d6).

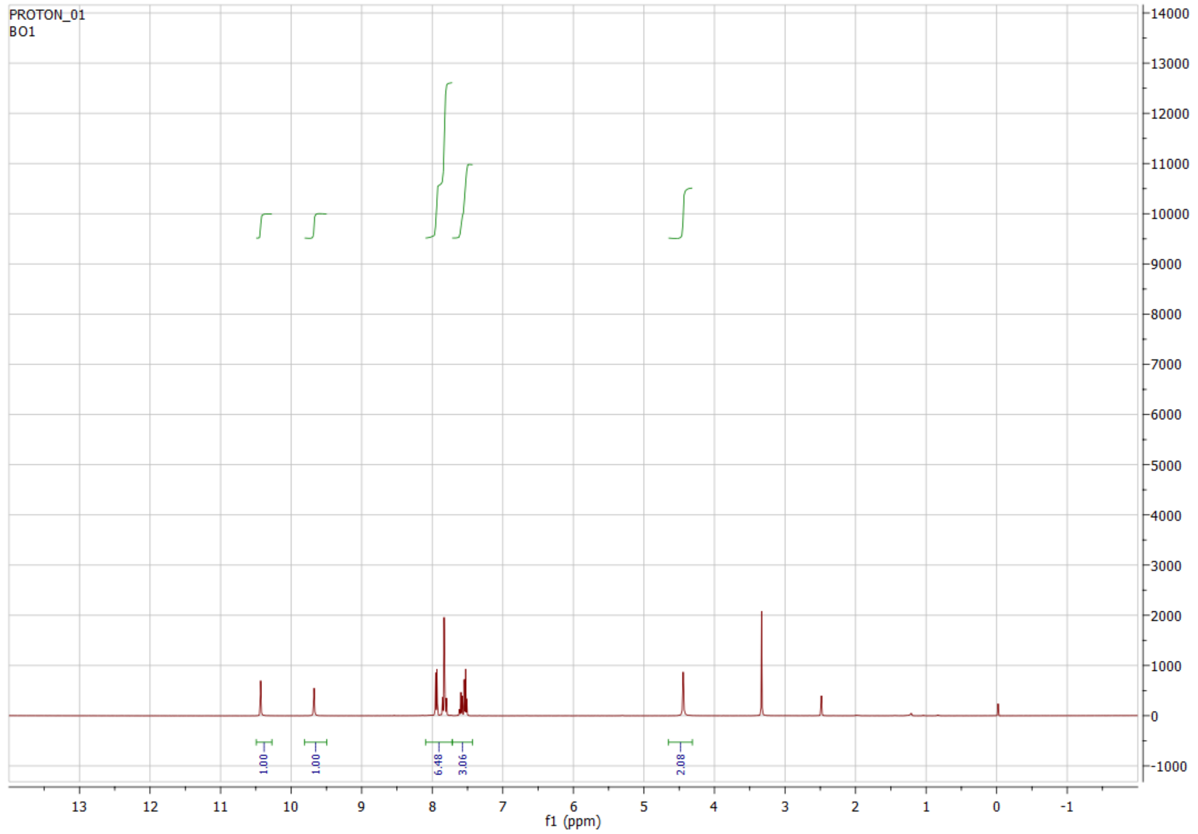

Figure S44. ^{1}H-NMR spectrum for **3h** (500 MHz, DMSO-d_6).

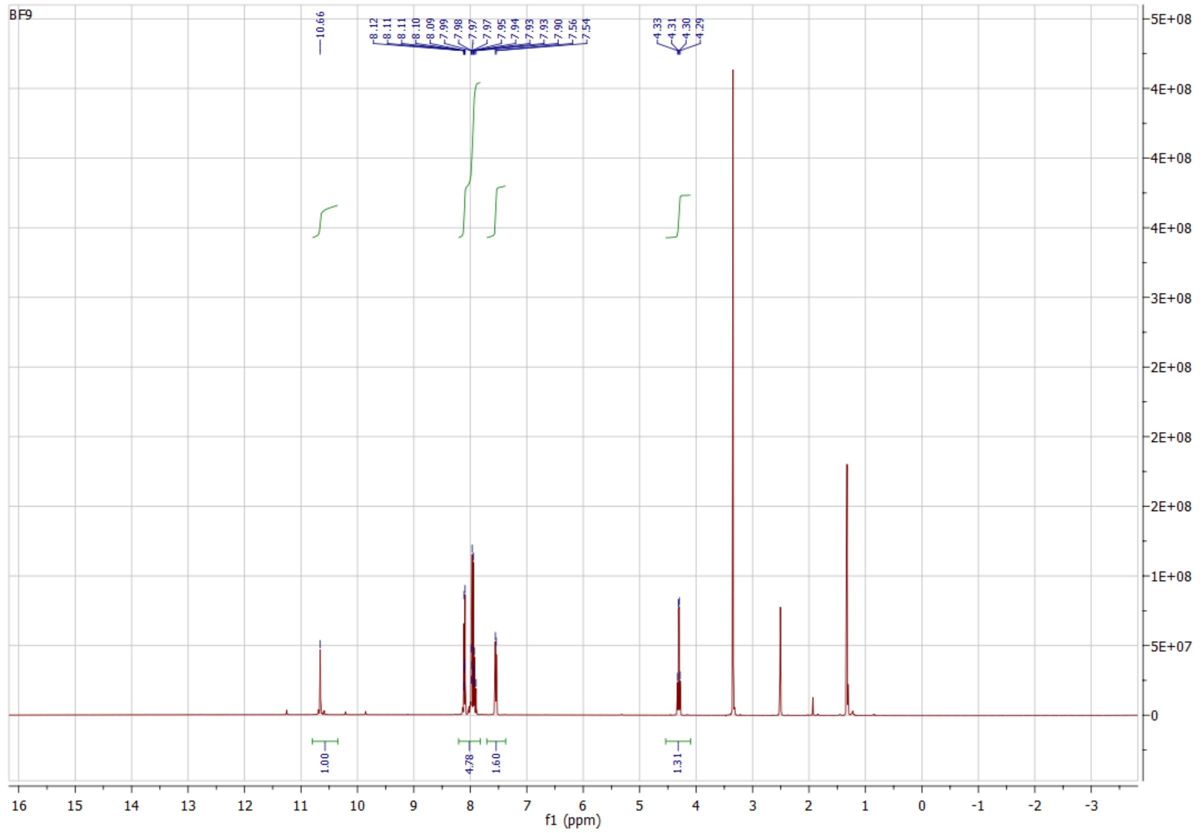

Figure S45. ^{1}H-NMR spectrum for **3i** (500 MHz, DMSO-d_6).

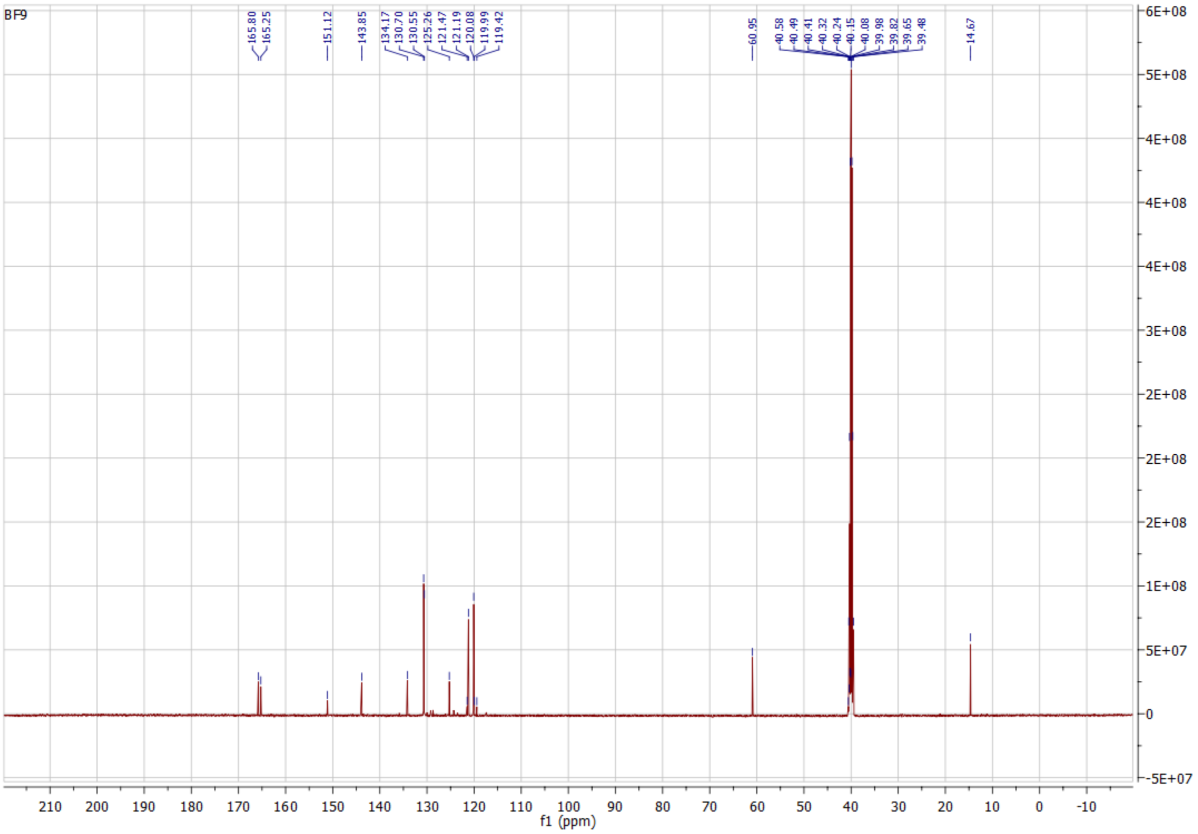

Figure S46. ^{13}C-NMR spectrum for **3i** (125 MHz, DMSO-d$_6$).

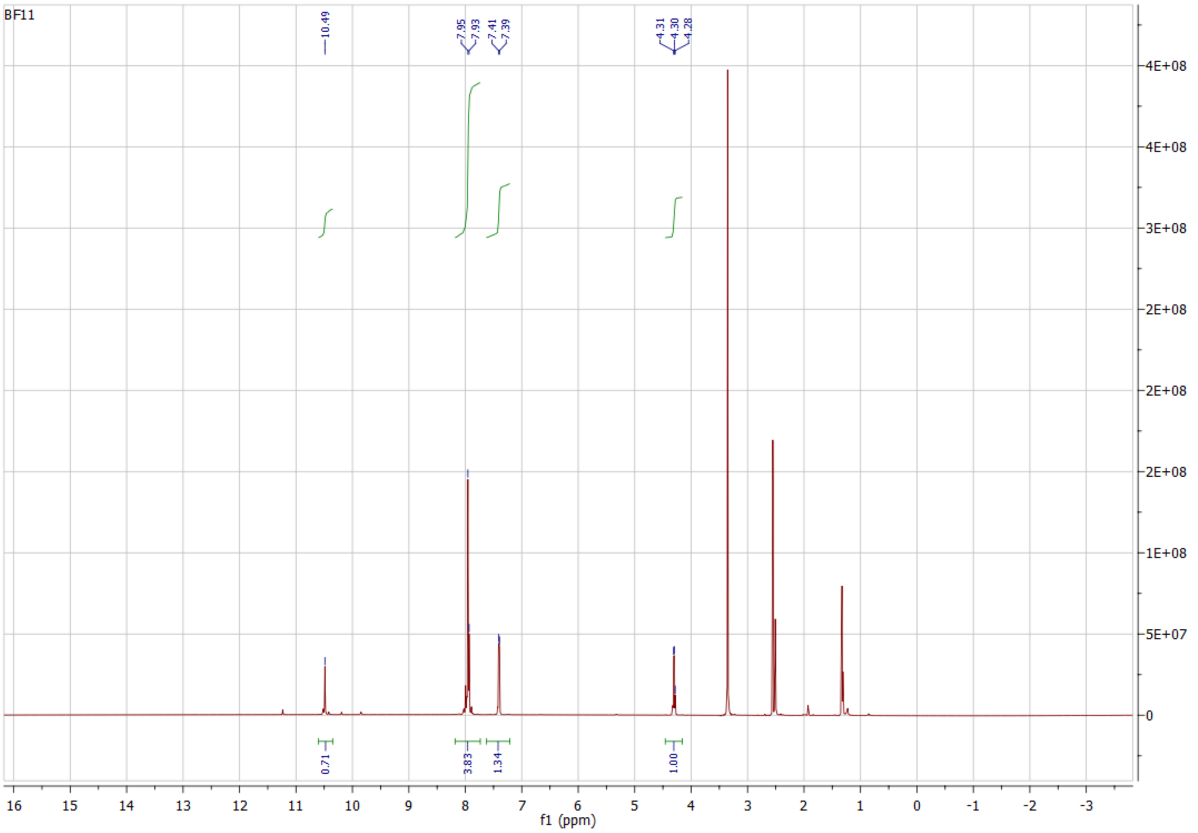

Figure S47. ^{1}H-NMR spectrum for **3j** (500 MHz, DMSO-d$_6$).

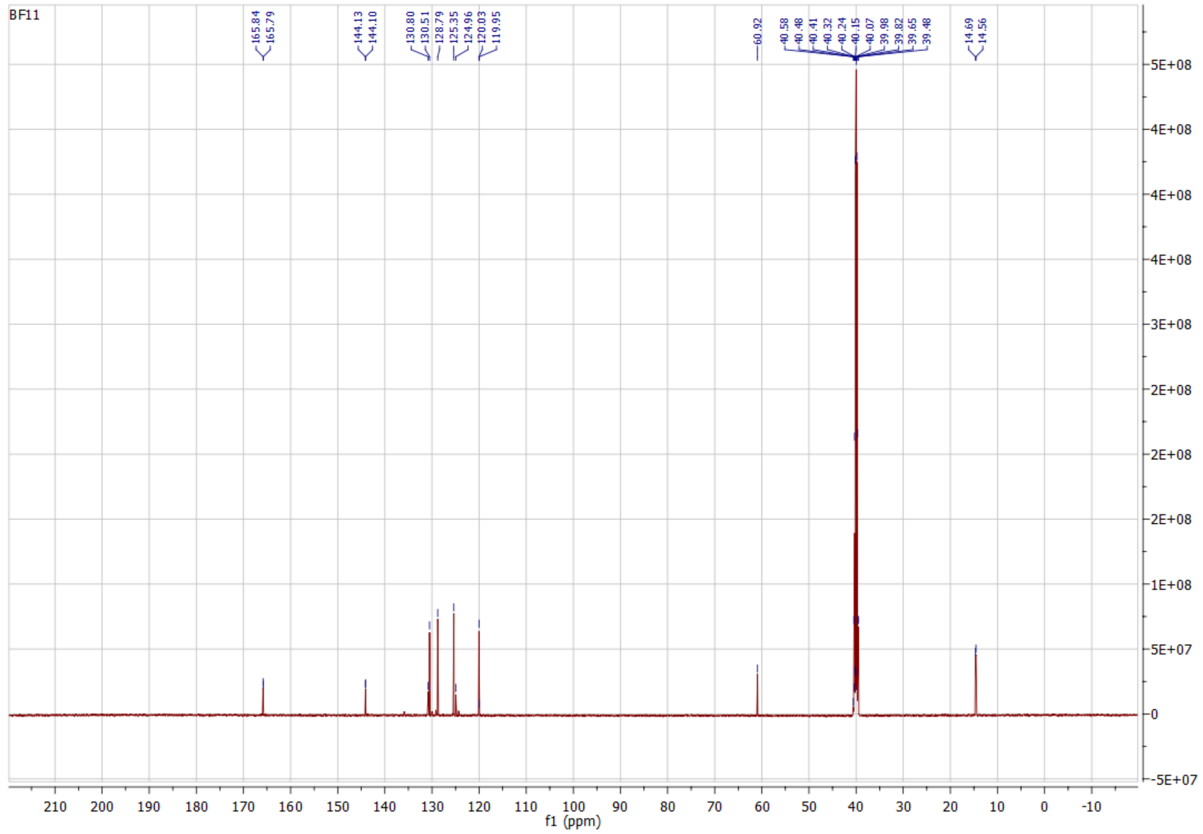

Figure S48. ^{13}C-NMR spectrum for **3j** (125 MHz, DMSO-d_6).

Table S1*. Screening test to evaluation of derivatives (**4a**-**4m**) on mortality of adult female *A. suspensa* under the laboratory conditions at 26±1°C, 70±5% RH, and 12:12 L:D photoperiod.

Derivatives	Dosage, μg/fly	n	Mortality, % (Mean ±SE)
4a	100	60	100.0±0.0
4b	100	59	13.6±1.8
4c	100	60	100.0±0.0
4d	100	60	100.0±0.0
4e	100	60	16.7±6.0
4f	100	59	15.3±0.3
4g	100	59	6.8±1.6
4h	100	60	16.7±6.0
4i	100	60	20.0±2.9
4j	100	59	16.9±7.2
4k	100	60	13.5±1.5
4l	100	60	15.0±2.9
4m	100	60	17.0±3.5
Control		60	3.3±1.7

*The screening tests were conducted following the same procedure described in the toxicity test. A higher dosage at 100 μg per fly of each derivative was used to evaluate the effect on mortality of female *A. suspensa*. In each test, 19~20 female adults were tested and replicated 3 times. Neat acetone was used as control.
